# Mechanistic Insights into the Reaction of Amidines with 1,2,3-Triazines and 1,2,3,5-Tetrazines

**DOI:** 10.1021/jacs.2c03726

**Published:** 2022-06-06

**Authors:** Zhi-Chen Wu, K. N. Houk, Dale L. Boger, Dennis Svatunek

**Affiliations:** † Department of Chemistry, The Scripps Research Institute, La Jolla, California 92037, United States; ‡ Department of Chemistry and Biochemistry, University of California, Los Angeles, Los Angeles, California 90095, United States; § Department of Chemistry, The Skaggs Institute for Chemical Biology, La Jolla, California 92037, United States; ∥ Institute of Applied Synthetic Chemistry, TU Wien, 1060 Vienna, Austria

## Abstract

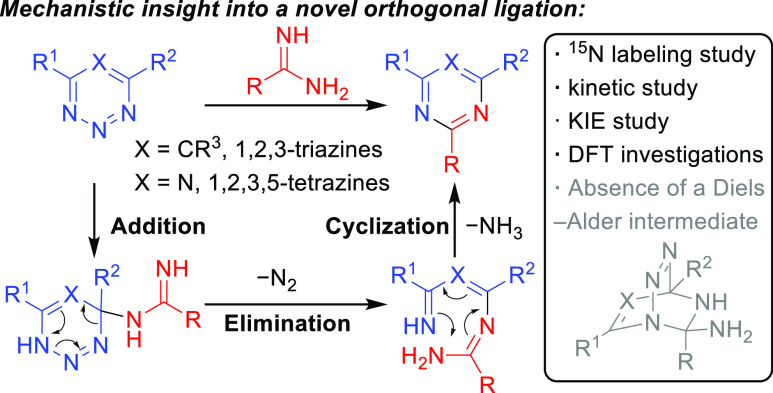

1,2,3-Triazines and 1,2,3,5-tetrazines react rapidly, efficiently, and selectively with amidines to form pyrimidines/1,3,5-triazines, exhibiting an orthogonal reactivity with 1,2,4,5-tetrazine-based conjugation chemistry. Whereas the mechanism of the reaction of the isomeric 1,2,4-triazines and 1,2,4,5-tetrazines with alkenes is well understood, the mechanism of the 1,2,3-triazine/1,2,3,5-tetrazine–amidine reaction as well as its intrinsic reactivity remains underexplored. By using ^15^N-labeling, kinetic investigations, and kinetic isotope effect studies, complemented by extensive computational investigations, we show that this reaction proceeds through an addition/N_2_ elimination/cyclization pathway, rather than the generally expected concerted or stepwise Diels–Alder/retro Diels–Alder sequence. The rate-limiting step in this transformation is the initial nucleophilic attack of an amidine on azine C4, with a subsequent energetically favored N_2_ elimination step compared with a disfavored stepwise formation of a Diels–Alder cycloadduct. The proposed reaction mechanism is in agreement with experimental and computational results, which explains the observed reactivity of 1,2,3-triazines and 1,2,3,5-tetrazines with amidines.

## Introduction

The inverse electron demand Diels–Alder reaction of heterocyclic azadienes serves as a powerful method for the construction of highly substituted or functionalized six-membered heterocyclic systems with widespread applications, including natural product total synthesis^1−3^ and the divergent construction of screening libraries.^4−7^ In particular, the ultrafast reaction between 1,2,4,5-tetrazines and strained alkenes is now developed as a mature and widely applied bioorthogonal conjugation method in chemical biology.^8−12^ Previous contributions in this area resulted in the detailed establishment of the reactivity of each heterocycle class with a variety of dienophiles,^13−17^ and computational studies elucidated the intrinsic reactivities of these heterocycles as well as the impact of substituent effects.^18−23^ Specifically, we explored the cycloaddition reactions between 10 fundamental azadienes and ethylene dienophiles, revealing the origins of their different reactivities from the analysis of both orbital interactions and distortion energies.^19^ Due to the low energetic penalty during distortion to the transition state geometry, 1,2,4,5-tetrazines and 1,2,4-triazines have the highest reactivity among tetrazines and triazines, which is demonstrated by the superb reactivity of these two heterocycles and the extensive exploration of the bioorthogonal ligation reactions of 1,2,4,5-tetrazines.^9,24,25^

Recently, we systematically defined the reactivity of various 1,2,3-triazines and that of the first member of the previously unknown 1,2,3,5-tetrazines as a family of more polarized dienes in inverse electron demand Diels–Alder reactions and demonstrated their applications in the synthesis of various heterocycles including pyridines, pyrimidines, pyridazines, and 1,3,5-triazines.^26−32^ Specifically, we defined the outstanding reactivity between 1,2,3-triazine/1,2,3,5-tetrazine and amidines, providing nearly quantitative conversion to pyrimidines/1,3,5-triazines under mild conditions, demonstrated the exclusive C4/N1 cycloaddition selectivity, and quantitatively determined the rate constants of these reactions that meet the requirements of applicable ligation reactions. The observed orthogonal reactivity between a novel 1,2,3,5-tetrazine/amidine ligation and a traditional 1,2,4,5-tetrazine/strained alkyne ligation revealed a highly distinct reactivity between the two isomeric tetrazines.^26^ A similar discovery was also reported by Siegl and Vrabel, with an observed orthogonality between the 1,2,3-triazine/amidine ligation pair and the 1,2,4-triazine/*trans*-cyclooctene pair.^33^ Whereas 1,2,3,5-tetrazines/1,2,3-triazines are predictably less reactive than 1,2,4,5-tetrazines/1,2,4-triazines with ethylene dienophiles, the source of their superb intrinsic reactivity with heterodienophiles including amidines remains unclear. Our previous investigation of the reaction between 1,2,3-triazines and enamines revealed the reaction course shifting away from a concerted to a stepwise mechanism along with an improved reactivity depending on the triazine substituents as well as the reaction solvent,^34^ shedding light on the impact of a potential alternative reaction mechanism on the outstanding reactivity. Herein, we report our experimental and computational investigation of the mechanism and the intrinsic reactivity of the reaction between amidines and 1,2,3,5-tetrazine/1,2,3-triazines.

## Results and Discussion

**Figure 1:**
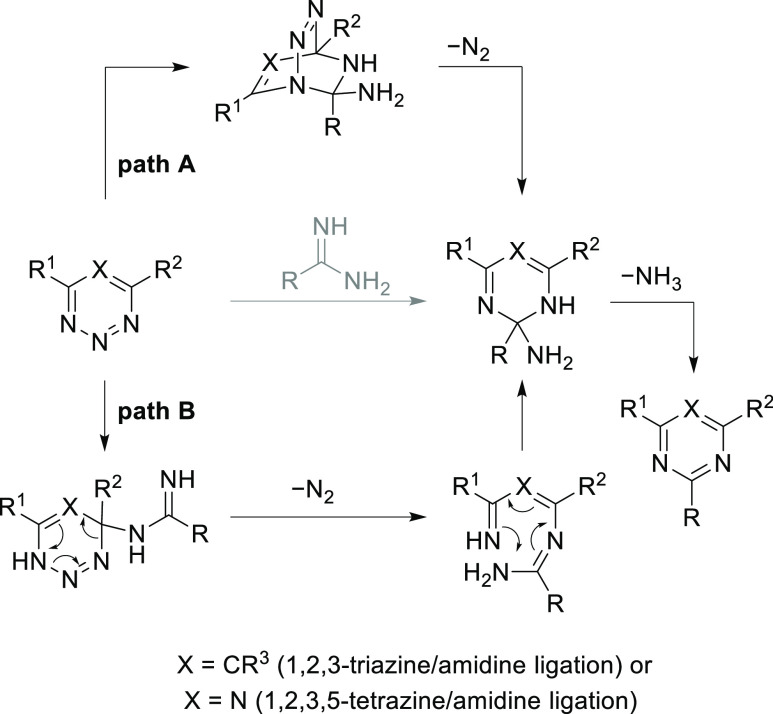
Possible mechanisms for the reaction between 1,2,3-triazines/1,2,3,5-tetrazines with amidines including a Diels–Alder mechanism (path A) and an addition/N_2_ elimination/cyclization mechanism (path B).

Focusing on the mechanism of the reaction between amidines and 1,2,3,5-tetrazine/1,2,3-triazines, the widely accepted pathway is a concerted or stepwise Diels–Alder reaction mechanism, which is then followed by a subsequent retro Diels–Alder reaction and elimination to provide the aromatic 1,3,5-triazine/pyrimidine products (Figure 1, path A). However, due to the increase in the polarity of both reactants, an alternative stepwise addition/N_2_ elimination/cyclization mechanism may also be proposed.^30^ Initiated by a nucleophilic attack of the amidine nitrogen on C4 of the triazine or tetrazine, the adduct could then lose dinitrogen, followed by electrocyclization and elimination to provide the same products (Figure 1, path B).

**Figure 2:**
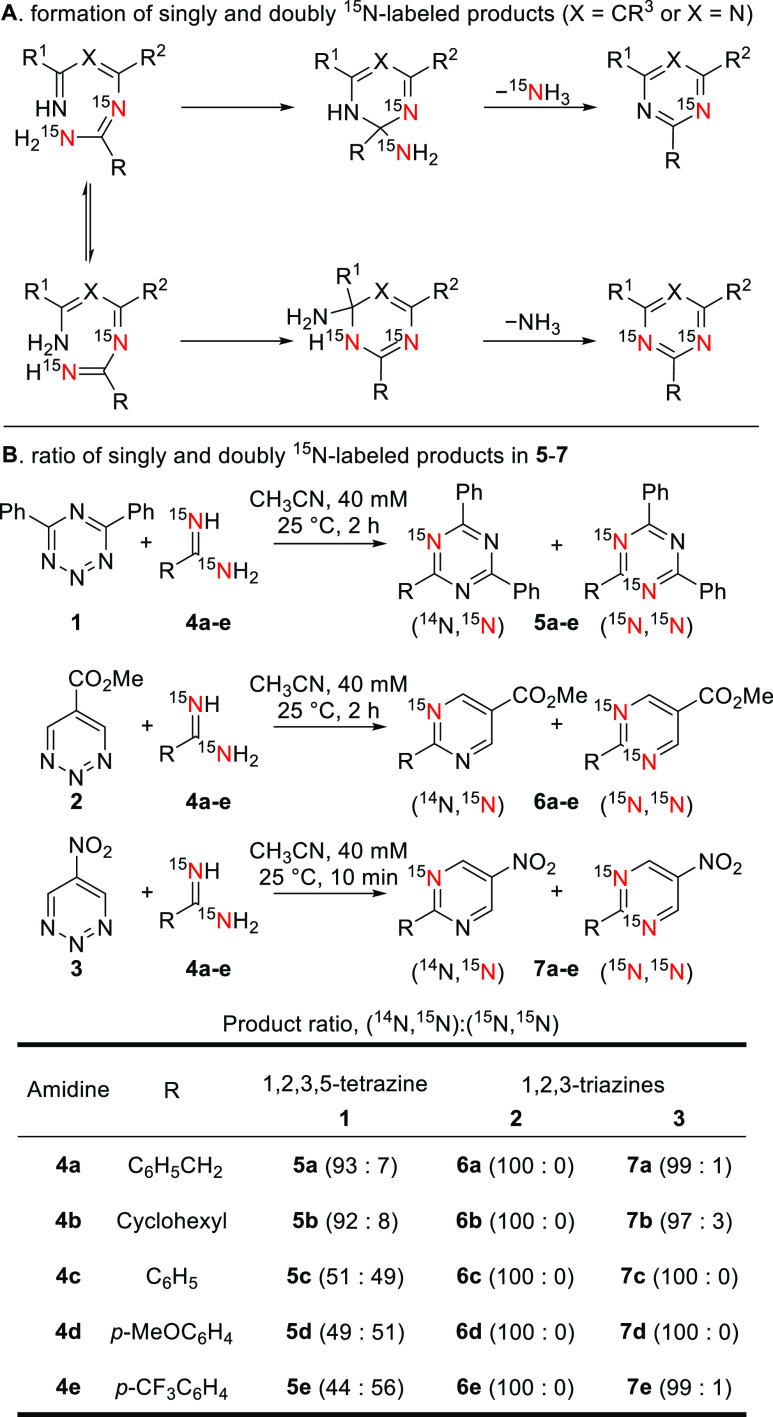
^15^N-labeling studies.

In order to investigate the mechanism of the reaction between 1,2,3,5-tetrazines/1,2,3-triazines and various amidines and distinguish between a concerted or stepwise cycloaddition mechanism (path A) versus a mechanism without the cycloadduct (path B), we expanded our previous ^15^N-labeling studies of the reaction of **1** or **2** with doubly ^15^N-labeled amidine **4a**.^26,30^ Here, we analyzed the reaction product obtained in the reaction of doubly ^15^N-labeled amidines **4a**-**e** and 1,2,3,5-tetrazine **1** or 1,2,3-triazines **2** and **3** (Figure 2). The Diels–Alder mechanism is expected to provide singly ^15^N-labeled products exclusively, whereas two tautomeric 1,3,5-trienes could be present in the alternative addition/N_2_ elimination/cyclization mechanism, providing access to both singly and doubly ^15^N-labeled products (Figure 2A). Similar to the reaction with non-labeled amidine counterparts, the labeling reaction was performed under mild and dilute conditions to provide 1,3,5-triazine products (**5a**–**e**) or pyrimidine products (**6a**–**e**, **7a**–**e**) in nearly quantitative yields (86–99%, Figure S1). The ratios of singly and doubly ^15^N-labeled products were then determined by high-resolution mass spectrometry (HRMS). For 1,3,5-triazine products **5a**–**e** for which a substantial quantity of doubly ^15^N-labeled product was observed, the second ^15^N incorporation ratio was further confirmed through the analysis of the ^13^C NMR spectra, focusing on the carbon peaks split by a ^2^*J*(^13^C–^15^N) coupling. The second ^15^N incorporation ratio is consistent across the two methods, with an observed <5% difference. For pyrimidines **6a**–**e** and **7a**–**e**, the splitting patterns of ^13^C signals revealed that no doubly ^15^N-labeled pyrimidines were observed, further confirming the results from HRMS.

Whereas the reactions between tetrazine **1** and alkyl amidines **4a** or **4b** provided a 7–8% incorporation of the second ^15^N in the triazine products, the reaction between **1** and aryl amidines (**4c**–**e**) resulted in a substantial 49–56% incorporation of the second ^15^N. While the second, but minor, ^15^N incorporation in the triazine products **5a** and **5b** still provided an ambiguous observation that could be consistent with both mechanisms, a nearly 1:1 ratio of the two labeled products in **5c**–**e** clearly rules out the concerted or stepwise cycloaddition mechanism, suggesting that the reaction does not progress through the cycloaddition intermediate. Notably, the ratios of labeled products were not found to be significantly impacted by either the sterics of the alkyl amidine substituents or the electronics of substituted benzamidines (**5c**–**e**). The ^15^N-labeling studies of the reactions between 1,2,3-triazines (**2** and **3**) and amidines **4a**-**e** provided exclusively singly ^15^N-incorporated triazine products (**6a**–**e** and **7a**–**e**, second ^15^N incorporation 0–3%), differing in the reaction outcome from the reactions of 1,2,3,5-tetrazine. The observed difference in the labeling studies is interesting and intriguing, requiring our further investigation into the reaction mechanism.

**Figure 3:**
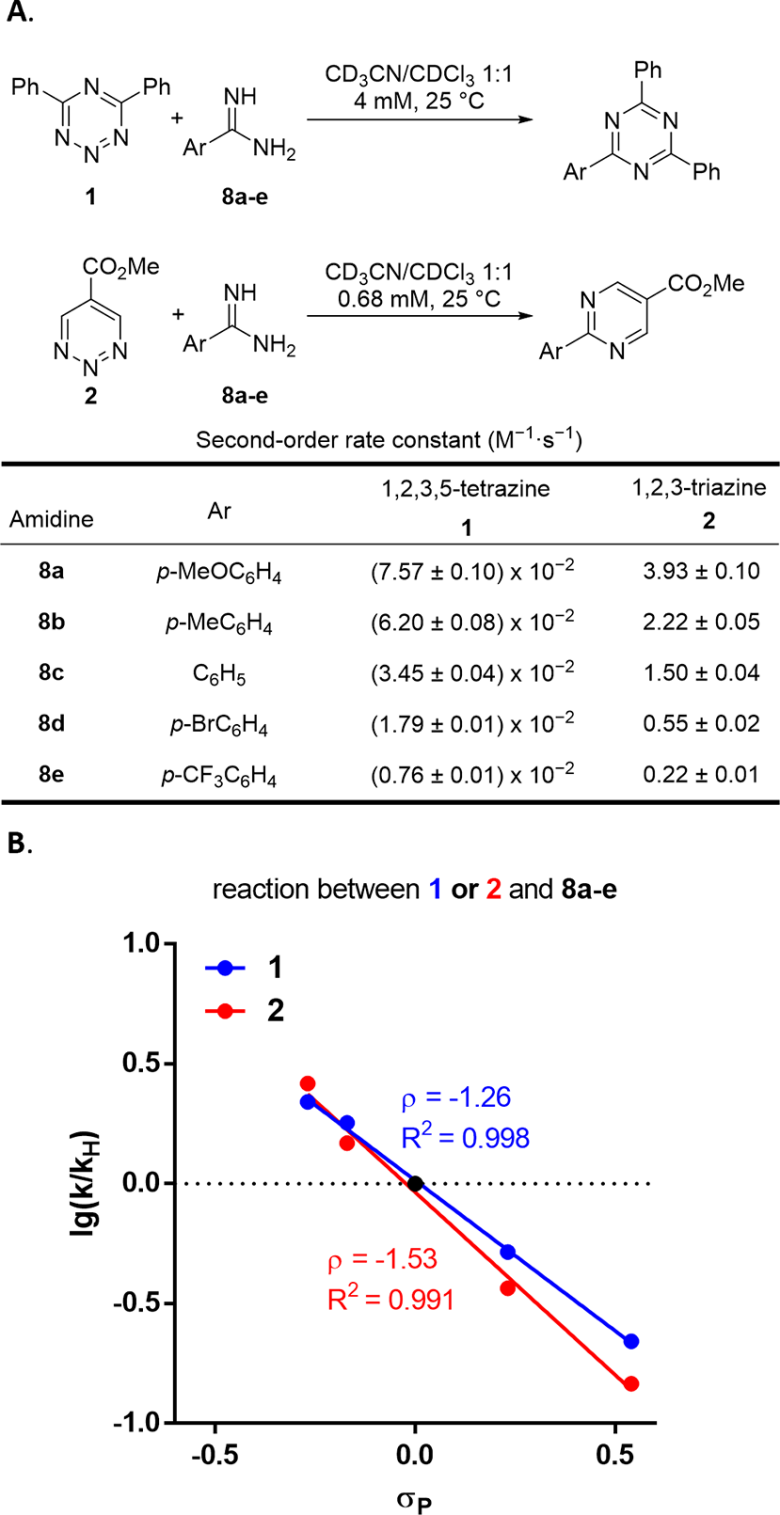
(A) Second-order rate constants of the reactions between tetrazine **1** or triazine **2** and amidines **8a–e**. (B) Hammett plot for the reaction between **1** or **2** and **8a–e**.

Our subsequent investigation focused on the reaction kinetics and the substituent electronic effects of the reactions to further probe the mechanism through a quantitation of reaction rates of tetrazine **1** and triazine **2** (Figure 3). The reaction of tetrazine **1** or triazine **2** with a series of para-substituted benzamidines **8a**–**e** was monitored by ^1^H NMR (solvent: CD_3_CN/CDCl_3_ 1:1), and conversion was determined by observing either the formation of corresponding 1,3,5-triazine or pyrimidine products or the consumption of reactants. The data acquired were observed to fit second-order reaction kinetics from which the rate constants were then determined.

Through ^1^H NMR monitoring of the reaction, a clean formation of the products was observed without the observation of any reaction intermediates (Supporting Information, Figures S2–S11), indicating that the first step in the reaction pathway is the rate-limiting step in both the 1,2,3,5-tetrazine/amidine and 1,2,3-triazine/amidine reactions. A large electronic effect of the para-substituents on the benzamidines **8a**–**e** on the reaction was observed; benzamidines with electron-donating substituents (**8a**, **8b**) provided a greater reactivity and those with electron-withdrawing substituents (**8d**, **8e**) reacted more slowly. The difference in reactivity was as large as 10- to 15-fold between the most electron-rich amidine **8a** and the most electron-deficient amidine **8e**. The relative reaction rates were found to fit a linear Hammett plot, where a ρ value of −1.26 was observed for 1,2,3,5-tetrazine **1**, and a slightly larger ρ value of −1.53 was observed for 1,2,3-triazine **2**. These observations indicate the accumulation of a partial positive charge on the amidine carbon in the transition state of the rate-determining step and support the reaction being initiated by a nucleophilic attack of the amidine nitrogen onto tetrazine **1** or triazines.

With these experimental insights in hand, we proceeded with a computational study of these systems. Using density functional theory (DFT), we employed the M06-2X density functional augmented with Grimme’s D3 dispersion correction as proposed by Grimme and co-workers.^35^ DFT calculations were performed using Gaussian 09 Rev D.01. def2TZVP was used as the basis set. The SMD model with acetonitrile as solvent was used to include the solvent effects. Conformer searches of all involved structures, including transition states, were conducted. A detailed description of the methods used can be found in the Supporting Information.

**Figure 4:**
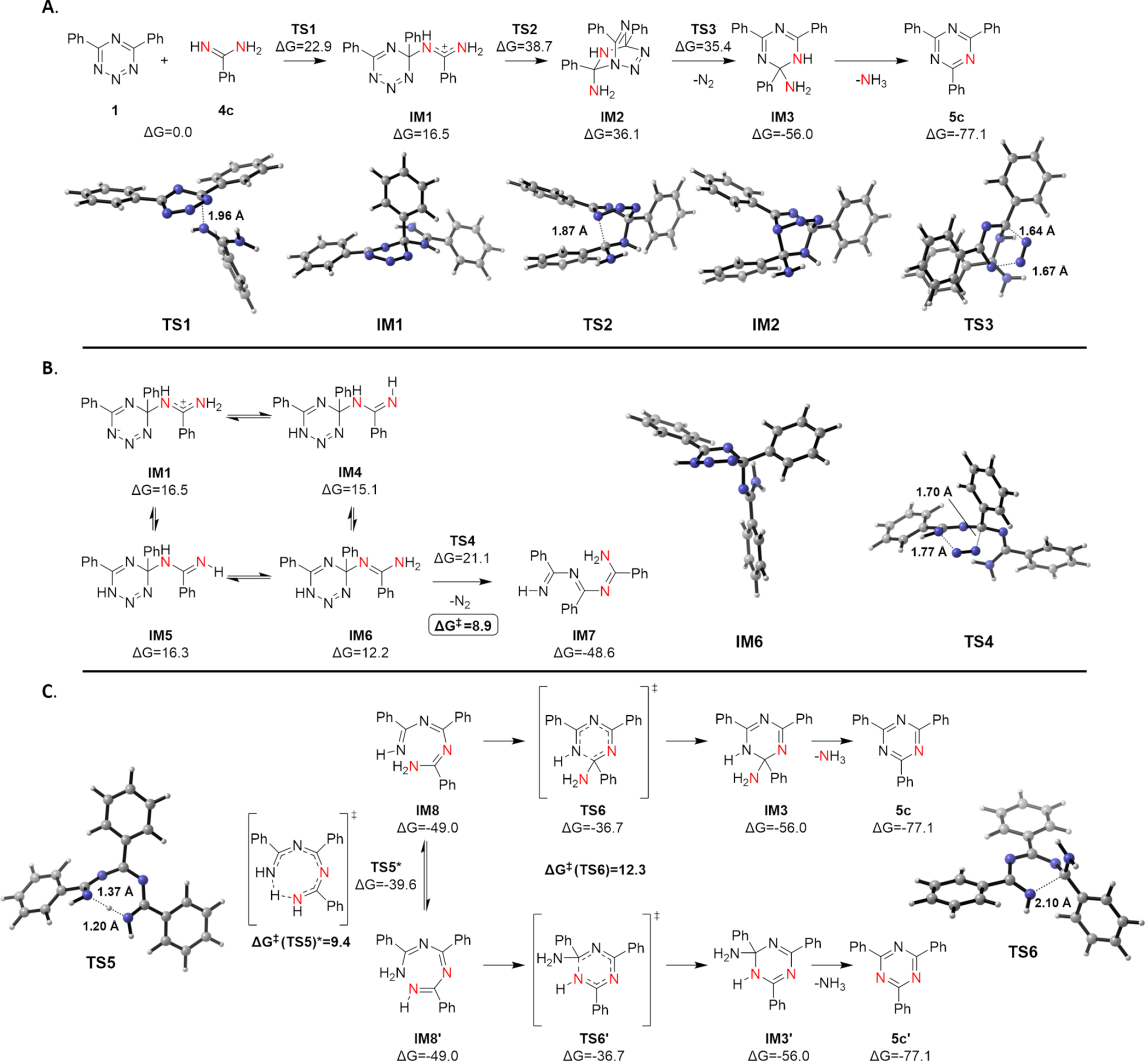
(A) Diels–Alder mechanism for the reaction between **1** and **4c**. (B) Retro Diels–Alder step of the addition/N_2_ elimination/cyclization pathway between **1** and **4c**. (C) Electrocyclization step of the addition/elimination/cyclization pathway between **1** and **4c**. Lowest energy pathways and 3D geometries of key structures are shown. Energies are in kcal/mol relative to starting materials. *Barrier height was corrected to account for tunneling.

Starting with the reaction between **1** and **4c**, we first investigated the Diels–Alder mechanism (Figure 4A). A concerted Diels–Alder transition state could not be located, which is not surprising given the polarized nature of both the diene and dienophile. The initial nucleophilic attack of the amidine nitrogen at C4 of tetrazine **1** has a free energy barrier of 22.9 kcal/mol (**TS1**), leading to a zwitterionic intermediate **IM1**, which is 16.5 kcal/mol higher in energy than the starting material. Formation of the second bond is highly unfavored with a transition state **TS2** at 38.7 kcal/mol, and the energy of Diels–Alder product **IM2** is at 36.1 kcal/mol in relation to the starting material. The subsequent retro Diels–Alder reaction is barrierless (**TS3**) and gives a stable intermediate **IM3** accompanied by the elimination of N_2_, and the final product **5c** is provided after elimination of NH_3_. For tetrazine **1**, the ratio between singly and doubly ^15^N-labeled products in the reaction with doubly ^15^N-labeled amidine **4c** is close to 1:1. This experimental result already excludes the Diels–Alder mechanism and is further supported by our calculations due to the high free energies encountered in this pathway.

Next, we investigated the addition/N_2_ elimination/cyclization pathway. The first step is a nucleophilic attack identical to the Diels–Alder mechanism, where the zwitterionic intermediate **IM1** formed is in equilibrium with three other tautomers (**IM4**–**6**, Figure 4B).

The most stable intermediate **IM6** corresponds to the lowest energy transition state (**TS4**) among the tautomers in the subsequent retro Diels–Alder reaction to provide highly stable **IM7** accompanied by the extrusion of N_2_. **IM1**, **IM4**, and **IM5** can also undergo the retro-Diels–Alder reaction, leading to tautomers of **IM7**, albeit with higher barriers (see Figure S12). Amongst all the possible tautomeric and rotational isomers of **IM7**, isomers **IM8** and **IM8′** are the only ones that can react in the 6π electrocyclization (Figure 4C), which can rapidly interconvert between each other by intramolecular proton transfer through the low-energy transition state **TS5** (corrected for tunneling, see Figure S14). However, these two intermediates are the same due to symmetry, and only the usage of ^15^N-labeled amidine **4c** or amidines different from **4c** as the reaction partner differentiate between these tautomers. The electrocyclization products **IM3** and **IM3′** are hence expected to be generated in equal amounts since both electrocyclization pathways are equivalent; a subsequent elimination of NH_3_ provides both singly and doubly ^15^N-labeled triazines **5c** in a 1:1 ratio as the overall product of the reaction. Consequently, such a stepwise reaction mechanism through intermediates **IM8** and **IM8′** is in good agreement with our ^15^N-labeling studies where a 49:51 ratio was observed for product **5c**. The comparison of the activation energy of all steps and the relative energy of all transition states supports the initial nucleophilic addition as the rate-limiting step, which is also in accordance with monitoring of the reaction progress between 1,2,3,5-tetrazine **1** and aryl amidines **8a–e** as well as the observed Hammett relationship (Figure 3B).

**Figure 5:**
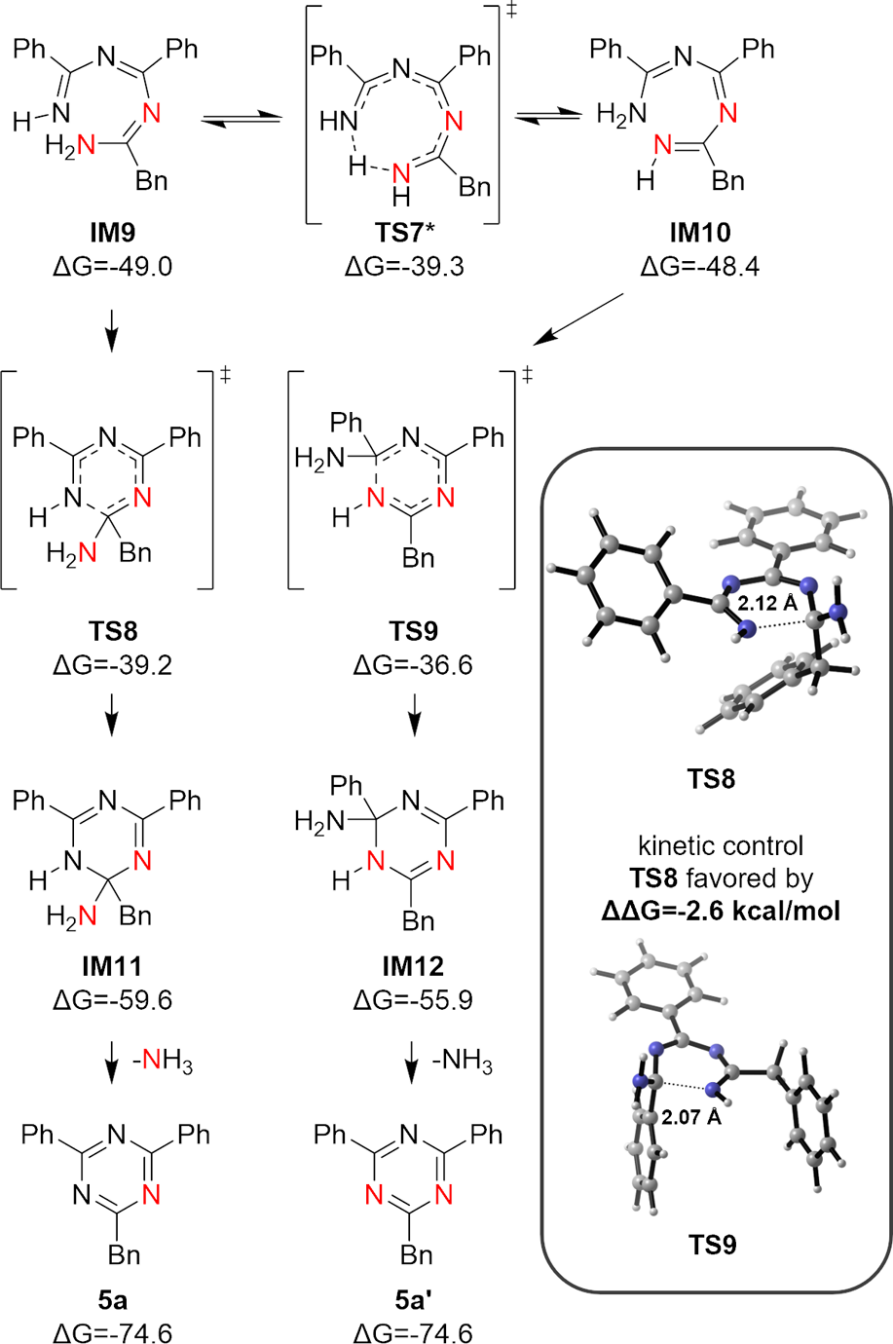
Electrocyclization step of the addition/N_2_ elimination/cyclization pathway between **1** and **4a**. Lowest energy pathway and 3D geometries of key structures are shown. Energies are in kcal/mol relative to starting materials. *Barrier height was corrected to account for tunneling.

A more general case was also investigated in detail in addition to the 1,2,3,5-tetrazine ligation reaction with amidine **4c** to shed light on the reaction mechanism with a non-symmetrical 6π-electrocyclization precursor with two possible electrocyclization pathways. Focusing on the selectivity-determining electrocyclization step between **1** and **4a**, the electrocyclization precursors **IM9** and **IM10** are close in energy with **IM10** at a relative energy of +0.6 kcal/mol (Figure 5); the interconversion between the two tautomers is also possible through **TS7**. This intramolecular proton transfer is accelerated due to tunneling (Figure S14). Electrocyclization starting from **IM9** through **TS8** was found to be kinetically favored by −2.6 kcal/mol over the alternative **TS9**, resulting in the formation of **IM11** as the major cyclized intermediate over **IM12**. The final elimination of NH_3_ provides both triazines **5a** and **5a′**, with the major product being the singly labeled product **5a** under kinetic control. The predicted reaction outcome agrees with the experimental result where the reaction between 1,2,3,5-tetrazine **1** and ^15^N-labeled phenylacetamidine **4a** provided triazine **5a** in a 93:7 ratio of singly and doubly ^15^N-labeled products.

**Figure 6:**
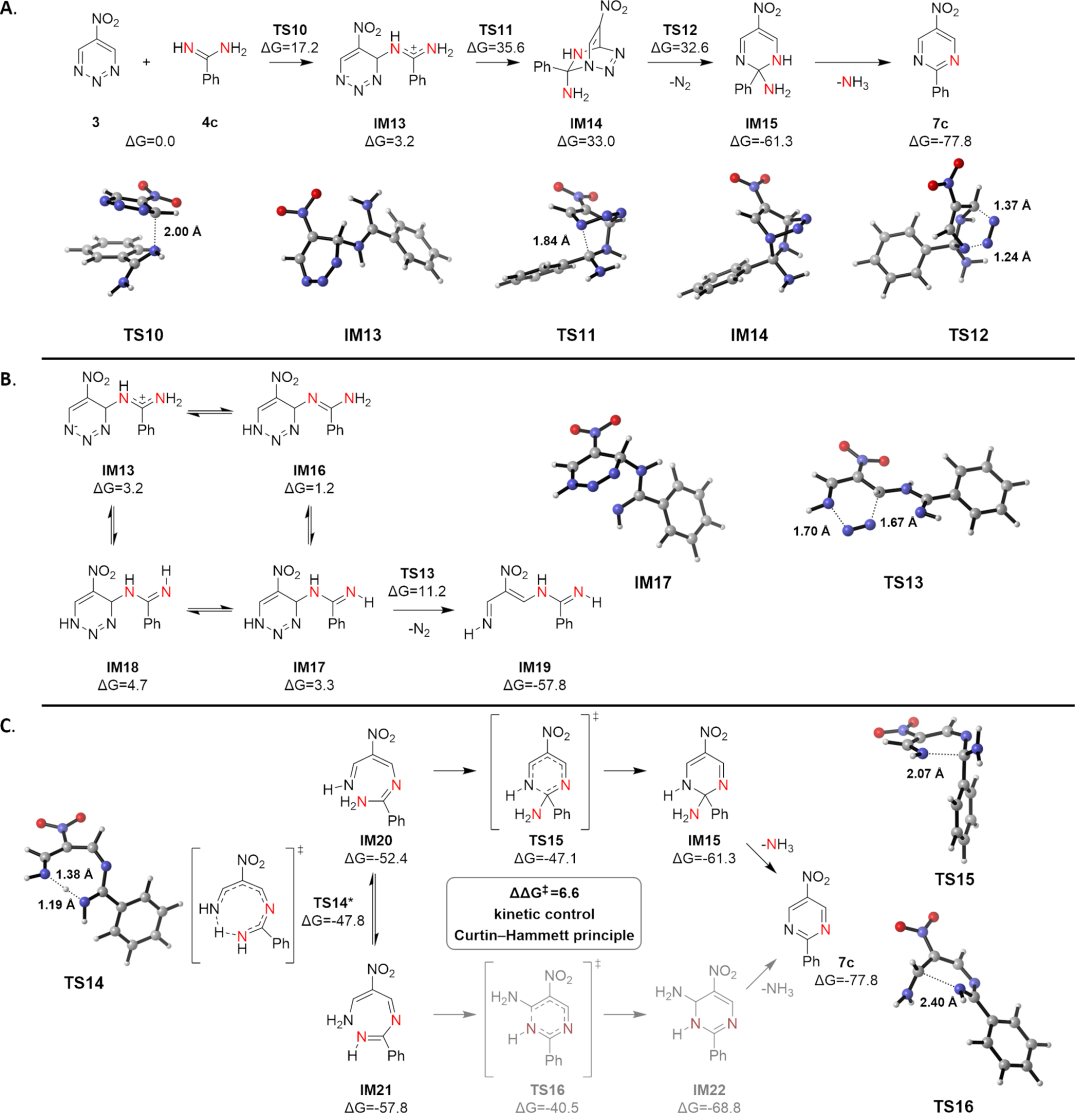
(A) Diels–Alder mechanism for the reaction between **3** and **4c**. (B) Retro Diels–Alder step of the addition/N_2_ elimination/cyclization pathway between **3** and **4c**. (C) Electrocyclization step of the addition/elimination/cyclization pathway between **3** and **4c**. Lowest energy pathways and 3D geometries of key structures are shown. Energies are in kcal/mol relative to starting materials. *Barrier height was corrected to account for tunneling.

Following our studies on the reaction with 1,2,3,5-tetrazines, we then investigated the mechanism of the reaction between 1,2,3-triazine **3** and amidine **4c**. Triazine **3** instead of **2** was chosen for its additional symmetry, avoiding an additional twofold increase in the already very high number of investigated conformers/tautomers. Similar to the case of 1,2,3,5-tetrazine, a concerted Diels–Alder transition state could not be located. In the stepwise Diels–Alder pathway (Figure 6A), the initial nucleophilic attack of the amidine nitrogen at C4 of triazine **3** shows a free energy barrier of 17.2 kcal/mol (**TS10**), leading to a zwitterionic intermediate **IM13**, which is 3.2 kcal/mol higher in energy than the starting materials. Formation of the second bond is highly unfavored with a transition state **TS11** at 35.6 kcal/mol, and the energy of Diels–Alder product **IM14** is 33.0 kcal/mol in relation to the starting material. The subsequent retro Diels–Alder reaction is barrierless (**TS12**) and gives a more stable intermediate **IM15** accompanied by elimination of N_2_, and the final product **7c** is provided after elimination of NH_3_. While the Diels–Alder pathway is in agreement with the result of ^15^N-labeling experiments, the rate-limiting step was found to be the formation of the Diels–Alder cycloadduct **IM14**, which is contradictory to the observed Hammett relationship in a similar triazine **2**. The high barrier encountered in the formation of the bicyclic Diels–Alder product **IM14** further supported an alternative mechanism not including the energetically highly disfavored cycloadduct.

Next, we investigated the addition/N_2_ elimination/cyclization pathway. The first step is a nucleophilic attack identical to the Diels–Alder mechanism, where the formed zwitterionic intermediate **IM13** was found to be in equilibrium with three other tautomers (**IM16**–**18**, Figure 6B). While all these isomers can undergo the retro Diels–Alder reaction with low barriers, the activation energy for isomer **IM17** is the lowest at 11.2 kcal/mol in relation to the starting material, forming the highly stable intermediate **IM19**. **IM13**, **IM16**, and **IM18** can also undergo dinitrogen elimination to yield isomers of **IM19** (see Figure S13). Similar to the case of **IM7**, intermediate **IM19** is also in equilibrium with a collection of isomers, including electrocyclization precursors **IM20** and **IM21** (Figure 6C), which are also interconvertible through intramolecular proton transfer (**TS14**, corrected for tunneling, see Figure S14). Although intermediate **IM20** is considerably less stable than **IM21**, the corresponding transition state (**TS15**) of the electrocyclization is −6.6 kcal/mol lower in energy than the alternative **TS16**. Kinetic control leads to the cyclized product **IM15**, which gives the final product **7c** after the loss of NH_3_. Such a reaction pathway is predicted to provide exclusive formation of the pyrimidine product containing only one of the nitrogen atoms originating from the amidine. This agrees with our labeling studies where only singly ^15^N-labeled **7c** is observed when triazine **3** is treated with doubly ^15^N-labeled amidine **4c**. This observation is not limited to this specific case but is a general result where singly ^15^N-labeled products are generated exclusively when 1,2,3-triazines **2** and **3** are treated with various ^15^N-labeled amidines (Figure 2). Similar to the case of 1,2,3,5-tetrazine **1**, the initial nucleophilic attack was identified as the rate-limiting step, which agrees with the observed Hammett relationship for the electronically and sterically very similar triazine **2** (Figure 3B). Although both of the reaction pathways are predicted to provide exclusive singly ^15^N-labeled pyrimidine products, the addition/N_2_ elimination/cyclization mechanism is supported by the nature of the rate-limiting step and the energetically favored N_2_ elimination.

**Figure 7:**
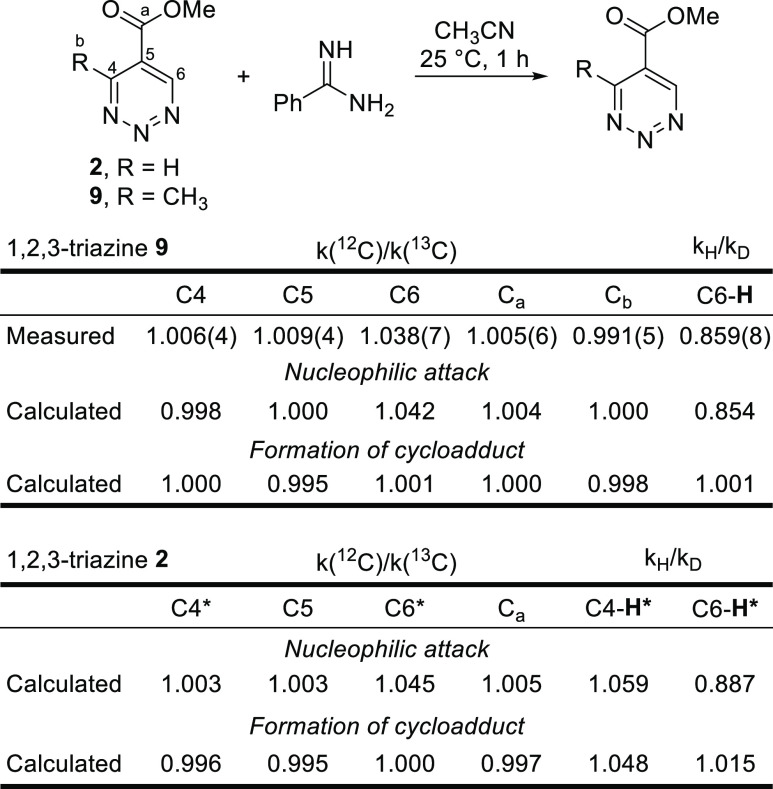
KIE studies. *For calculated KIEs, a nucleophilic attack in position 6 was used.

Additional studies of the kinetic isotope effect (KIE) of key atoms in the azadienes provided further insights into the reaction mechanism (Figure 7). A combined experimental and theoretical investigation of the KIE was conducted on 1,2,3-triazines, where both ^12^C/^13^C and additional H/D KIE information due to the presence of the aromatic protons on the azadiene system could be examined. Due to the symmetrical nature of 1,2,3-triazine **2**, experimental measurements of H/D and ^12^C/^13^C KIE were conducted on 1,2,3-triazine **9**, a previously reported^32^ reactive triazine with an added C4 methyl substituent, to allow their precise determination. The ^12^C/^13^C KIE of all carbons in 1,2,3-triazine **9** was determined through Singleton’s method,^36^ and the direct intermolecular competition method was applied for the H/D KIE determination of the triazine aromatic hydrogen. A significant primary ^12^C/^13^C KIE (1.038) of the 1,2,3-triazine C6 carbon and a strong inverse secondary H/D KIE (0.859) of the aromatic proton in triazine **9** were observed, while the ^12^C/^13^C KIE of the remaining carbons including C4 were found to be essentially 1 (1.00). The observed results are indicative of a direct bond formation of only the C6 carbon and a change in its hybridization state from sp^2^ to sp^3^ in the transition state of the rate-determining step.

These results agree well with the calculated KIE for an initial nucleophilic attack at C6 (C6 ^12^C/^13^C KIE = 1.042, H/D KIE = 0.854), consistent with the addition/N_2_ elimination/cyclization mechanism with the nucleophilic addition being the first and rate-determining step. The reaction pathway through a rate-determining formation of the cycloadduct features a calculated KIE of 1.001 for the C6 carbon, 1.000 for the C4 carbon, and 1.001 for the aromatic proton on triazine **9**, which is distinct from the observed results and further indicating that it is an unlikely mechanism. For comparison, energy profiles (Supporting Information Figure S15) and theoretical KIEs were also calculated for 1,2,3-triazine **2** and resemble the results obtained with methyl 1,2,3-triazine **9**.

## Conclusions

Through combined experimental and computational mechanistic studies, the suspected concerted or stepwise Diels–Alder reaction pathway was ruled out as a plausible reaction mechanism for the reaction of 1,2,3-triazines/1,2,3,5-tetrazines with amidines. Instead, the results revealed that a stepwise addition/N_2_ elimination/cyclization pathway is a more likely mechanism. An initial nucleophilic attack of the electron-rich amidine nitrogen on the electron-deficient 1,2,3-triazine/1,2,3,5-tetrazine carbon was revealed to be the rate-determining step, which is in agreement with the calculated highest energy barrier, absence of detectable reaction intermediates, a singularly strong KIE on C6 carbon (of **9**) as well as the attached proton, and the strong Hammett relationship of the para-substituents of the arylamidines on the overall reactivity with a positive charge accumulation on the amidine in the transition state.

Subsequent reaction progresses through a highly exothermic retro Diels–Alder reaction with the elimination of N_2_, avoiding the formation of an intermediate and unstable bicyclic Diels–Alder adduct. Incorporation of one or two amidine nitrogen atoms, as observed by ^15^N-labeling studies, is kinetically controlled at the 6π electrocyclization step. Electronic properties of the substituents of the triene intermediate as well as its intrinsic reactivity were found to determine the ratio of singly versus doubly labeled products. The proposed reaction mechanism provided deeper insights into this formal inverse electron demand Diels–Alder reaction as well as further understanding of the origin of the observed orthogonal reactivity between polarized heterocyclic systems (including 1,2,3,5-tetrazine and 1,2,3-triazine) and relatively non-polarized heterocyclic azadienes (including 1,2,4,5-tetrazine and 1,2,4-triazine).

## Supporting Information Available

Computational methods and results, additional computational data, organic synthesis, reaction kinetics, labeling studies, kinetic isotope effect studies, and copies of NMR spectra (PDF)
Coordinates of all calculated geometries (ZIP)
