# Supplementary material for: Mechanistic Insights into the Reaction of Amidines with 1,2,3-Triazines and 1,2,3,5-Tetrazines
Source: J Am Chem Soc. 2022 Jun 6;144(24):10921–8. doi: 10.1021/jacs.2c03726 (PMC9228069; doi:10.1021/jacs.2c03726)
Supplement: Supplementary file 1 — ja2c03726_si_001.pdf [file ja2c03726_si_001.pdf]

Supporting Information

Mechanistic Insights into the Reaction of Amidines With 1,2,3-Triazines and 1,2,3,5-Tetrazines

Zhi-Chen Wu,[§] K. N. Houk,[‡] Dale L. Boger,[§,||,*] and Dennis Svatunek,[†,‡,*]

[§] Department of Chemistry, The Scripps Research Institute, La Jolla, California, 92037, United States

[‡] Department of Chemistry and Biochemistry, University of California, Los Angeles, Los Angeles, California, 90095, United States

[||] Department of Chemistry, The Skaggs Institute for Chemical Biology, La Jolla, California, 92037, United States

[†] Institute of Applied Synthetic Chemistry, TU Wien, 1060 Vienna, Austria

Table of Contents

I. General Methods 2

II. ^{15}N labeling experiments 2

III. Kinetic studies 16

IV. Kinetic Isotope Effect Studies 27

IV. Computational Studies 32

V. References 40

VI. Spectra 41

I. General Methods

General Methods. All reagents and solvents were used as supplied without further purification unless otherwise noted. Preparative TLC (PTLC) and column chromatography were conducted using Millipore SiO_2 60 F_{254} PTLC (0.5 mm) and Zeochem ZEOprep 60 ECO SiO_2 (40–63 µm), respectively. Analytical TLC was conducting using Millipore SiO_2 60 F254 TLC (0.250 mm) plates. ^{1}H and ^{13}C NMR spectra were obtained using a Bruker Avance III HD 600 MHz spectrometer equipped with either a 5 mm QCI or 5 mm CPDCH probe. Mass spectrometry analysis was performed by direct sample injection on an Agilent G1969A ESI-TOF mass spectrometer.

II. ^{15}N labeling experiments

Preparation of doubly ^{15}N-labeled amidines 4a-e

4a was prepared according to a literature procedure.[1]

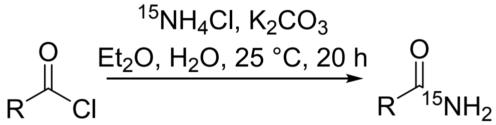

A solution of the corresponding acyl chloride (3 mmol, 1 equiv) in Et_2O (6 mL) was treated with a solution of K_2CO_3 (621 mg, 4.5 mmol, 1.5 equiv) and $^{15}NH_4Cl$ (246 mg, 4.5 mmol, 1.5 equiv) in H_2O (6 mL). The mixture was stirred at 25 °C for 20 h before 2M KOH (20 mL) was added. The mixture was extracted by EtOAc (40 mL × 3). The organic layers were combined, dried over Na_2SO_4, then concentrated to provide corresponding ^{15}N-labeled amides as white solids: R = cyclohexyl, reaction scaled up by 2-fold, 414 mg, 54%; R = *p*-$MeOC_6H_4$, 252 mg, 44%; R = *p*-$CF_3C_6H_4$, 547 mg, 84%.

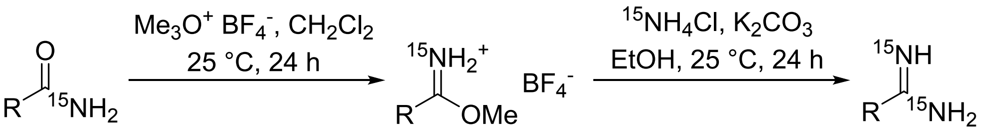

In a flame-dried reaction vessel, corresponding ^{15}N-labeled amide (1 mmol, 1 equiv) was added to a suspension of trimethyloxonium tetrafluoroborate (222 mg, 1.5 mmol, 1.5 equiv) in anhydrous CH_2Cl_2 (1 mL). The mixture was stirred under argon atmosphere at 25 °C for 24 h. Solvent was

then removed to provide crude imidate salt. This crude was dissolved in EtOH (6 mL) and the mixture was treated with K_2CO_3 (414 mg, 3 mmol, 3 equiv) and $^{15}NH_4Cl$ (163.5 mg, 3 mmol, 3 equiv). The mixture was stirred at 25 °C for 24 h and was then concentrated. 1M HCl (40 mL) was added to the residue and the aqueous phase was washed with CH_2Cl_2 (50 mL × 2). The aqueous phase was then basified by 2M KOH (50 mL) and extracted with CH_2Cl_2 (50 mL × 3). The organic layers were combined, dried over Na_2SO_4, then concentrated to provide corresponding doubly ^{15}N-labeled amidines:

[^{15}N]$_2$-Cyclohexylformamidine (4b)

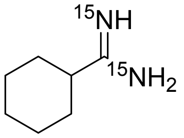

4b (70 mg, 55% over two steps, viscous oil): ^1H NMR (500 MHz, $CDCl_3$, 298K) δ 2.07–2.03 (m, 1H), 1.93–1.89 (m, 2H), 1.84–1.80 (m, 2H), 1.72–1.69 (m, 1H), 1.32–1.27 (m, 4H), 1.23–1.18 (m, 1H); ^{13}C NMR (125 MHz, $CDCl_3$, 298K) δ 172.4 (t, J = 11 Hz), 44.8, 30.9, 29.7, 26.0; HRMS ESI-TOF *m/z* 129.1172 ([M + H]$^+$, $C_7H_{14}[^{15}N]_2 + H^+$ requires 129.1170).

[^{15}N]$_2$-Benzamidine (4c)

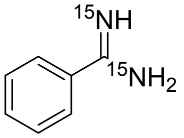

4c (73 mg, 60% over two steps, viscous oil): ^1H NMR (500 MHz, $CDCl_3$, 298K) δ 7.60 (dd, 2H, J = 8.0, 1.5 Hz), 7.46 (t, 1H, J = 7.0 Hz), 7.42 (t, 2H, J = 7.0 Hz); ^{13}C NMR (125 MHz, $CDCl_3$, 298K) δ 166.0 (t, J = 10 Hz), 136.4, 130.7, 128.9, 126.1; HRMS ESI-TOF *m/z* 123.0702 ([M + H]$^+$, $C_7H_8[^{15}N]_2 + H^+$ requires 123.0701).

[^{15}N]$_2$-4-Methoxybenzamidine (4d)

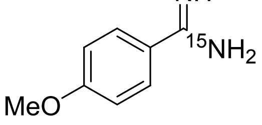

4d (108 mg, 71% over two steps, white solid): ^{1}H NMR (600 MHz, $CDCl_3$, 298K) δ 7.57 (d, 2H, *J* = 8.6 Hz), 6.92 (d, 2H, *J* = 8.6 Hz), 3.84 (s, 3H); ^{13}C NMR (151 MHz, $CDCl_3$, 298K) δ 165.4 (t, *J* = 9 Hz), 161.5, 128.8, 127.7, 114.1, 55.5; HRMS ESI-TOF *m/z* 153.0808 ($[M + H]^+$, $C_8H_{10}O[^{15}N]_2 + H^+$ requires 153.0807).

$[^{15}N]_2$-4-Trifluoromethylbenzamidine (4e)

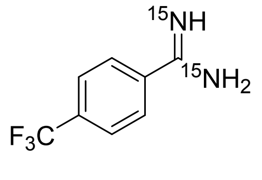

4e (36 mg, 20% over two steps, white solid): ^{1}H NMR (600 MHz, $CDCl_3$, 298K) δ 7.74 (d, 2H, *J* = 8.0 Hz), 7.69 (d, 2H, *J* = 8.0 Hz); ^{13}C NMR (151 MHz, $CDCl_3$, 298K) δ 163.7 (t, *J* = 16 Hz), 139.3, 131.9 (q, *J* = 33 Hz), 126.1, 125.3, 123.2 (q, *J* = 272 Hz); HRMS ESI-TOF *m/z* 191.0585 ($[M + H]^+$, $C_8H_7F_3[^{15}N]_2 + H^+$ requires 191.0575).

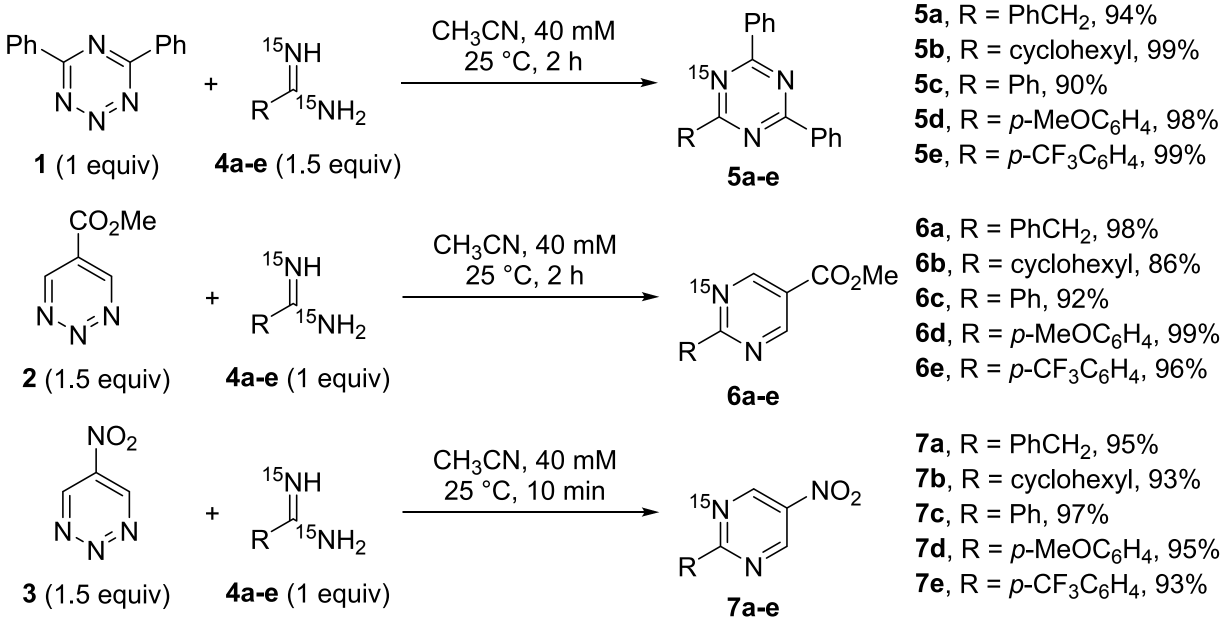

Figure S1. Reactions between 1,2,3,5-tetrazine (**1**) or 1,2,3-triazines (**2** and **3**) with labeled amidines (**4a**-**e**)

Reactions between 4,6-diphenyl-1,2,3,5-tetrazine (1) and labeled amidines (4a-e)

A 40 mM solution of **1** (4.68 mg, 0.02 mmol, 1 equiv) in anhydrous CH_3CN (0.5 mL) was treated with the corresponding double ^{15}N-labelled amidine **4a-e** (0.03 mmol, 1.5 equiv). The mixture was stirred at 25 °C for 2 h, and the solvent was removed under a gentle stream of N_2. The residue was purified by column chromatography (SiO_2, EtOAc/hexanes) or PTLC (EtOAc/hexanes) to provide **5a-e** as desired products.

[^{15}N]-2-Benzyl-4,6-diphenyl-1,3,5-triazine and [^{15}N]$_2$-2-Benzyl-4,6-diphenyl-1,3,5-triazine (5a)

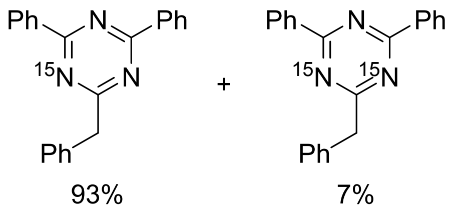

Detailed in ref. S2.[2]

[^{15}N]-2-Cyclohexyl-4,6-diphenyl-1,3,5-triazine and [^{15}N]$_2$-2-Cyclohexyl-4,6-diphenyl-1,3,5-triazine (5b)

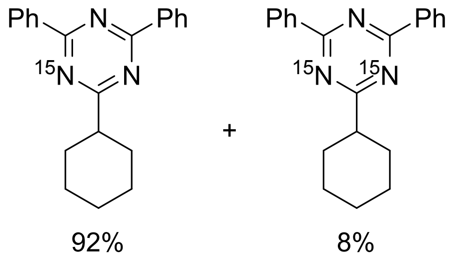

5b (6.24 mg, 99%, white solid): ^{1}H NMR (600 MHz, $CDCl_3$, 298K) δ 8.67 (dd, 4H, J = 8.3 Hz, 1.4 Hz), 7.58 (t, 2H, J = 7.2 Hz), 7.54 (t, 4H, J = 7.2 Hz), 2.97–2.91 (m, 1H), 2.17–2.12 (m, 2H), 1.94–1.89 (m, 2H), 1.84–1.76 (m, 3H), 1.49 (qt, 2H, J = 12.9 Hz, 3.3 Hz), 1.38 (qt, 1H, J = 13.4 Hz, 3.3 Hz); ^{13}C NMR (151 MHz, $CDCl_3$, 298K) δ 183.1 (d, J = 2 Hz), 171.3 (m), 136.5 (t, J = 4 Hz), 132.4, 129.0, 128.7, 47.3 (d, J = 8 Hz), 31.4, 26.21, 26.19; HRMS ESI-TOF m/z 317.1782 ($[M + H]^+$, $C_{21}H_{21}N_2[^{15}N] + H^+$ requires 317.1779).

[^{15}N]-2,4,6-triphenyl-1,3,5-triazine and [^{15}N]$_2$-2,4,6-triphenyl-1,3,5-triazine (5c)

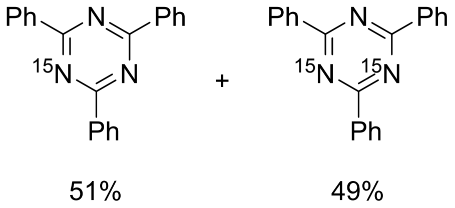

5c (5.50 mg, 90%, white solid): ^{1}H NMR (600 MHz, $CDCl_3$, 298K) δ 8.79 (d, 6H, *J* = 7.4 Hz), 7.63 (t, 3H, *J* = 7.1 Hz), 7.59 (t, 6H, *J* = 7.3 Hz); ^{13}C NMR (151 MHz, $CDCl_3$, 298K) δ 171.8 (m), 136.4 (m), 132.8, 129.3 (m), 128.8; HRMS ESI-TOF *m/z* 311.1309, 312.1297 ([M + H]$^+$, $C_{21}H_{15}N_2[^{15}N] + H^+$ requires 311.1312, $C_{21}H_{15}N[^{15}N]_2 + H^+$ requires 312.1279).

[^{15}N]-2-(4-Methoxyphenyl)-4,6-diphenyl-1,3,5-triazine and [^{15}N]$_2$-2-(4-Methoxyphenyl)-4,6-diphenyl-1,3,5-triazine (5d)

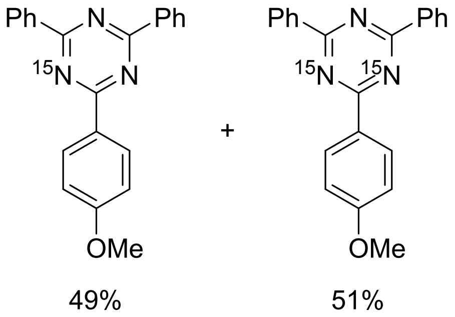

5d (6.68 mg, 98%, white solid): ^{1}H NMR (600 MHz, $CDCl_3$, 298K) δ 8.77 (dd, 4H, *J* = 8.2, 1.4 Hz), 8.75 (d, 2H, *J* = 8.9 Hz), 7.61 (t, 2H, *J* = 7.2 Hz), 7.57 (t, 4H, *J* = 7.2 Hz), 7.07 (d, 2H, *J* = 8.9 Hz), 3.92 (s, 3H); ^{13}C NMR (151 MHz, $CDCl_3$, 298K) δ 171.5 (m), 171.3 (m), 163.5, 136.6 (m), 132.5, 131.0 (m), 129.0 (m), 128.9 (m), 128.7, 114.1, 55.6; HRMS ESI-TOF *m/z* 341.1421, 342.1403 ([M + H]$^+$, $C_{22}H_{17}ON_2[^{15}N] + H^+$ requires 341.1415, $C_{22}H_{17}ON[^{15}N]_2 + H^+$ requires 342.1385).

[^{15}N]-2-(4-Trifluoromethylphenyl)-4,6-diphenyl-1,3,5-triazine and [^{15}N]$_2$-2-(4-Trifluoromethylphenyl)-4,6-diphenyl-1,3,5-triazine (5e)

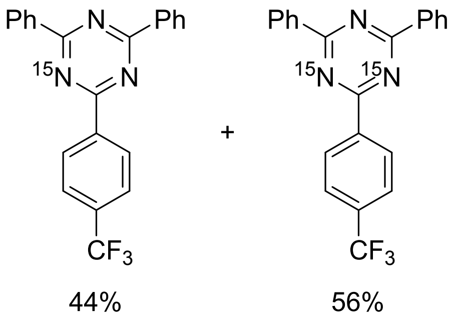

5e (7.55 mg, 99%, white solid): ^{1}H NMR (600 MHz, $CDCl_3$, 298K) δ 8.88 (d, 2H, J = 8.2 Hz), 8.77 (d, 4H, J = 7.1 Hz), 7.83 (d, 2H, J = 8.2 Hz), 7.64 (t, 2H, J = 7.2 Hz), 7.59 (t, 4H, J = 7.2 Hz); ^{13}C NMR (151 MHz, $CDCl_3$, 298K) δ 172.1 (m), 170.6 (m), 139.7 (m), 136.0 (m), 134.0 (q, J = 32 Hz), 132.9, 129.4 (m), 129.2 (m), 128.9, 125.7 (q, J = 4 Hz), 124.2 (q, J = 273 Hz); HRMS ESI-TOF *m/z* 379.1186, 380.1165 ($[M + H]^+$, $C_{22}H_{14}F_3N_2[^{15}N] + H^+$ requires 379.1183, $C_{22}H_{14}F_3N[^{15}N]_2 + H^+$ requires 380.1153).

Reactions between 5-methoxycarbonyl-1,2,3-triazine (2) and labelled amidines (4a-e)

A 60 mM solution of **2** (8.4 mg, 0.06 mmol, 1.5 equiv) in anhydrous CH_3CN (1 mL) was treated with the corresponding double ^{15}N-labeled amidine **2a-e** (0.04 mmol, 1 equiv). The mixture was stirred at 25 °C for 2 h, and the solvent was removed under a gentle stream of N_2. The residue was purified by column chromatography (SiO_2, EtOAc/hexanes) or PTLC (EtOAc/hexanes) to provide **6a-e** as desired products.

[^{15}N]-Methyl 2-benzylpyridine-5-carboxylate (6a)

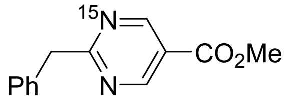

Detailed in ref. S3.[3]

[^{15}N]-Methyl 2-cyclohexylpyridine-5-carboxylate (6b)

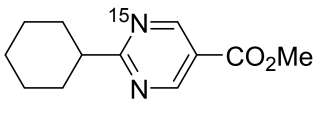

6b (7.62 mg, 86%, white solid): ^{1}H NMR (600 MHz, $CDCl_3$, 298K) δ 9.20–9.17 (m, 2H), 3.96 (s, 3H), 2.96 (tq, 1H, J = 11.8 Hz, 2.4 Hz), 2.01 (d, 2H, J = 13.0 Hz), 1.86 (dt, 2H, J = 13.2 Hz, 3.2 Hz), 1.75 (d, 1H, J = 12.8 Hz), 1.64 (qd, 2H, J = 12.4 Hz, 3.4 Hz), 1.42 (qt, 2H, J = 13.0 Hz, 3.3 Hz), 1.31 (qt, 1H, J = 12.8 Hz, 3.5 Hz); ^{13}C NMR (151 MHz, $CDCl_3$, 298K) δ 178.3 (d, J = 3 Hz), 164.7, 158.3 (m), 121.4 (d, J = 2 Hz), 52.7, 47.9 (d, J = 8 Hz), 31.9, 26.3, 26.0; HRMS ESI-TOF *m/z* 222.1263 ($[M + H]^+$, $C_{12}H_{16}O_2N[^{15}N] + H^+$ requires 222.1255).

[^{15}N]-Methyl 2-phenylpyridine-5-carboxylate (6c)

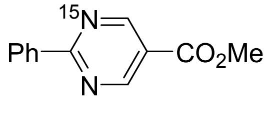

6c (7.85 mg, 92%, white solid): ^{1}H NMR (600 MHz, $CDCl_3$, 298K) δ 9.34–9.31 (m, 2H), 8.53 (d, 2H, J = 6.3 Hz), 7.57–7.48 (m, 3H), 4.00 (s, 3H); ^{13}C NMR (151 MHz, $CDCl_3$, 298K) δ 167.4 (d, J = 2 Hz), 164.6, 158.6 (m), 136.7 (d, J = 9 Hz), 132.0, 129.1 (d, J = 2 Hz), 128.9, 121.6, 52.7; HRMS ESI-TOF *m/z* 216.0789 ($[M + H]^+$, $C_{12}H_{10}O_2N[^{15}N] + H^+$ requires 216.0786).

[^{15}N]-Methyl 2-(4-methoxyphenyl)pyridine-5-carboxylate (6d)

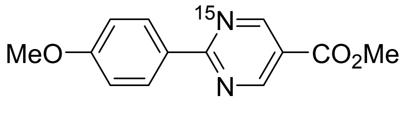

6d (9.71 mg, 99%, white solid): ^{1}H NMR (600 MHz, $CDCl_3$, 298K) δ 9.29–9.25 (m, 2H), 8.49 (d, 2H, J = 8.0 Hz), 7.01 (d, 2H, J = 8.0 Hz), 3.98 (s, 3H), 3.89 (s, 3H); ^{13}C NMR (151 MHz, $CDCl_3$, 298K) δ 167.1 (d, J = 2 Hz), 164.8, 163.0, 158.5 (m), 131.0 (d, J = 2 Hz), 129.4 (d, J = 9 Hz), 120.8 (d, J = 2 Hz), 114.3, 55.6, 52.6; HRMS ESI-TOF *m/z* 246.0894 ($[M + H]^+$, $C_{13}H_{12}O_3N[^{15}N] + H^+$ requires 246.0891).

[^{15}N]-Methyl 2-(4-trifluoromethylphenyl)pyridine-5-carboxylate (6e)

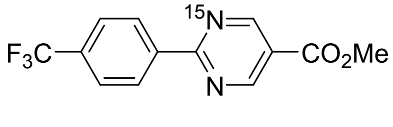

6e (10.81 mg, 96%, white solid): ^{1}H NMR (600 MHz, $CDCl_3$, 298K) δ 9.37–9.33 (m, 2H), 8.66 (d, 2H, J = 7.8 Hz), 7.78 (d, 2H, J = 7.8 Hz), 4.01(s, 3H); ^{13}C NMR (151 MHz, $CDCl_3$, 298K) δ 166.0, 164.3, 158.5 (m), 139.9 (d, J = 9 Hz), 133.4 (q, J = 32 Hz), 129.4 (d, J = 2 Hz), 125.8 (q, J

= 4 Hz), 124.1 (q, J = 272 Hz), 122.4 (d, J = 2 Hz), 52.9; HRMS ESI-TOF *m/z* 284.0662 ([M + H]$^+$, $C_{13}H_9F_3O_2N[^{15}N]$ + H^+ requires 284.0659).

Reactions between 5-nitro-1,2,3-triazine (3) and labelled amidines (4a-e)

A 60 mM solution of **3** (7.5 mg, 0.06 mmol, 1.5 equiv) in anhydrous CH_3CN (1 mL) was treated with the corresponding double ^{15}N-labeled amidine **4a-e** (0.04 mmol, 1 equiv). The mixture was stirred at 25 °C for 10 min, and the solvent was removed under a gentle stream of N_2. The residue was purified by column chromatography (SiO_2, CH_2Cl_2) to provide **7a-e** as desired products.

[^{15}N]-2-Benzyl-5-nitropyridine (7a)

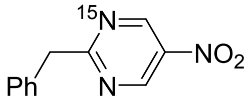

7a (8.19 mg, 95%, white solid): ^{1}H NMR (600 MHz, $CDCl_3$, 298K) δ 9.42–9.40 (m, 2H), 7.36 (d, 2H, J = 7.0 Hz), 7.33 (t, 2H, J = 7.6 Hz), 7.26 (t, 1H, J = 7.6 Hz), 4.43 (d, 2H, J = 2.6 Hz); ^{13}C NMR (151 MHz, $CDCl_3$, 298K) δ 175.1 (d, J = 4 Hz), 152.9 (m), 140.8, 136.7, 129.4, 129.0, 127.4, 46.2 (d, J = 9 Hz); HRMS ESI-TOF *m/z* 217.0739 ([M + H]$^+$, $C_{11}H_9O_2N_2[^{15}N]$ + H^+ requires 217.0738).

[^{15}N]-2-Cyclohexyl-5-nitropyridine (7b)

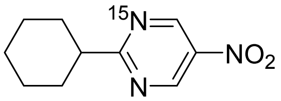

7b (7.76 mg, 93%, white solid): ^{1}H NMR (600 MHz, $CDCl_3$, 298K) δ 9.42–9.39 (m, 2H), 3.06 (tq, 1H, J = 11.8 Hz, 3.0 Hz), 2.06–2.01 (m, 2H), 1.91–1.87 (m, 2H), 1.80–1.76 (m, 1H), 1.66 (qd, 2H, J = 12.5 Hz, 3.4 Hz), 1.44 (qt, 2H, J = 13.0 Hz, 3.5 Hz), 1.32 (qt, 1H, J = 12.8 Hz, 3.6 Hz); ^{13}C NMR (151 MHz, $CDCl_3$, 298K) δ 180.1 (d, J = 4 Hz), 152.5 (m), 140.6 (d, J = 2 Hz), 47.9 (d, J = 8 Hz), 31.9, 26.1, 25.9; HRMS ESI-TOF *m/z* 209.1049 ([M + H]$^+$, $C_{10}H_{13}O_2N_2[^{15}N]$ + H^+ requires 209.1051).

[^{15}N]-5-Nitro-2-phenyl-pyridine (7c)

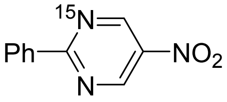

7c (7.80 mg, 97%, white solid): ^{1}H NMR (600 MHz, $CDCl_3$, 298K) δ 9.54–9.51 (m, 2H), 8.53 (dd, 2H, J = 8.4 Hz, 1.2 Hz), 7.60 (t, 2H, J = 7.3 Hz), 7.55 (t, 1H, J = 7.3 Hz); ^{13}C NMR (151 MHz, $CDCl_3$, 298K) δ 168.4 (d, J = 2 Hz), 153.0 (m), 140.7, 135.7 (d, J = 9 Hz), 133.1, 129.8 (d, J = 3 Hz), 129.1; HRMS ESI-TOF *m/z* 203.0580 ([M + H]$^+$, $C_{10}H_7O_2N_2[^{15}N] + H^+$ requires 203.0581).

[^{15}N]-2-(4-methoxyphenyl)-5-nitro-pyridine (7d)

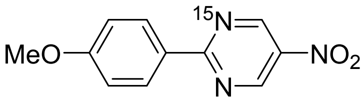

7d (8.86 mg, 95%, yellow solid): ^{1}H NMR (600 MHz, $CDCl_3$, 298K) δ 9.46–9.44 (m, 2H), 8.52 (d, 2H, J = 8.2 Hz), 7.03 (d, 2H, J = 8.2 Hz), 3.91 (s, 3H); ^{13}C NMR (151 MHz, $CDCl_3$, 298K) δ 168.0 (d, J = 2 Hz), 163.9, 153.0 (m), 140.0, 131.9 (d, J = 3 Hz), 128.3 (d, J = 9 Hz), 114.6, 55.7; HRMS ESI-TOF *m/z* 233.0696 ([M + H]$^+$, $C_{11}H_9O_3N_2[^{15}N] + H^+$ requires 233.0687).

[^{15}N]-5-Nitro-2-(4-trifluoromethylphenyl)-pyridine (7e)

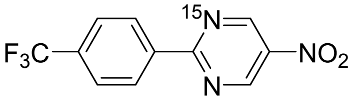

7e (10.03 mg, 93%, white solid): ^{1}H NMR (600 MHz, $CDCl_3$, 298K) δ 9.58–9.56 (m, 2H), 8.69 (d, 2H, J = 9.0 Hz), 7.81 (d, 2H, J = 9.0 Hz); ^{13}C NMR (151 MHz, $CDCl_3$, 298K) δ 167.0 (d, J = 2 Hz), 153.2 (m), 141.1, 138.7 (d, J = 10 Hz), 134.3 (q, J = 32 Hz), 130.1 (d, J = 3 Hz), 126.0 (q, J = 4 Hz), 123.9 (q, J = 273 Hz); HRMS ESI-TOF *m/z* 271.0455 ([M + H]$^+$, $C_{11}H_6F_3O_2N_2[^{15}N]$ + H^+ requires 271.0455).

Determination of product ratio by HRMS

Products of the isotope labeling experiments were subjected to HRMS analysis, where the ratio of signal intensity (peak area) of M + H^+ (single ^{15}N-labeled product) and M + 1 + H^+ (double ^{15}N-labeled product) was used to determine the ^{15}N content in the reaction product with the existence of natural occurring isotopes taken into account following the equation below. Two parallel experiments are conducted to provide the average, summarized in Table S1. Data for products **5a** and **6a** were included in our previous reports.[2,3]

$$\text{Second } ^{15}\text{N } (\%) = \frac{Intensity_{M+1+H^+} - Intensity_{M+H^+} \times Natural\ Abundance_{M+1+H^+}}{Intensity_{M+H^+} + Intensity_{M+1+H^+} - Intensity_{M+H^+} \times Natural\ Abundance_{M+1+H^+}}$$

Table S1. Determination of second incorporation ratio in products **5-7** by HRMS

Product	Natural isotope abundance (M + 1 + H^+)	Intensity (M + H^+)	Intensity (M + 1 + H^+)	Second ^{15}N incorporation
**5b**	23.9%	23861	89161	7.8%
		30194	112419	7.8%
**5c**	23.8%	7111	8592	49.2%
		4844	5784	48.9%
**5d**	24.9%	70523	89454	50.5%
		73127	93429	50.7%
**5e**	24.8%	1448	2245	56.6%
		1117	1681	55.7%
**6b**	13.8%	64006	8249	< 0%
		51947	6546	< 0%
**6c**	13.7%	15869	1975	< 0%
		39190	4730	< 0%
**6d**	14.8%	39011	5043	< 0%
		36330	4515	< 0%
**6e**	14.7%	7460	1098	< 0%
		9267	1290	< 0%
**7a**	12.8%	19840	2779	1.2%
		12654	1791	1.3%
**7b**	12.9%	75492	10647	3.1%
		85246	12109	2.8%
**7c**	11.9%	23947	3617	0.8%
		17232	2550	0.4%
**7d**	11.8%	49238	6189	0.7%
		58995	7225	0.2%
**7e**	12.9%	4363	595	1.2%
		2323	305	1.3%

Determination of product ratio of 5a-e by ^{13}C NMR

Data for product **5a** was included in our previous report.[2]

[^{15}N]-2-cyclohexyl-4,6-diphenyl-1,3,5-triazine (5b)

The analysis focused on the signal of the substituted carbon (a) on the phenyl ring:

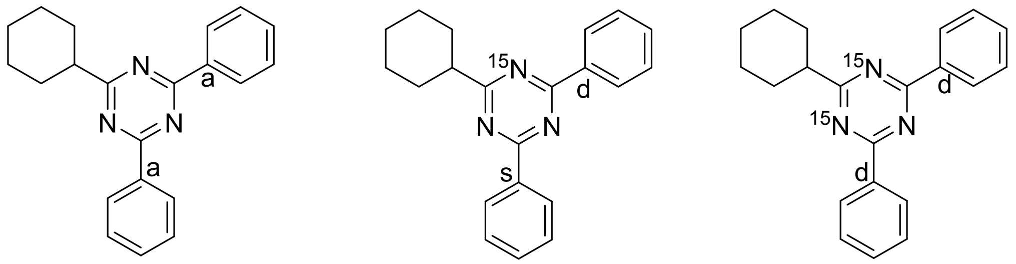

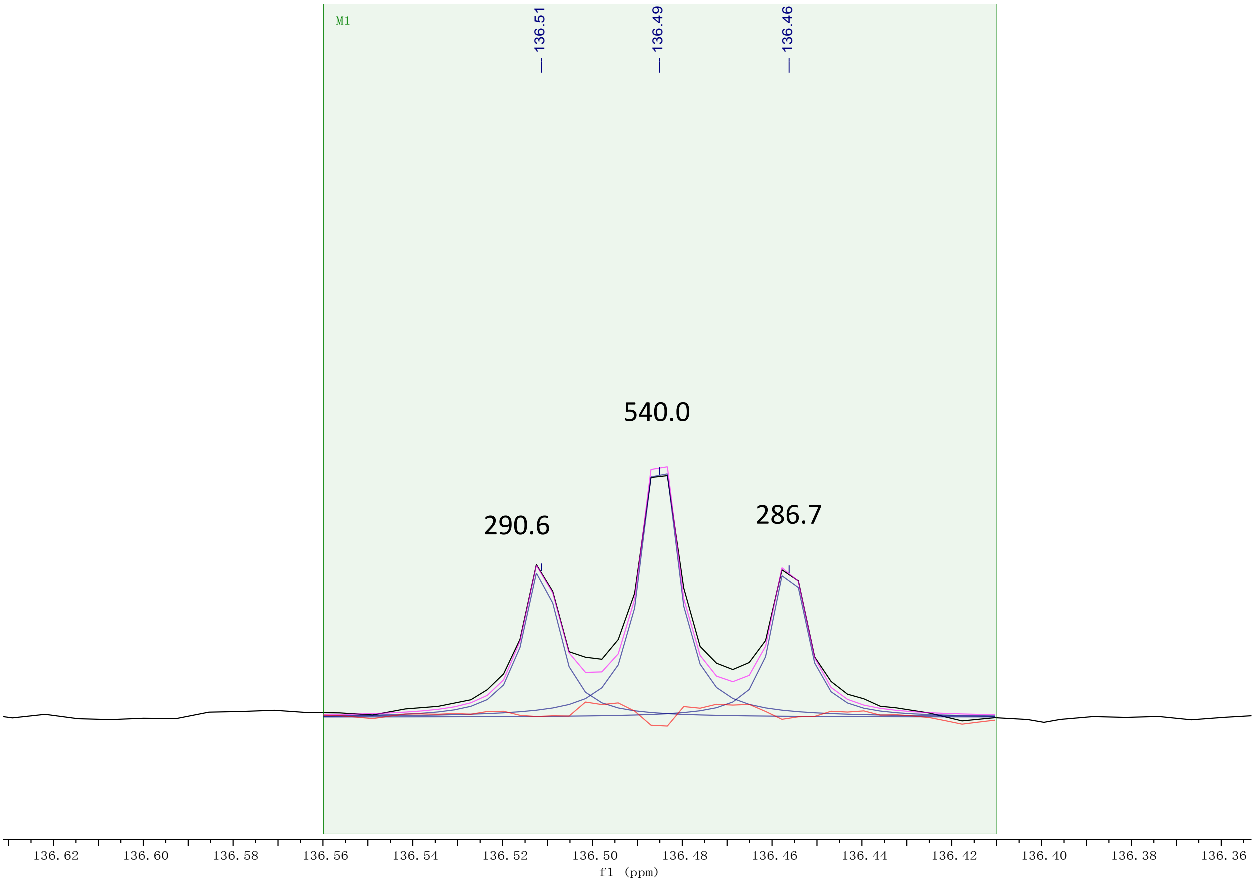

$$percentage\ of\ double\ ^{15}N\ labeled\ product = \frac{290.6 + 286.7 - 540.0}{290.6 + 286.7 + 540.0} = 3\%$$

[^{15}N]-2,4,6-triphenyl-1,3,5-triazine (5c)

The analysis focused on the signal of the substituted carbon (a) on the phenyl ring:

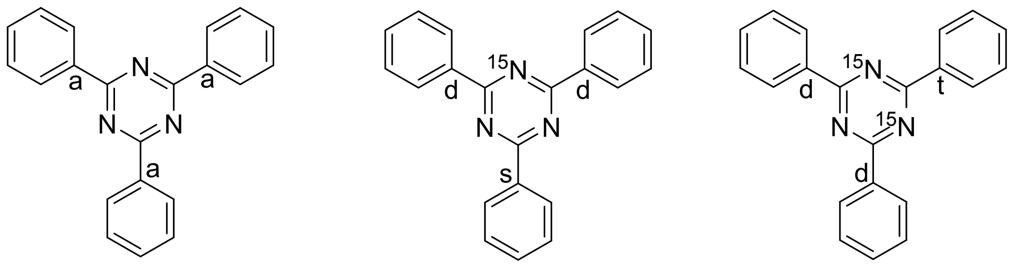

Single ^{15}N labeled product would give a doublet and a singlet, resulting in three peaks (ratio 1 : 1 : 1), double ^{15}N labeled product would give a doublet and a triplet, resulting in five peaks (ratio 1 : 4 : 2 : 4 : 1). The spectra observed reveals a mixture of single ^{15}N and double ^{15}N labelled product.

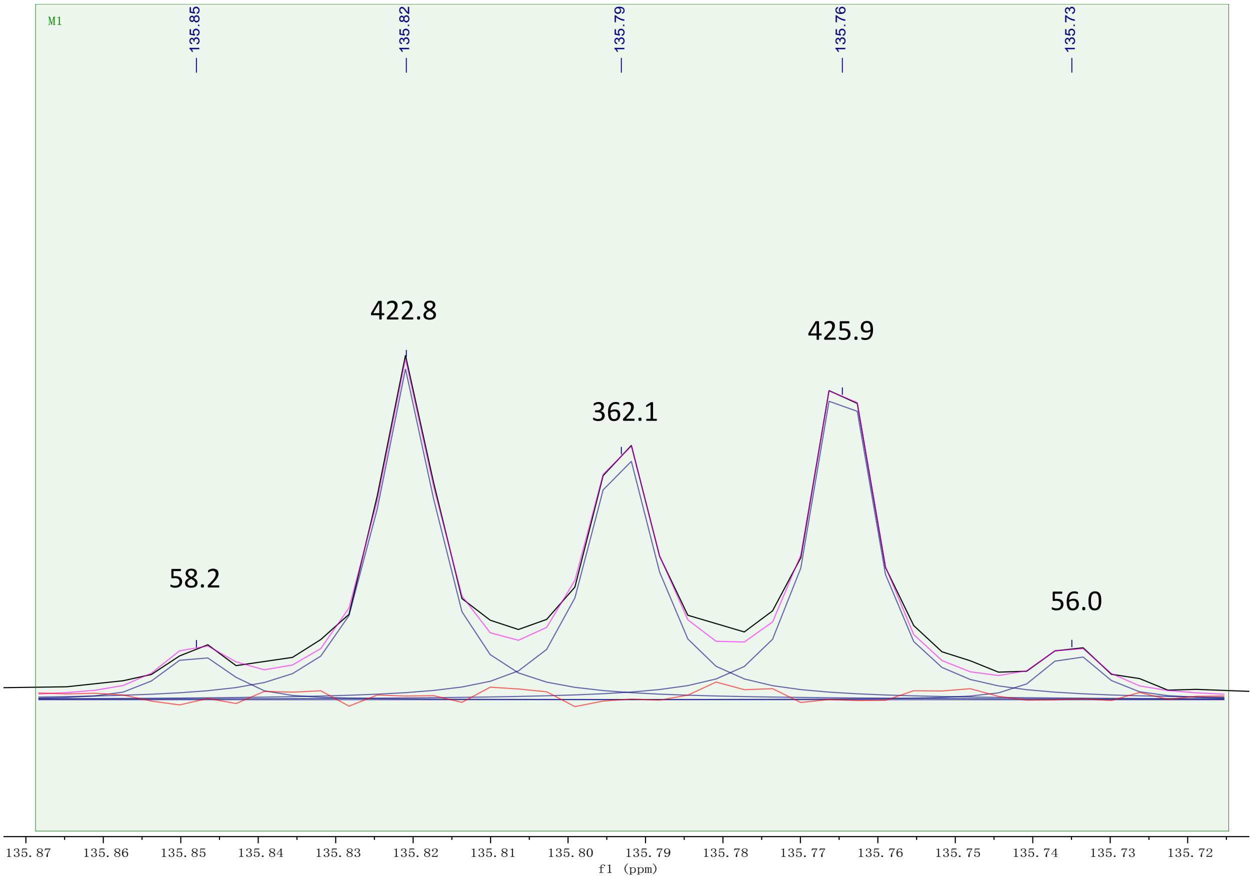

$$percentage\ of\ double\ ^{15}N\ labeled\ product = \frac{(58.2 + 56.0) \times 2}{362.1 + 58.2 + 56.0} = 48\%$$

[^{15}N]-2-(4-methoxyphenyl)-4,6-diphenyl-1,3,5-triazine (5d)

The analysis focused on the signal of the substituted carbon (a) on the phenyl ring:

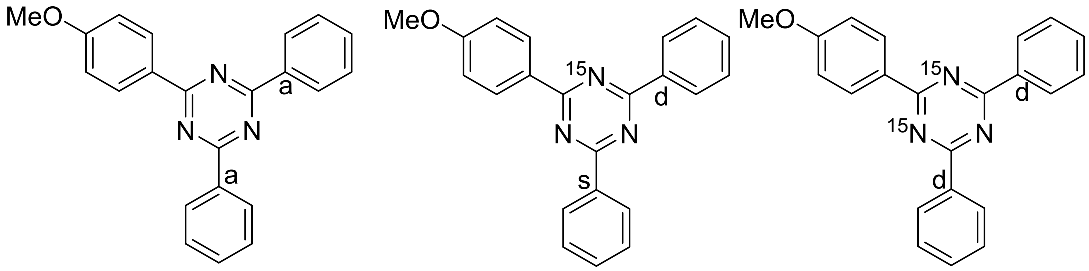

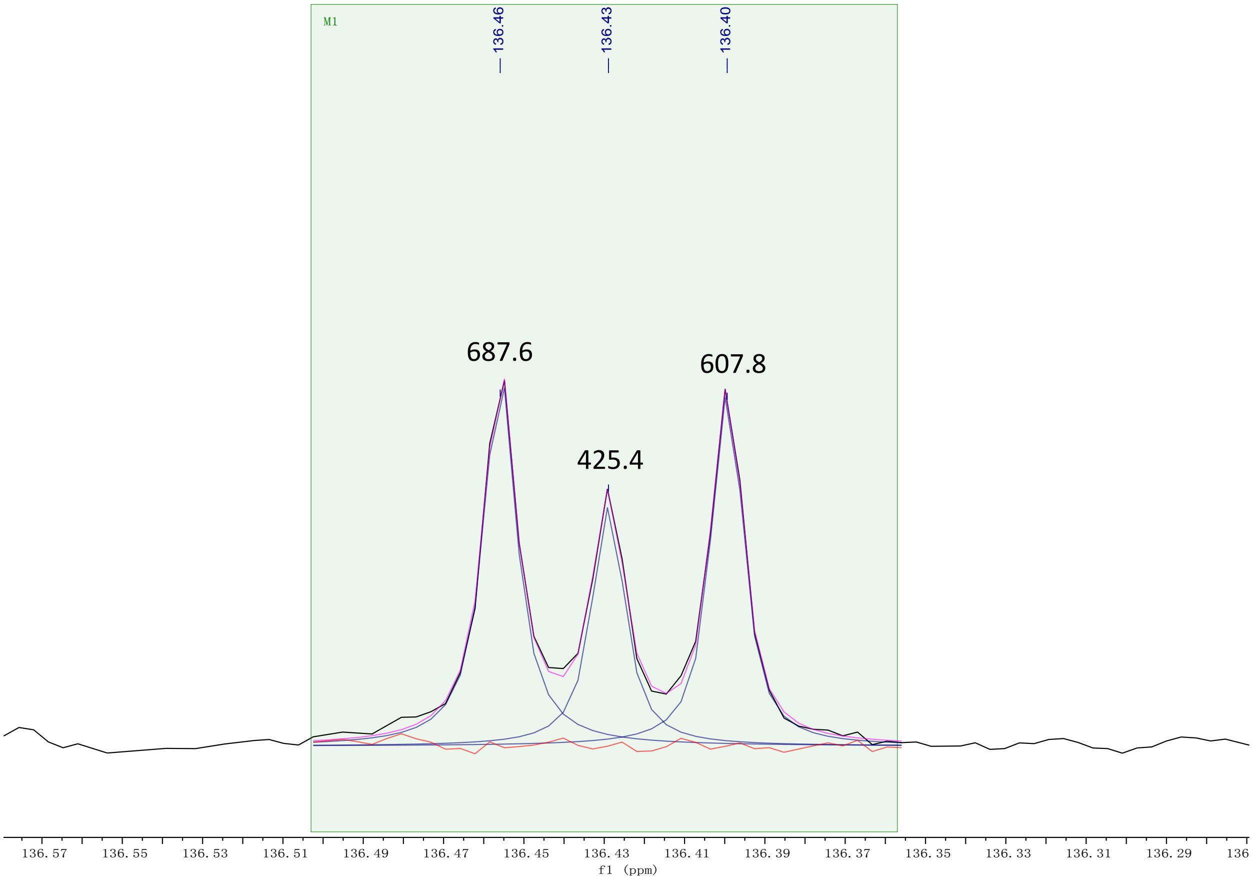

$$\textit{percentage of double } ^{15}N \textit{ labeled product} = \frac{687.6 + 607.8 - 425.4}{687.6 + 607.8 + 425.4} = 51\%$$

[^{15}N]-2-(4-trfluoromethylphenyl)-4,6-diphenyl-1,3,5-triazine (5e)

The analysis focused on the signal of the substituted carbon (a) on the phenyl ring:

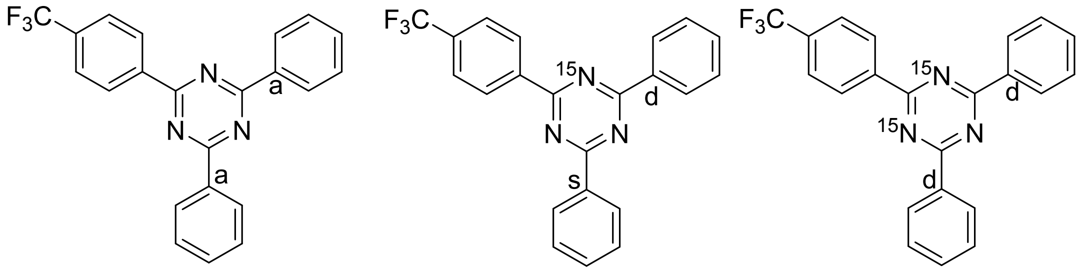

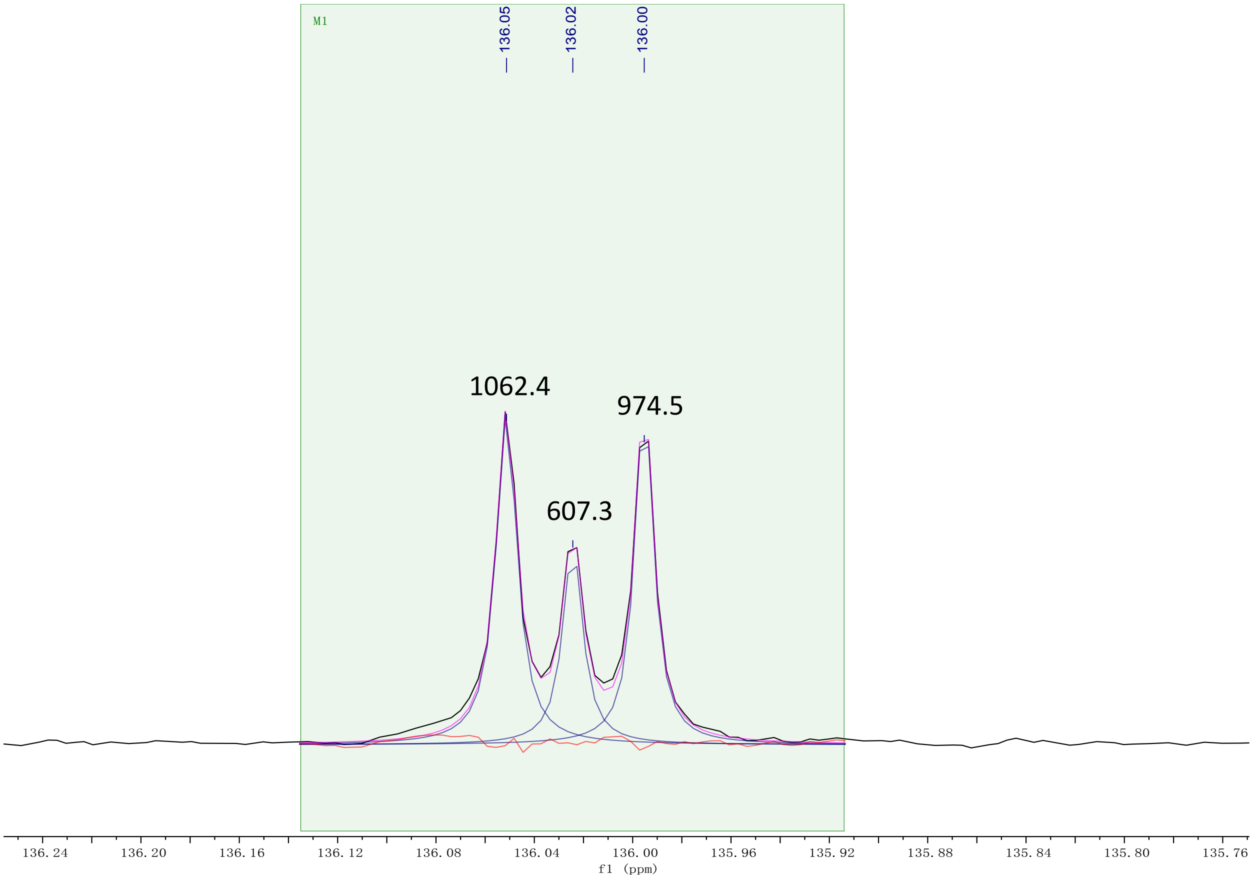

$$percentage\ of\ double\ ^{15}N\ labeled\ product = \frac{1062.4 + 974.5 - 607.3}{1062.4 + 974.5 + 607.3} = 54\%$$

III. Kinetic studies

The reactions between 4,6-diphenyl-1,2,3,5-tetrazine **1** (4 mM, 1.0 equiv) and amidines **8a-e** (8 mM, 2.0 equiv) were conducted in CD_3CN-$CDCl_3$ (V/V = 1:1) at 298 K. The conversion of starting materials and the formation of the products were monitored by ^1H NMR spectra. Clean conversion from reactant to product was observed with no intermediates or byproducts. The data points at low conversion range were observed to fit second-order reaction kinetics, and rate constants were fitted according to the following equation:

$$y = \frac{1}{[\boldsymbol{tetrazine}]_0 - [\boldsymbol{amidine}]_0} ln \frac{[\boldsymbol{amidine}]_0([\boldsymbol{tetrazine}]_0 - [\boldsymbol{triazine}])}{[\boldsymbol{tetrazine}]_0([\boldsymbol{amidine}]_0 - [\boldsymbol{triazine}])} = kt$$

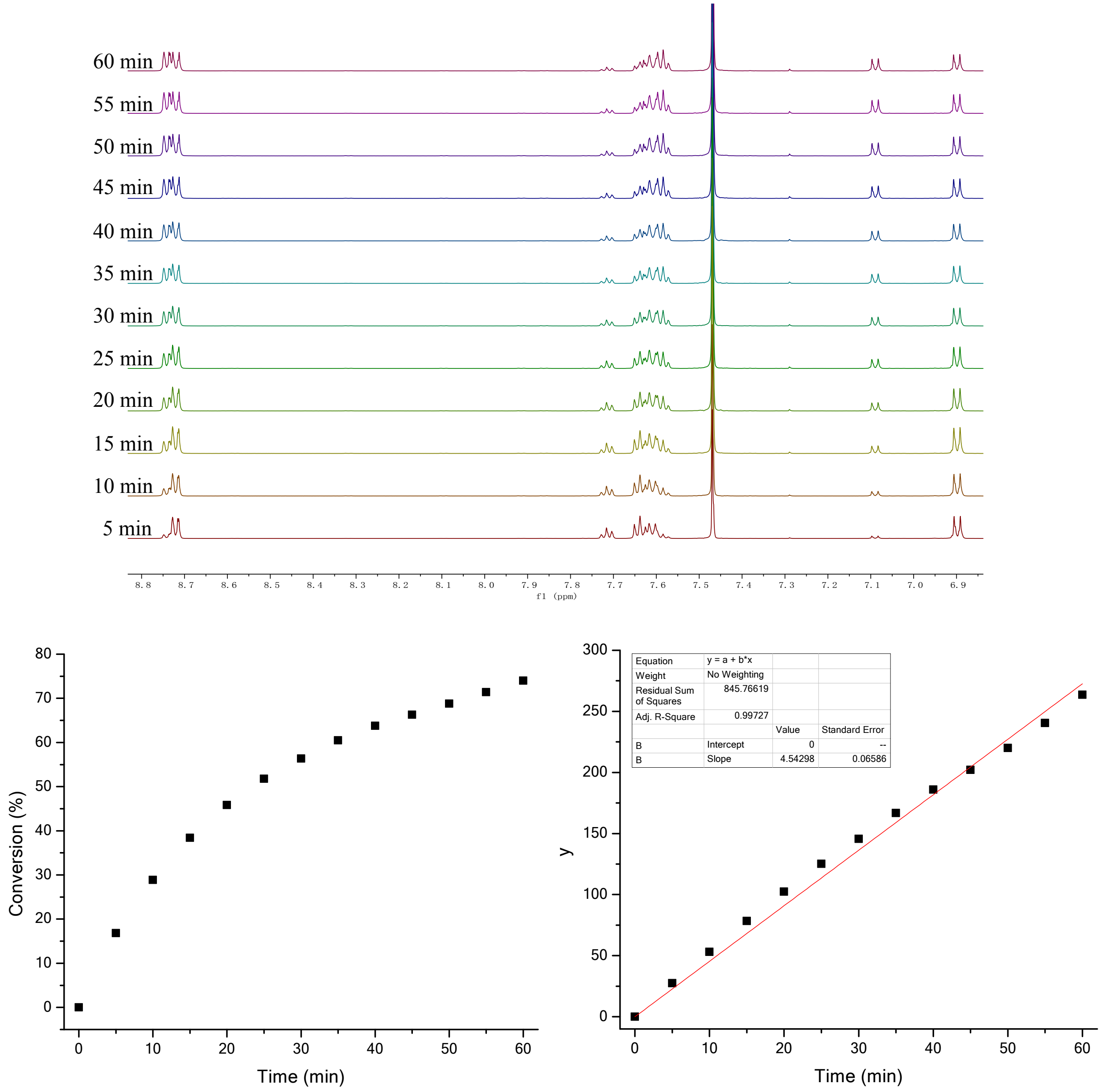

Figure S2. Kinetic studies of reaction between 4,6-diphenyl-1,2,3,5-tetrazine **1** and *p*-methoxybenzamidine **8a** (top: NMR spectra; bottom left: reaction progress over time; bottom right: linear fitting). k = (7.57 ± 0.10) × 10^{-2} $M^{-1}\cdot s^{-1}$ (R^2 = 0.997)

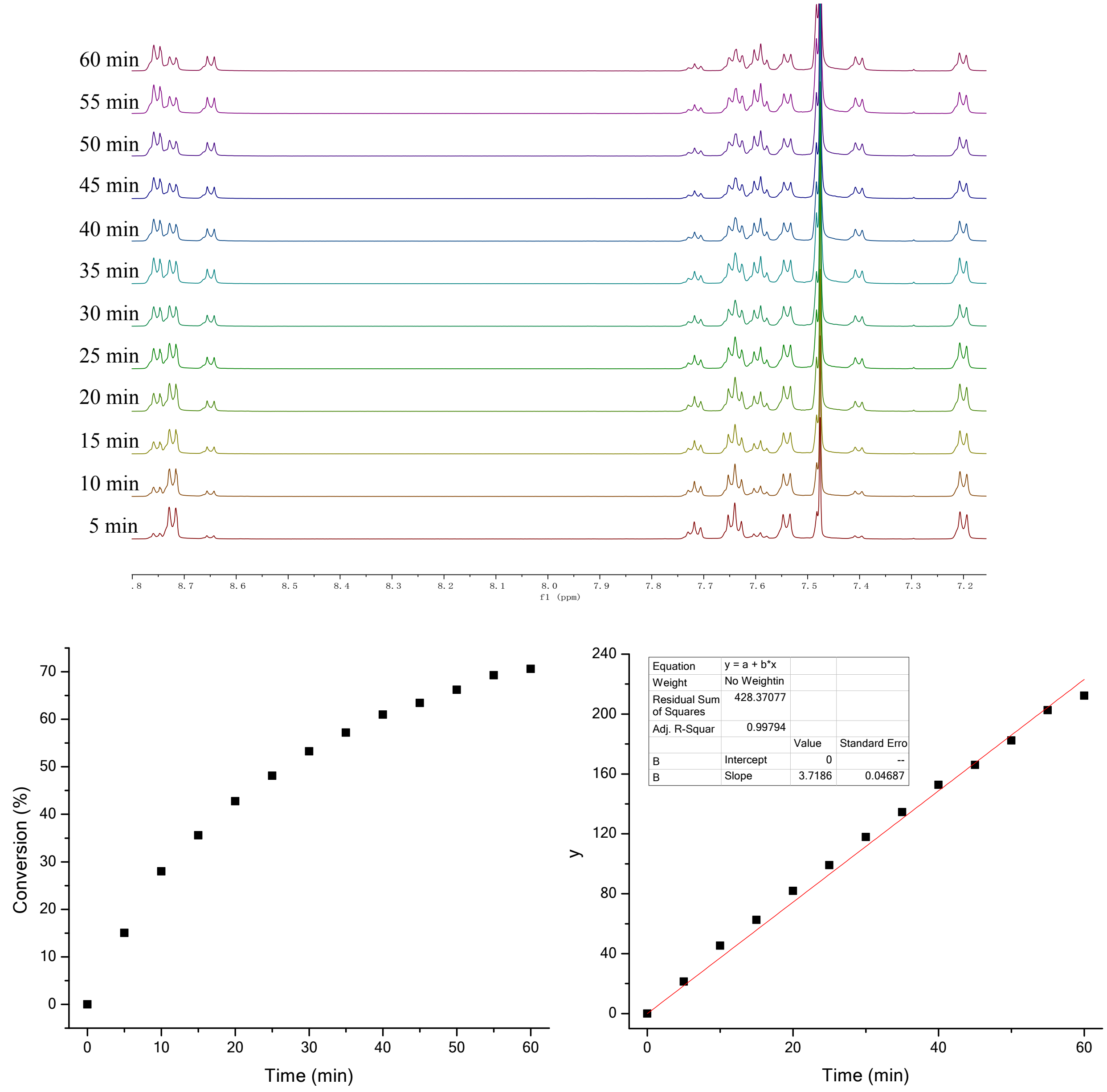

Figure S3. Kinetic studies of reaction between 4,6-diphenyl-1,2,3,5-tetrazine **1** and *p*-methylbenzamidine **8b** (top: NMR spectra; bottom left: reaction progress over time; bottom right: linear fitting). $k = (6.18 \pm 0.08) \times 10^{-2}\ M^{-1} \cdot s^{-1}$ ($R^2 = 0.998$)

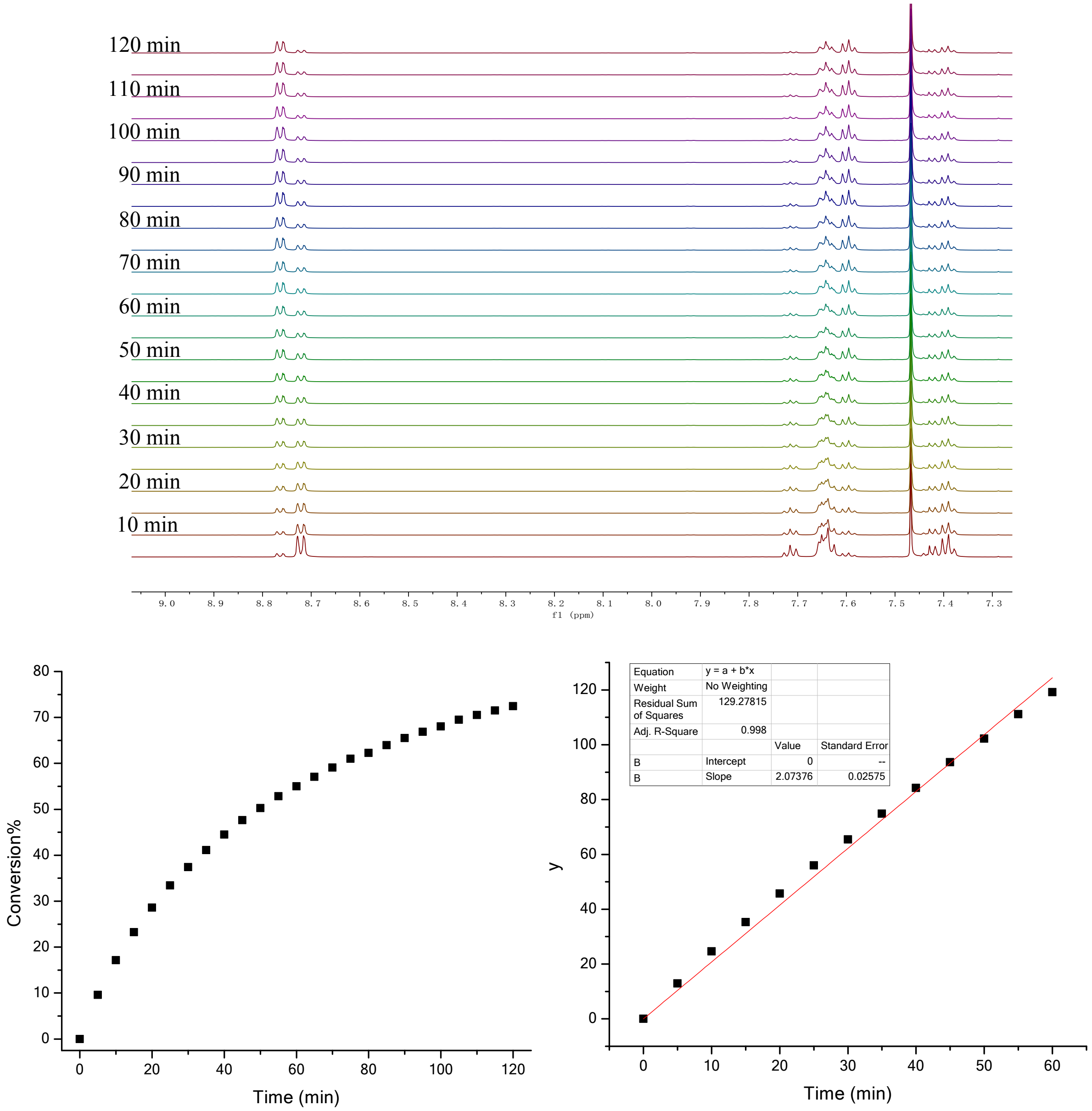

Figure S4. Kinetic studies of reaction between 4,6-diphenyl-1,2,3,5-tetrazine **1** and benzamidine **8c** (top: NMR spectra; bottom left: reaction progress over time; bottom right: linear fitting). k = $(3.45 \pm 0.04) \times 10^{-2}\ M^{-1}\cdot s^{-1}$ ($R^2 = 0.998$)

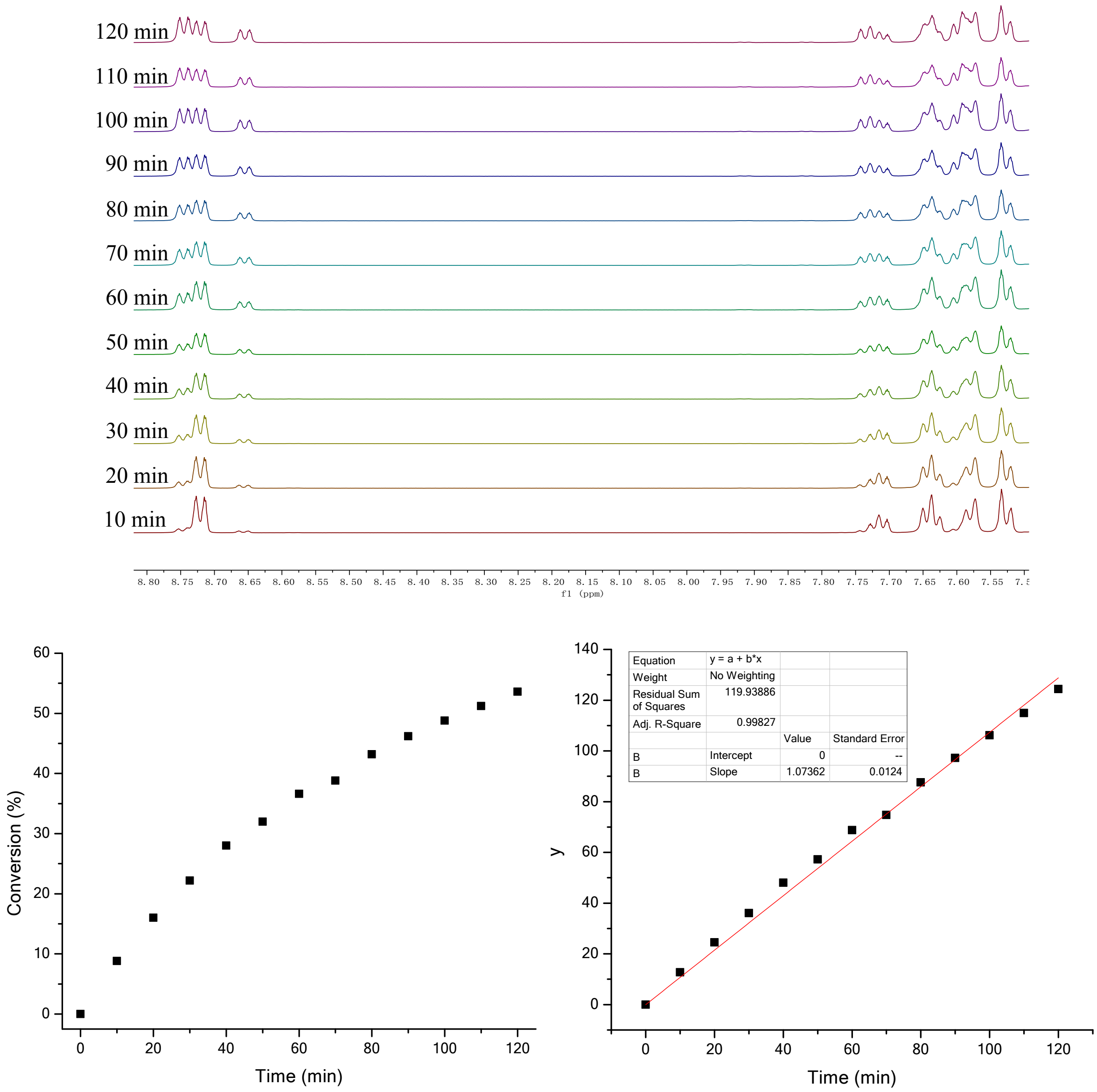

Figure S5. Kinetic studies of reaction between 4,6-diphenyl-1,2,3,5-tetrazine **1** and *p*-bromobenzamidine **8d** (top: NMR spectra; bottom left: reaction progress over time; bottom right: linear fitting). k = (1.79 ± 0.01) × 10^{-2} $M^{-1}\cdot s^{-1}$ (R^2 = 0.998)

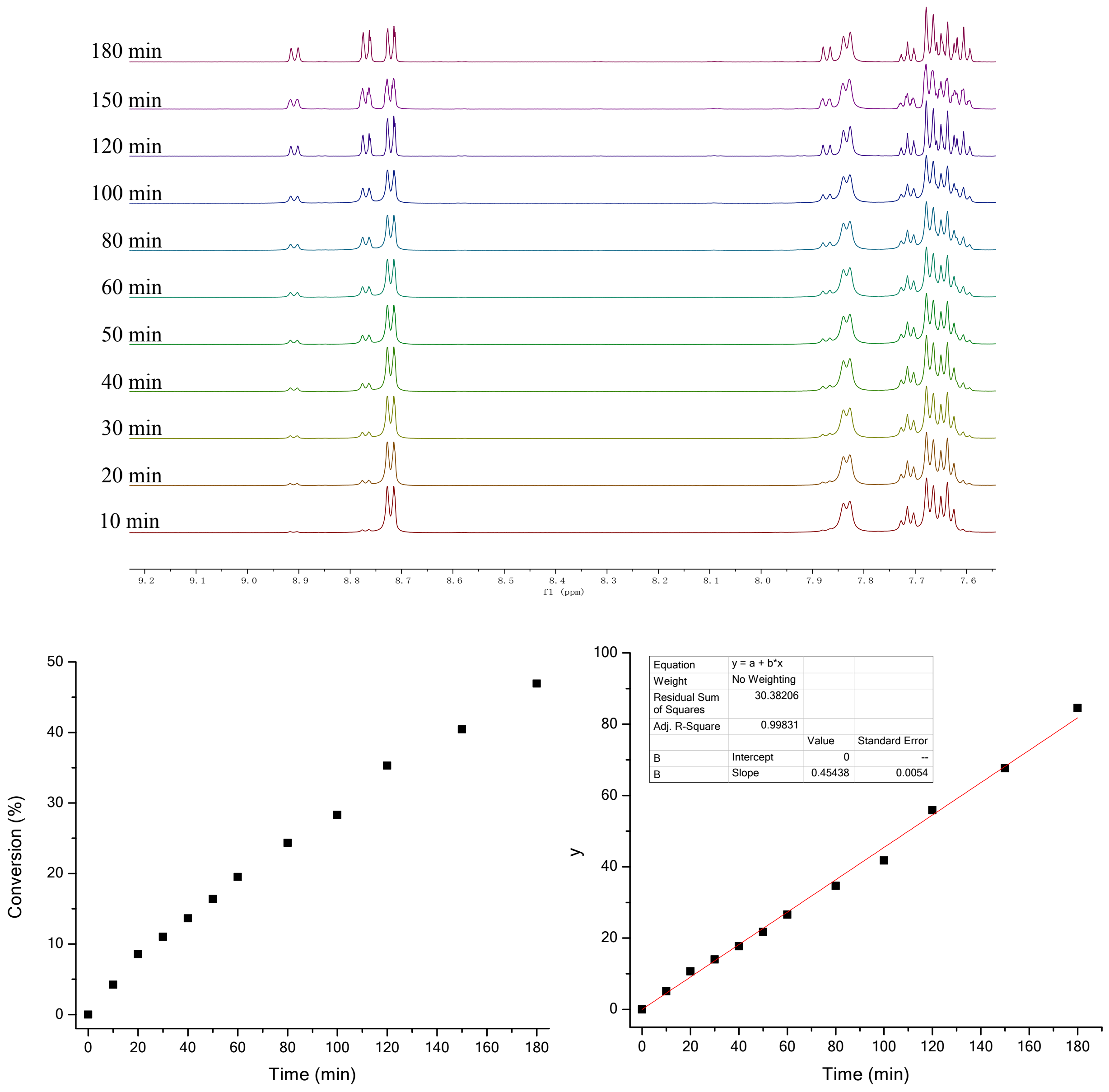

Figure S6. Kinetic studies of reaction between 4,6-diphenyl-1,2,3,5-tetrazine **1** and *p*-trifluoromethylbenzamidine **8e** (top: NMR spectra; bottom left: reaction progress over time; bottom right: linear fitting). k = (0.76 ± 0.01) × 10^{-2} $M^{-1}\cdot s^{-1}$ (R^2 = 0.998)

The reactions between 5-methoxycarbonyl-1,2,3-triazine **2** (0.68 mM, 1.0 equiv) and amidines **8a-e** (1.36 mM, 2.0 equiv) were conducted in CD_3CN-$CDCl_3$ (V/V = 1:1) at 298 K. The conversion of starting materials and the formation of the products were monitored by ^1H NMR spectra. Clean conversion from reactant to product was observed with no intermediates or byproducts. The data

points at low conversion range were observed to fit second-order reaction kinetics, and rate constants were fitted according to the following equation:

$$y = \frac{1}{[\boldsymbol{triazine}]_0 - [\boldsymbol{amidine}]_0} ln \frac{[\boldsymbol{amidine}]_0([\boldsymbol{triazine}]_0 - [\boldsymbol{diazine}])}{[\boldsymbol{triazine}]_0([\boldsymbol{amidine}]_0 - [\boldsymbol{diazine}])} = kt$$

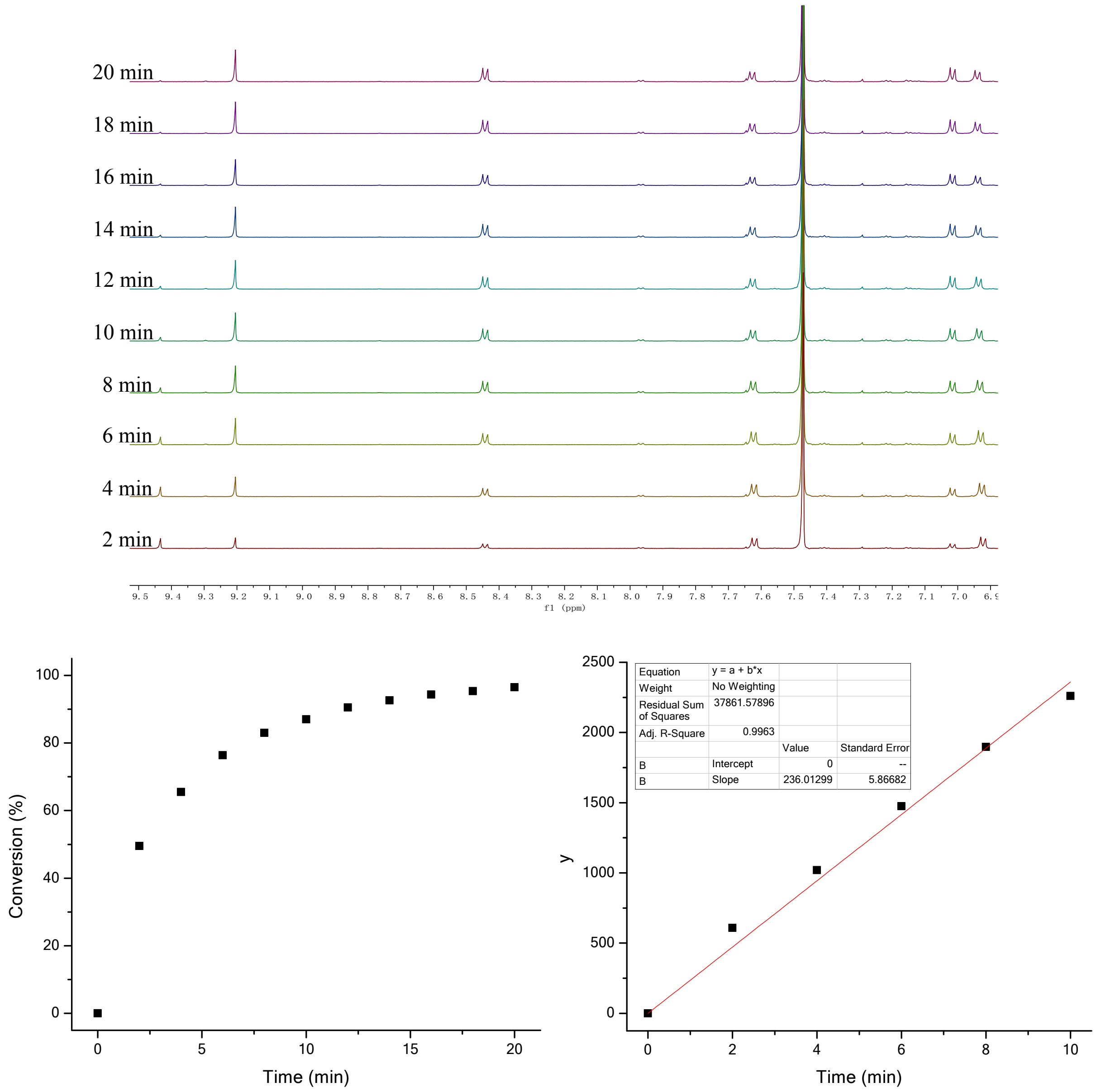

Figure S7. Kinetic studies of reaction between 5-methoxycarbonyl-1,2,3-triazine **2** and *p*-methoxybenzamidine **8a** (top: NMR spectra; bottom left: reaction progress over time; bottom right: linear fitting). k = 3.93 ± 0.10 $M^{-1} \cdot s^{-1}$ ($R^2 = 0.996$)

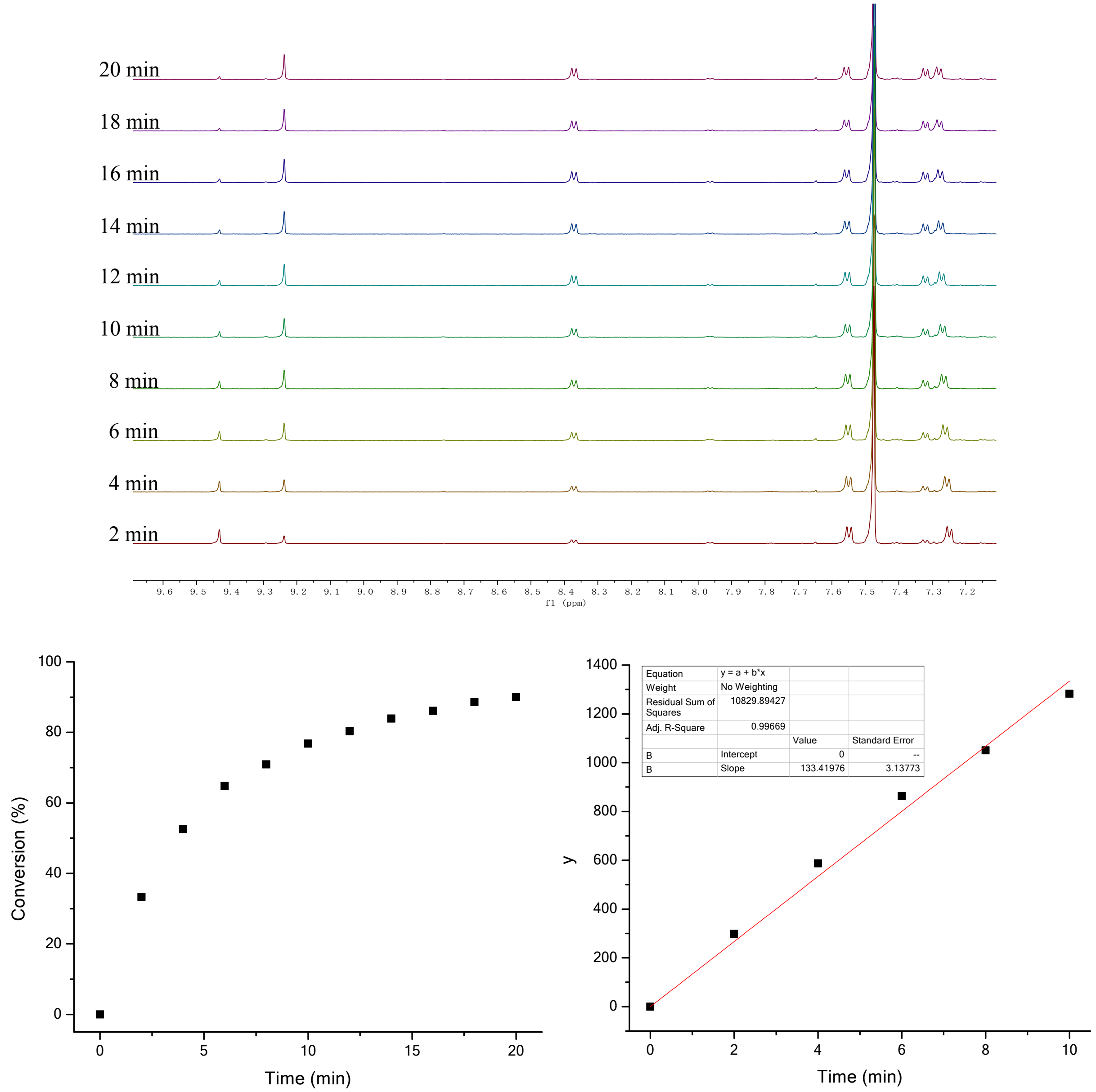

Figure S8. Kinetic studies of reaction between 5-methoxycarbonyl-1,2,3-triazine **2** and *p*-methylbenzamidine **8b** (top: NMR spectra; bottom left: reaction progress over time; bottom right: linear fitting). k = 2.22 ± 0.05 $M^{-1}\cdot s^{-1}$ (R^2 = 0.997)

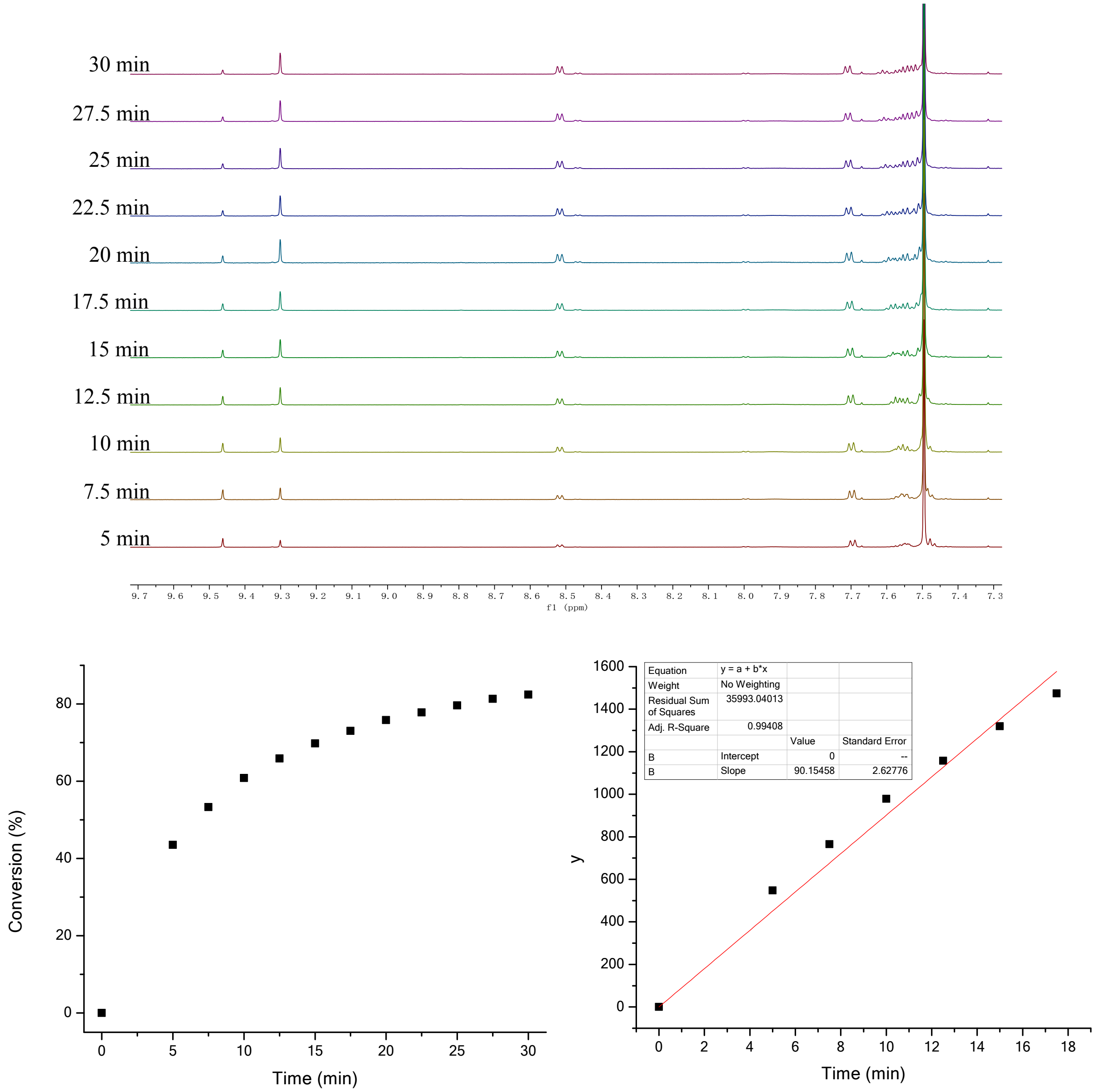

Figure S9. Kinetic studies of reaction between 5-methoxycarbonyl-1,2,3-triazine **2** and benzamidine **8c** (top: NMR spectra; bottom left: reaction progress over time; bottom right: linear fitting). $k = 1.50 \pm 0.04\ M^{-1} \cdot s^{-1}$ ($R^2 = 0.994$)

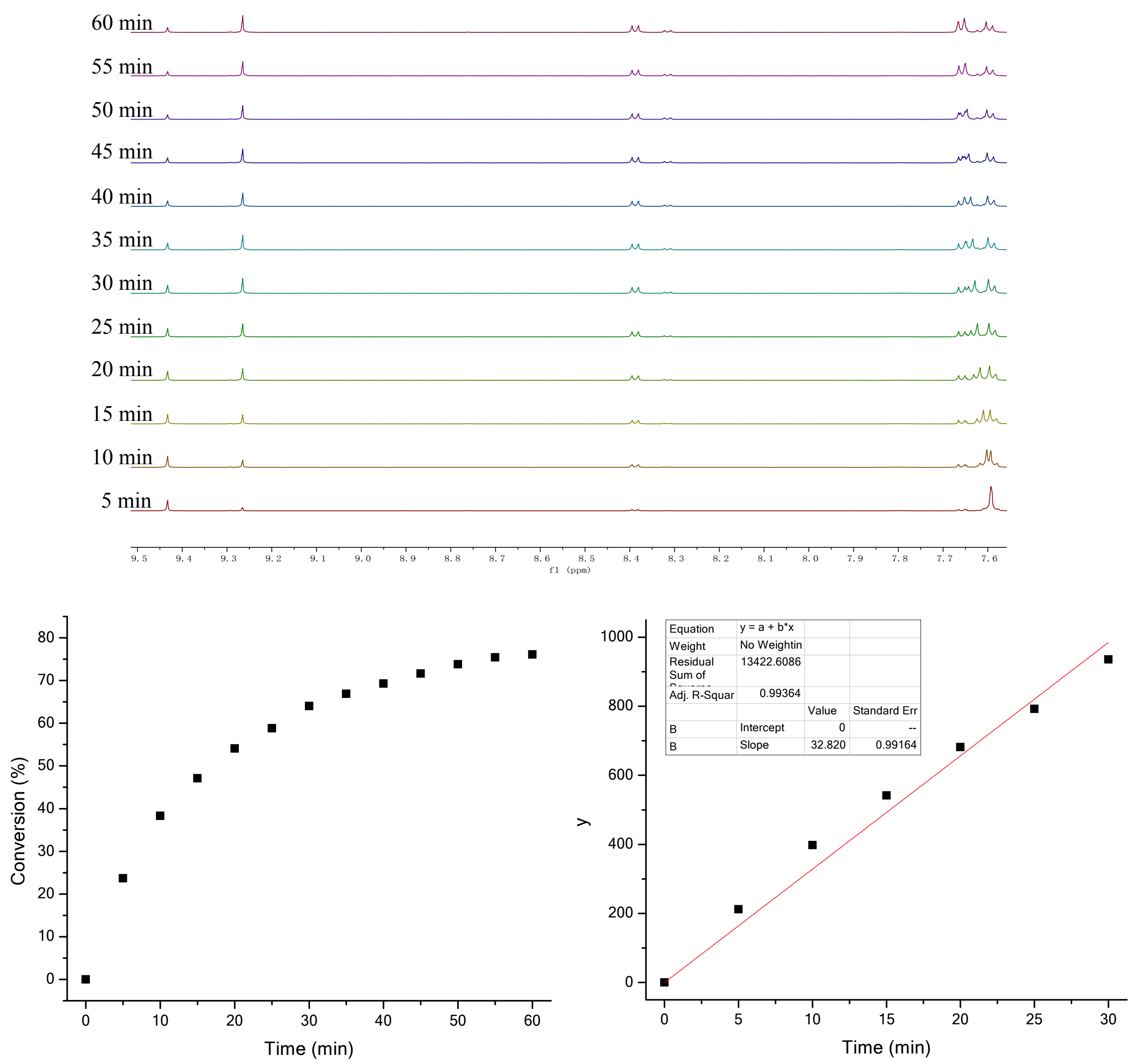

Figure S10. Kinetic studies of reaction between 5-methoxycarbonyl-1,2,3-triazine **2** and *p*-bromobenzamidine **8d** (top: NMR spectra; bottom left: reaction progress over time; bottom right: linear fitting). k = 0.55 ± 0.02 $M^{-1}\cdot s^{-1}$ (R^2 = 0.994)

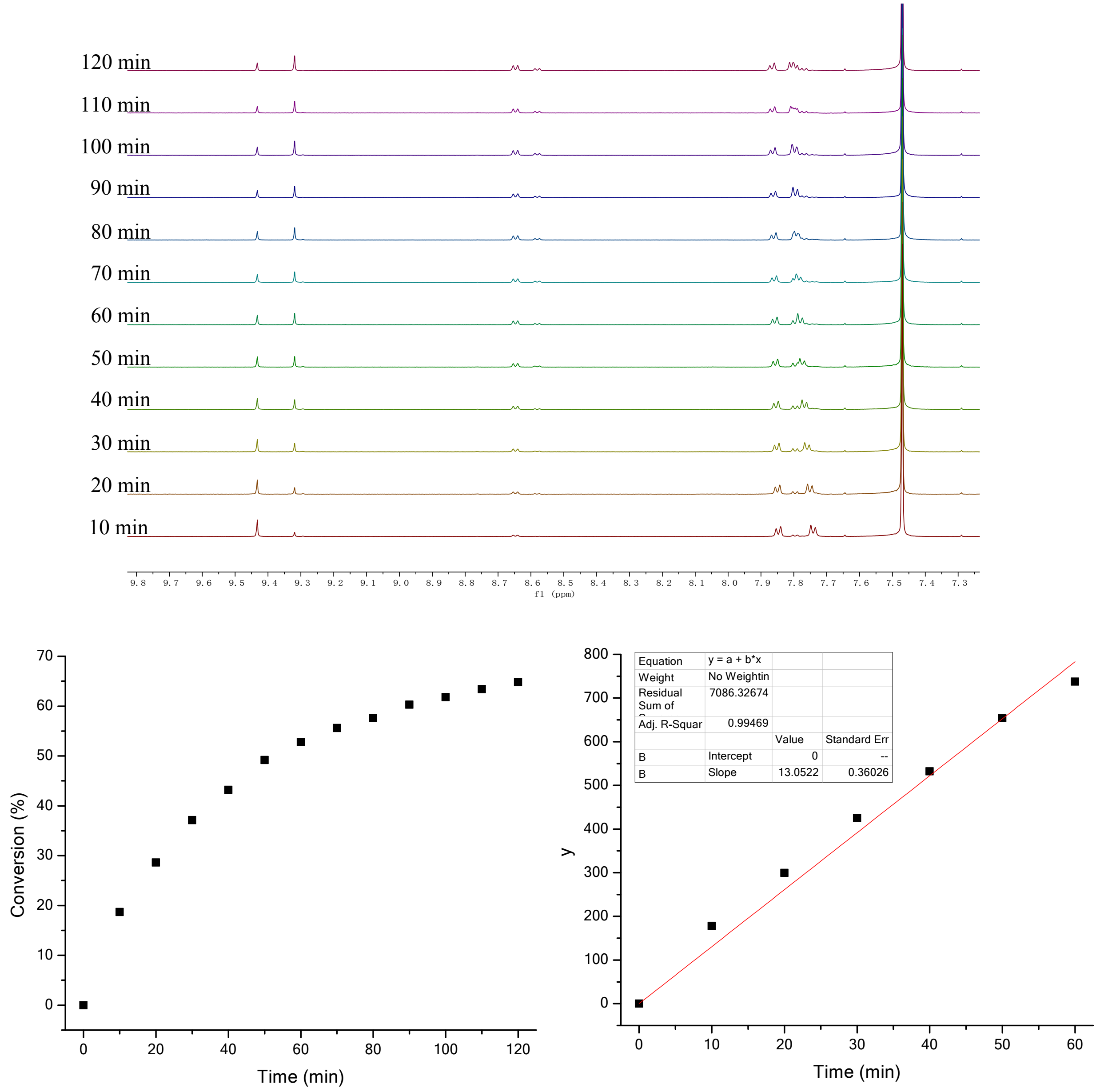

Figure S11. Kinetic studies of reaction between 5-methoxycarbonyl-1,2,3-triazine **2** and *p*-trifluorobenzamidine **8e** (top: NMR spectra; bottom left: reaction progress over time; bottom right: linear fitting). k = 0.22 ± 0.01 $M^{-1}\cdot s^{-1}$ ($R^2 = 0.995$)

IV. Kinetic Isotope Effect Studies

Preparation of methyl 4-methyl-1,2,3-triazine-5-carboxylate-6-*d* (6-*d*-triazine 9)

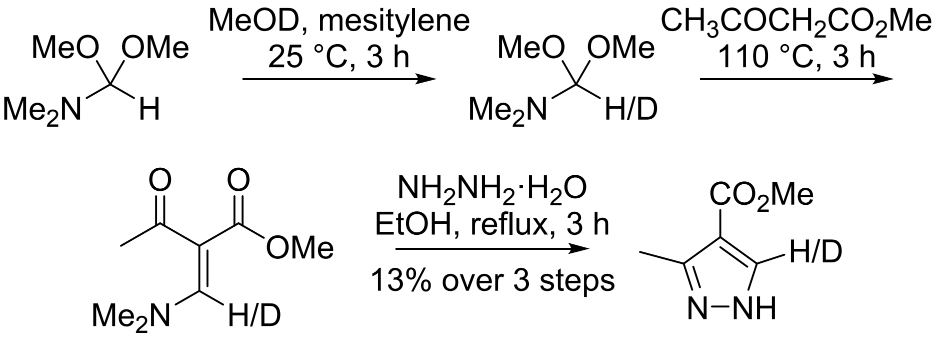

Partially deuterated dimethylformamide dimethyl acetal was prepared according to a literature procedure.[4] A solution of dimethylformamide dimethyl acetal (10 mL, 76 mmol, 1 equiv) in mesitylene (8 mL) in a 500 mL flame-dried round-bottom flask was treated with CH_3OD (20 mL, 500 mmol, 7 equiv). The mixture was stirred at 25 °C for 3 h, and CH_3OH/CH_3OD was distilled off (bath T = 120 °C). The mixture was cooled to 25 °C, and methyl acetoacetate (8.8 g, 76 mmol, 1 equiv) was added. The reaction mixture was then warmed at 110 °C for 3 h and cooled to 25 °C. The resulting mixture was treated with hydrazine hydrate (3.7 mL, 76 mmol, 1 equiv) in EtOH (150 mL), and stirred at reflux (bath temp = 110 °C). The reaction mixture was cooled to 25 °C and concentrated. Water (100 mL) was added to the residue and the mixture was extracted by EtOAc (100 mL × 3). The organic layers were combined, dried over Na_2SO_4, and concentrated. The residue was purified by column chromatography (SiO_2, 60% EtOAc/hexanes) to provide methyl 3-methyl-1*H*-pyrazole-4-carboxylate-5-*d* (1.50 g, 13% over 3 steps) as a white solid with 46% deuterium incorporation at C5 as determined by ^{1}H NMR: ^{1}H NMR (600 MHz, $CDCl_3$, 298K) δ 9.32 (s, br, 1H), 7.97 (s, 0.54H), 3.84 (s, 3H), 2.57 (s, 3H).

Methyl 4-methyl-1,2,3-triazine-5-carboxylate-6-*d* (6-*d*-triazine **9**) was prepared according to a literature preparation[5] of triazine **9** from methyl 3-methyl-1*H*-pyrazole-4-carboxylate-5-*d* to provide a purple oil (620 mg, 58%) with 44.63% deuterium incorporation at C6 as determined by quantitative ^{1}H NMR: ^{1}H NMR (600 MHz, CD_3CN, 298K) δ 9.23 (s, 0.55H), 3.96 (s, 3H), 2.91 (s, 3H).

Determination of $^{12}C/^{13}C$ Kinetic Isotope Effect in the Reaction of 9 with Benzamidine (8c)

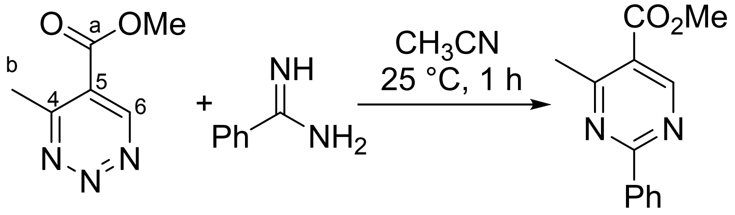

Large scale reaction between **9** and **8c**:

Methyl 4-methyl-1,2,3-triazine-5-carboxylate **9** (765 mg, 5 mmol, 1 equiv) was dissolved in CH_3CN (100 mL). Anisole (0.5 mL) was added as an internal standard. A solution of benzamidine (**8c**, 4 mmol, 480 mg, 0.8 equiv) in CH_3CN (5 mL) was added dropwise, and the mixture was stirred at 25 °C for 1 h when full conversion was observed. LC-MS analysis of the mixture before and after the reaction was conducted to determine the conversion. The mixture was concentrated and the residue was purified by column chromatography (SiO_2, 75% Et_2O/hexanes) to provide recovered **9** (123 mg, 16%) as a purple oil for NMR analysis.

Quantitative ^{13}C NMR measurement:

The NMR samples of starting and recovered material were prepared identically, filling 10 mm NMR tubes to a constant height of 5 cm with a $CDCl_3$ solution of 100 mg material. T_1 determination by the inversion-recovery method was carried out on each NMR sample. The ^{13}C spectra were recorded at 150.90 MHz on a Bruker Avance III HD 600 MHz spectrometer at room temperature, with inverse gated decoupling and 65 s ($>5T_1$) delays between calibrated pulses. 6 independent spectra of each sample were recorded, and integrations were determined numerically using a constant region for each peak that was 10 times the peak width at half height distance on either side of the peak. The integrations of the methyl carbon on the ester were set at 1000. The average integrations for the other carbons are shown in Table S2.

Table S2. $^{12}C/^{13}C$ kinetic isotope effect determination.

Chemical shift (ppm)	163.7	159.1	147.7	119.0	53.4	21.4
Carbon	C_a	C4	C6	C5	methoxy	C_b
Starting material (n = 6)						
Intensity	977.8	1001.8	981.7	983.5	1000	1047.2
Standard deviation	0.15%	0.18%	0.13%	0.22%	0.00%	0.23%
Reaction 1 (n = 6) (conversion: 65% ± 2%)						
Intensity	984.7	1006.2	1019.4	992.1	1000	1038.8
Standard deviation	0.48%	0.37%	0.45%	0.31%	0.00%	0.27%
R/R_0						
Value	1.007	1.004	1.038	1.009	1.000	0.991
Standard deviation	0.51%	0.41%	0.46%	0.38%	0.00%	0.36%
$^{12}C/^{13}C$ KIE						
KIE	1.007	1.004	1.039	1.009	1.000	0.992
Standard deviation	0.006	0.004	0.007	0.004	0.000	0.004
Reaction 2 (n = 6) (conversion: 80% ± 2%)						
Intensity	982.7	1014.4	1039.8	998.6	1000	1030.7
Standard deviation	0.22%	0.17%	0.12%	0.16%	0.00%	0.18%
R/R_0						
Value	1.005	1.012	1.059	1.015	1.000	0.984
Standard deviation	0.26%	0.25%	0.17%	0.27%	0.00%	0.29%
$^{12}C/^{13}C$ KIE						
KIE	1.003	1.008	1.037	1.009	1.000	0.990
Standard deviation	0.003	0.003	0.005	0.004	0.000	0.004
$^{12}C/^{13}C$ KIE (average)						
KIE (average)	1.005	1.006	1.038	1.009	1.000	0.991
Standard deviation	0.006	0.004	0.007	0.004	0.000	0.005

$$\text{Calculation of KIE: } KIE = \frac{\ln(1-F)}{\ln[\frac{(1-F)R}{R_0}]}$$

Where R is the ^{13}C proportion of the recovered starting material, R_0 is ^{13}C proportion of the starting material, and F is the conversion of the material.

Determination of $^1H/^2H$ Kinetic Isotope Effect for the Reaction of 9 with Benzamidine (8c)

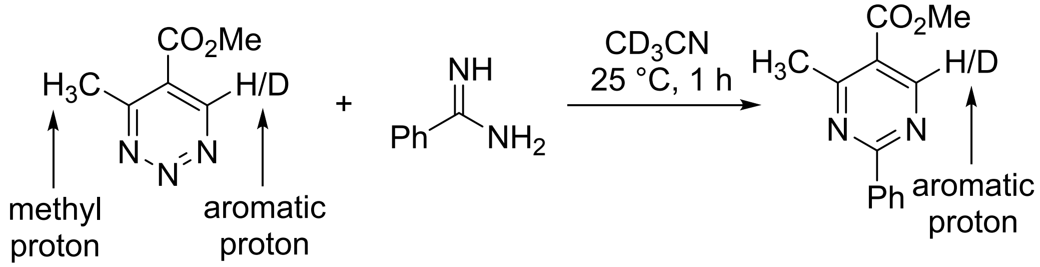

Methyl 4-methyl-1,2,3-triazine-5-carboxylate-6-*d* (6-*d*-triazine **9**, 30.8 mg, 0.2 mmol, 1 equiv, 44.63% deuterium incorporation at C6) was dissolved in 1.5 mL CD_3CN. A solution of benzamidine (**8c**, 16.8 mg, 0.14 mmol, 0.7 equiv) in CD_3CN was added dropwise at 25 °C. The mixture was stirred at 25 °C for 1 h when full conversion was observed. The mixture was directly subjected to ^1H NMR analysis.

The NMR samples of starting material and reaction mixture were prepared and filling 10 mm NMR tubes to a constant height of 5 cm. T_1 determination by the inversion-recovery method was carried out on each NMR sample. The ^1H spectra were recorded at 600.13 MHz on a Bruker Avance III HD 600 MHz spectrometer at room temperature, with 40 s (>$5T_1$) delays between calibrated pulses. Six (6) independent spectra of each sample were recorded, and the integrations were determined numerically using a constant region for each peak that was 10 times the peak width at half height distance on either side of the peak. The integration of the methyl protons on the triazine were set at 3000. The average integrations for the aromatic proton of starting material and reaction mixture are shown in Table S3. The conversion was determined through the integration of aromatic protons of 1,2,3-triazine and pyrimidine.

Table S3. $^1H/^2H$ kinetic isotope effect determination.

Starting material (n = 6)			
Signal	Triazine aromatic proton (δ = 9.23)		Triazine methyl proton (δ = 2.91)
Intensity	553.68		3000
Standard Deviation	0.49		0
Deuterium Proportion	44.63%		
Standard Deviation	0.05%		
Reaction 1 (n = 6)			
Signal	Triazine aromatic proton (δ = 9.23)	Pyrimidine aromatic proton (δ = 9.15)	Triazine methyl proton (δ = 2.91)
Intensity	606.95	1743.91	3000
Standard Deviation	2.02	10.5	0
Conversion	74.2%		
Standard Deviation	0.8%		
Deuterium Proportion	39.33%		
Standard Deviation	0.20%		
R/R_0	0.8033		
Standard Deviation	0.0040		
KIE	0.861		
Standard Deviation	0.005		
Reaction 2 (n = 6)			
Signal	Triazine aromatic proton (δ = 9.23)	Pyrimidine aromatic proton (δ = 9.15)	Triazine methyl proton (δ = 2.91)
Intensity	624.81	3020.70	3000
Standard Deviation	4.40	12.1	0
Conversion	82.9%		
Standard Deviation	0.6%		
Deuterium Proportion	37.52%		
Standard Deviation	0.44%		
R/R_0	0.7449		
Standard Deviation	0.0074		
KIE	0.857		
Standard Deviation	0.006		
KIE (average)	0.859		
Standard Deviation	0.008		

$$\text{Calculation of KIE: } KIE = \frac{\ln(1-F)}{\ln[\frac{(1-F)R}{R_0}]}$$

Where R is the deuterium proportion of the recovered starting material, R_0 is deuterium proportion of the starting material, and F is the conversion of the protium proportion of the material.

IV. Computational Studies

Methods

DFT calculations were performed using Gaussian 09 Rev D.01.[6] The M06-2X density functional[7] was employed in combination with D3 empirical dispersion correction,[8] as proposed by Grimme and coworkers.[9] Single imaginary frequencies corresponding to the desired reaction coordinates were obtained only in the case of transition state (TS) calculations. No imaginary frequencies were obtained for all other structures. A quasi-harmonic correction to entropy was applied by setting all frequencies below 100 cm^{-1} to 100 cm^{-1} using GoodVibes.[10]

Conformer searches of all structures, including transition states, were performed using CREST 2.7.1 with the iMTD-GC workflow and the GFN2-xTB method.[11-12] Conformers found by CREST were then optimized using M06-2X-D3/def2-TZV-SMD(acetonitrile). The lowest energy conformer was reoptimized using M06-2X-D3/def2-TZVP-SMD(acetonitrile). Structures were visualized using CYLview.[13]

Tunneling behavior was estimated with the correction by Skodje and coworkers[14] using the PyTUN utility (https://github.com/SJ-Ang/PyTUN). The tunneling correction was converted into an apparent ΔG lowering using the Eyring equation at 298.15 K.

For computational determination of KIEs the atom was interest was substituted by the heavier isotope in both the starting materials and the transition state and a frequency/thermodynamic analysis was performed. Barrier heights were calculated and compared to the parent system without isotope substitution. $\Delta\Delta G^{‡}$ were converted into relative rates using the Eyring equation at 298.15 K.

Additional retro-Diels–Alder pathways

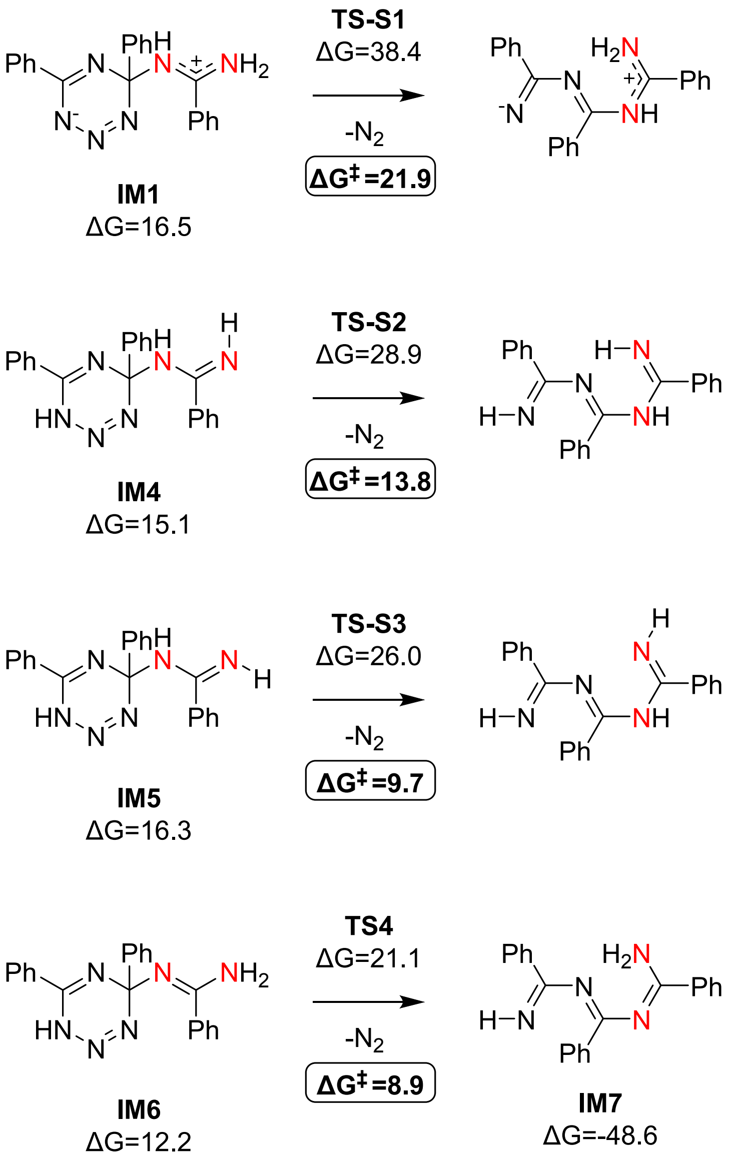

Figure S12. Alternative retro-Diels–Alder pathways in the reaction between **1** and **4c**. Energies relative to starting materials.

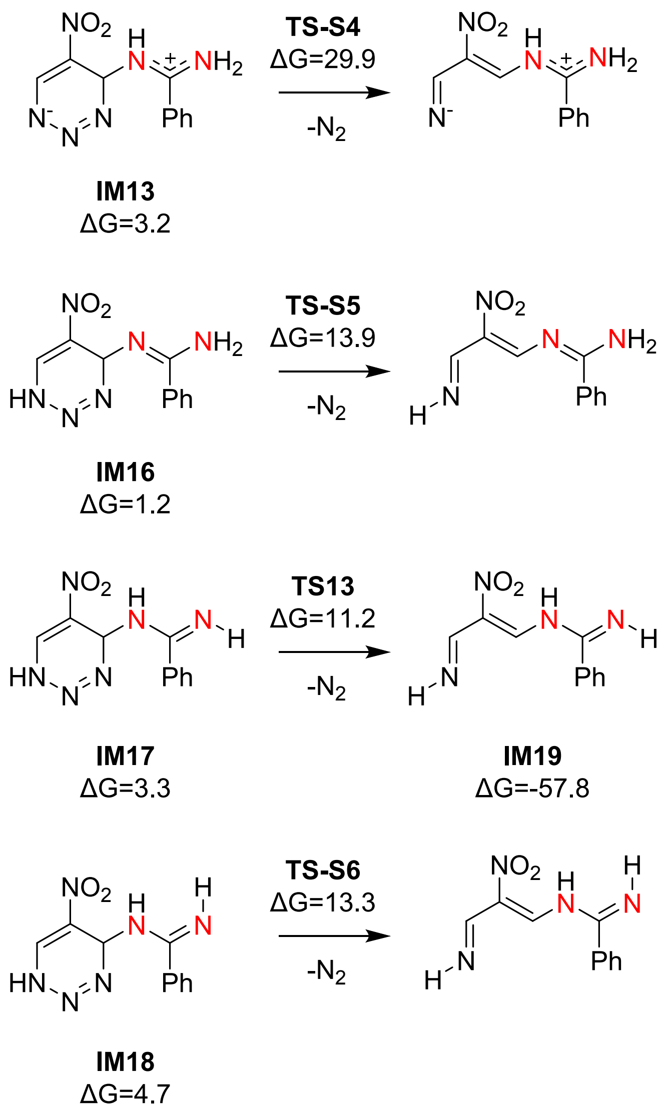

Figure S13. Alternative retro-Diels–Alder pathways in the reaction between **3** and **4c**. Energies relative to starting materials.

Proton tunneling in TS5, TS7, and TS14

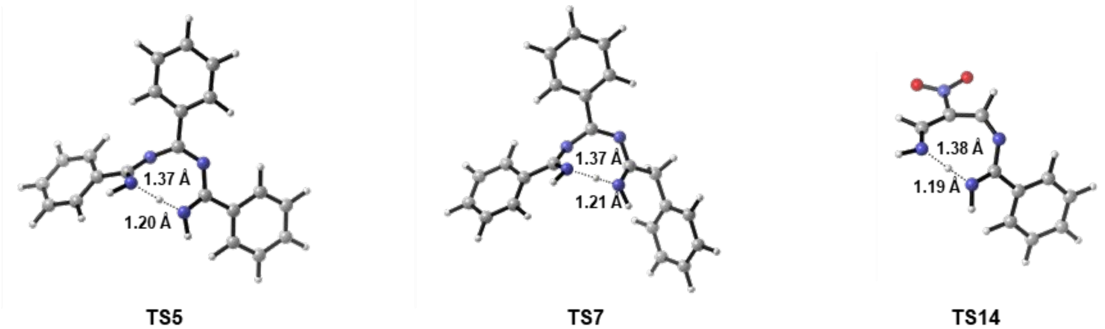

TS5	TS7	TS14
v = -982 cm^{-1}	v = -1037 cm^{-1}	v = -848 cm^{-1}
tunneling correction= 3.37	tunneling correction= 4.19	tunneling correction= 2.29
ΔG_{uncorr} = -38.9 kcal mol^{-1}	ΔG_{uncorr} = -38.4 kcal mol^{-1}	ΔG_{uncorr} = -47.3 kcal mol^{-1}
ΔG_{corr} = -39.6 kcal mol^{-1}	ΔG_{corr} = -39.3 kcal mol^{-1}	ΔG_{corr} = -47.8 kcal mol^{-1}

Figure S14. Tunneling corrections for **TS5**, **TS7**, and **TS14**.

Kinetic isotope effects

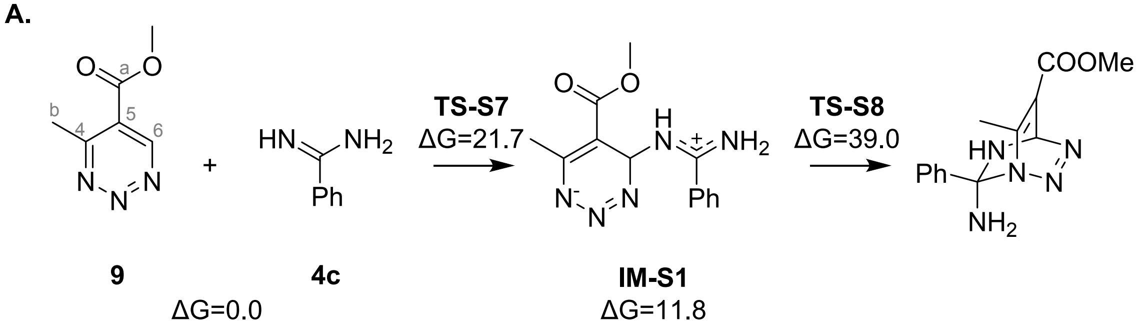

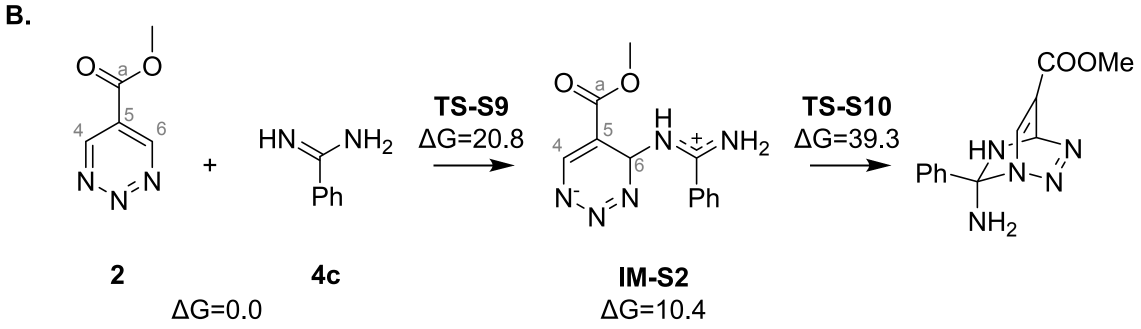

Figure S15. Energy barriers for **A. 4c** + **9** and **B. 4c** + **2**.

Table S4: M06-2X-D3/def2-TZVP-SMD(acetonitrile) calculated Gibbs free energies for determination of KIEs on key atoms in the reactions **4c** + **9** and **4c** + **2**.

Nucleophilic attack **4c** + **9**

	**4c** G(298.15) / hartree	**9** G(298.15) / hartree	**TS-S7** G(298.15) / hartree
no heavy isotopes	-380.956545	-547.399970	-928.320158
C4-^{13}C	-380.956545	-547.400240	-928.320156
C5-^{13}C	-380.956545	-547.400237	-928.320109
C6-^{13}C	-380.956545	-547.400229	-928.320173
C_a-^{13}C	-380.956545	-547.400258	-928.320126
C_b-^{13}C	-380.956545	-547.400207	-928.320158
C6-^{2}H	-380.956545	-547.403421	-928.323489

Diels–Alder ringclosure **4c** + **9**

	**IM-S1**<br>G(298.15) / hartree	**TS-S8**<br>G(298.15) / hartree
no heavy isotopes	-928.336975	-928.293484
C4-^{13}C	-928.337242	-928.293752
C5-^{13}C	-928.337239	-928.293753
C6-^{13}C	-928.337236	-928.293744
C_a-^{13}C	-928.337259	-928.293768
C_b-^{13}C	-928.337202	-928.293713
C6-^{2}H	-928.340613	-928.297121

Nucleophilic attack **4c** + **2**

	**4c**<br>G(298.15) / hartree	**2**<br>G(298.15) / hartree	**TS-S9**<br>G(298.15) / hartree
no heavy isotopes	-380.956545	-508.110381	-889.033774
C4-^{13}C	-380.956545	-889.033994	-508.110643
C5-^{13}C	-380.956545	-889.034042	-508.110653
C6-^{13}C	-380.956545	-889.034031	-508.110641
C_a-^{13}C	-380.956545	-889.034061	-508.110673
C4-^{2}H	-380.956545	-889.037206	-508.113867
C6-^{2}H	-380.956545	-889.037342	-508.113836

Diels–Alder ringclosure **4c** + **2**

	**IM-S2**<br>G(298.15) / hartree	**TS-S10**<br>G(298.15) / hartree
no heavy isotopes	-889.050347	-889.004328
C4-^{13}C	-889.050607	-889.004588
C5-^{13}C	-889.050611	-889.004597
C6-^{13}C	-889.050602	-889.004587
C_a-^{13}C	-889.050631	-889.004615
C4-^{2}H	-889.053967	-889.007934
C6-^{2}H	-889.053749	-889.007686

Computational Data

The calculated energies for the lowest energy conformers are summarized in Table S5. Coordinates of lowest energy conformers are provided as *.xyz files.

Table S5: M06-2X-D3/def2-TZVP-SMD(acetonitrile) calculated energies of lowest energy conformers. Quasiharmonic correction was applied for Gibbs free energies.

Structure	E / hartree	ZPE / hartree	H / hartree	G(298.15) / hartree
**1**	-758.454580	0.215342	-758.225136	-758.278679
**3**	-484.801746	0.067153	-484.727014	-484.765965
**4c**	-381.065388	0.140748	-380.916149	-380.956540
**5a**	-1012.847178	0.337968	-1012.488897	-1012.554563
**5c**	-973.540656	0.309410	-973.213179	-973.274777
**7c**	-699.888118	0.161904	-699.714101	-699.763204
**N_2**	-109.530714	0.005748	-109.521662	-109.543381
**NH_3**	-56.556070	0.034178	-56.518081	-56.539922
**IM1**	-1139.522252	0.360037	-1139.140155	-1139.208971
**IM2**	-1139.492609	0.360220	-1139.111053	-1139.177762
**IM3**	-1030.086497	0.350689	-1029.715321	-1029.781077
**IM4**	-1139.524463	0.360105	-1139.142267	-1139.211227
**IM5**	-1139.522601	0.360451	-1139.139913	-1139.209207
**IM6**	-1139.529006	0.360097	-1139.146550	-1139.215850
**IM7**	-1030.070869	0.347799	-1029.701611	-1029.769286
**IM8**	-1030.073121	0.348927	-1029.702999	-1029.769936
**IM9**	-1069.382510	0.376241	-1068.983416	-1069.053639
**IM10**	-1069.381985	0.377267	-1068.983259	-1069.051112
**IM11**	-1069.403235	0.378833	-1069.002690	-1069.070592
**IM12**	-1069.396575	0.378174	-1068.996521	-1069.064573
**IM13**	-865.889554	0.211882	-865.663126	-865.717412
**IM14**	-865.842470	0.211864	-865.615773	-865.669874
**IM15**	-756.440992	0.202429	-756.224838	-756.276818
**IM16**	-865.891369	0.211899	-865.663539	-865.720542
**IM17**	-865.889711	0.213108	-865.661178	-865.717183
**IM18**	-865.887076	0.212788	-865.658748	-865.715019
**IM19**	-756.431147	0.200332	-756.215887	-756.270860
**IM20**	-756.421580	0.199090	-756.207183	-756.262579
**IM21**	-756.423722	0.200336	-756.208574	-756.262970
**IM22**	-756.452900	0.203025	-756.235978	-756.288815
**TS1**	-1139.508266	0.356724	-1139.129152	-1139.198745
**TS2**	-1139.486759	0.358704	-1139.106959	-1139.173470
**TS3**	-1139.490542	0.357370	-1139.111708	-1139.178878
**TS4**	-1139.510319	0.355925	-1139.131860	-1139.201518
**TS5**	-1030.051975	0.343769	-1029.687628	-1029.753896
**TS6**	-1030.051641	0.346910	-1029.683947	-1029.750313
**TS7**	-1069.361625	0.371828	-1068.967627	-1069.036806

**TS8**	-1069.367137	0.375398	-1068.969817	-1069.038034
**TS9**	-1069.362397	0.375122	-1068.965122	-1069.033951
**TS10**	-865.863188	0.209012	-865.638312	-865.695018
**TS11**	-865.838135	0.211518	-865.612247	-865.665773
**TS12**	-865.841961	0.210449	-865.616907	-865.670624
**TS13**	-865.873996	0.209961	-865.648517	-865.704725
**TS14**	-756.411305	0.195829	-756.201374	-756.254525
**TS15**	-756.414431	0.198744	-756.201556	-756.254241
**TS16**	-756.404174	0.199671	-756.190401	-756.243647
**TS-S1**	-1139.482714	0.356057	-1139.104076	-1139.173981
**TS-S2**	-1139.499852	0.357533	-1139.120168	-1139.189161
**TS-S3**	-1139.504309	0.357343	-1139.124827	-1139.193804
**TS-S4**	-865.842482	0.208626	-865.618034	-865.674782
**TS-S5**	-865.867505	0.208181	-865.643310	-865.700281
**TS-S6**	-865.870417	0.209787	-865.645108	-865.701275

V. References

1. Charushin, V. N.; Van der Plas, H. C., Ring transformations in reactions of heterocyclic compounds with nucleophiles. Part 26. 1,3- and 1,4-Cyclo adducts as intermediates in the pyrimidine to pyridine ring transformation of 5-nitropyrimidines by .alpha.-phenylacetamidines. *The Journal of Organic Chemistry* **2002,** *48* (16), 2667-2671.
2. Wu, Z. C.; Boger, D. L., Synthesis, Characterization, and Cycloaddition Reactivity of a Monocyclic Aromatic 1,2,3,5-Tetrazine. *Journal of the American Chemical Society* **2019,** *141* (41), 16388-16397.
3. Glinkerman, C. M.; Boger, D. L., Cycloadditions of 1,2,3-Triazines Bearing C5-Electron Donating Substituents: Robust Pyrimidine Synthesis. *Organic Letters* **2015,** *17* (16), 4002-4005.
4. Choi, C.; Carlo, A. A.; Cronin, C. N.; Jing, K.; Kung, D. W.; Liu, J.; Lombardo, V. M.; Turco, A. R.; Yin, J.; Yu, A.; Wright, S. W., Synthesis of Deuterated Heterocycles with Me2NCD(OMe)2 and Evaluation of the Products for Metabolism by Aldehyde Oxidase. *ACS Med Chem Lett* **2022,** *13* (2), 250-256.
5. Quinones, R. E.; Wu, Z. C.; Boger, D. L., Reaction Scope of Methyl 1,2,3-Triazine-5-carboxylate with Amidines and the Impact of C4/C6 Substitution. *The Journal of Organic Chemistry* **2021,** *86* (19), 13465-13474.
6. Frisch, M.; Trucks, G.; Schlegel, H.; Scuseria, G.; Robb, M.; Cheeseman, J.; Scalmani, G.; Barone, V.; Mennucci, B.; Petersson, G., Gaussian 09 Revision D. 01, 2009. *Gaussian Inc. Wallingford CT* **2009**.
7. Zhao, Y.; Truhlar, D. G., The M06 suite of density functionals for main group thermochemistry, thermochemical kinetics, noncovalent interactions, excited states, and transition elements: two new functionals and systematic testing of four M06-class functionals and 12 other functionals. *Theor. Chem. Acc.* **2007,** *120* (1-3), 215-241.
8. Grimme, S.; Antony, J.; Ehrlich, S.; Krieg, H., A consistent and accurate ab initio parametrization of density functional dispersion correction (DFT-D) for the 94 elements H-Pu. *J. Chem. Phys.* **2010,** *132* (15), 154104.
9. Goerigk, L.; Hansen, A.; Bauer, C.; Ehrlich, S.; Najibi, A.; Grimme, S., A look at the density functional theory zoo with the advanced GMTKN55 database for general main group thermochemistry, kinetics and noncovalent interactions. *Physical Chemistry Chemical Physics* **2017,** *19* (48), 32184-32215.
10. Paton, R. GoodVibes (10.5281/zenodo.841362).
11. Bannwarth, C.; Ehlert, S.; Grimme, S., GFN2-xTB-An Accurate and Broadly Parametrized Self-Consistent Tight-Binding Quantum Chemical Method with Multipole Electrostatics and Density-Dependent Dispersion Contributions. *Journal of Chemical Theory and Computation* **2019,** *15* (3), 1652-1671.
12. Pracht, P.; Bohle, F.; Grimme, S., Automated exploration of the low-energy chemical space with fast quantum chemical methods. *Physical Chemistry Chemical Physics* **2020,** *22* (14), 7169-7192.
13. CYLview, 1.0b; Legault, C. Y., Université de Sherbrooke, 2009 (http://www.cylview.org).
14. Skodje, R. T.; Truhlar, D. G.; Garrett, B. C., A general small-curvature approximation for transition-state-theory transmission coefficients. *The Journal of Physical Chemistry* **2002,** *85* (21), 3019-3023.

VI. Spectra

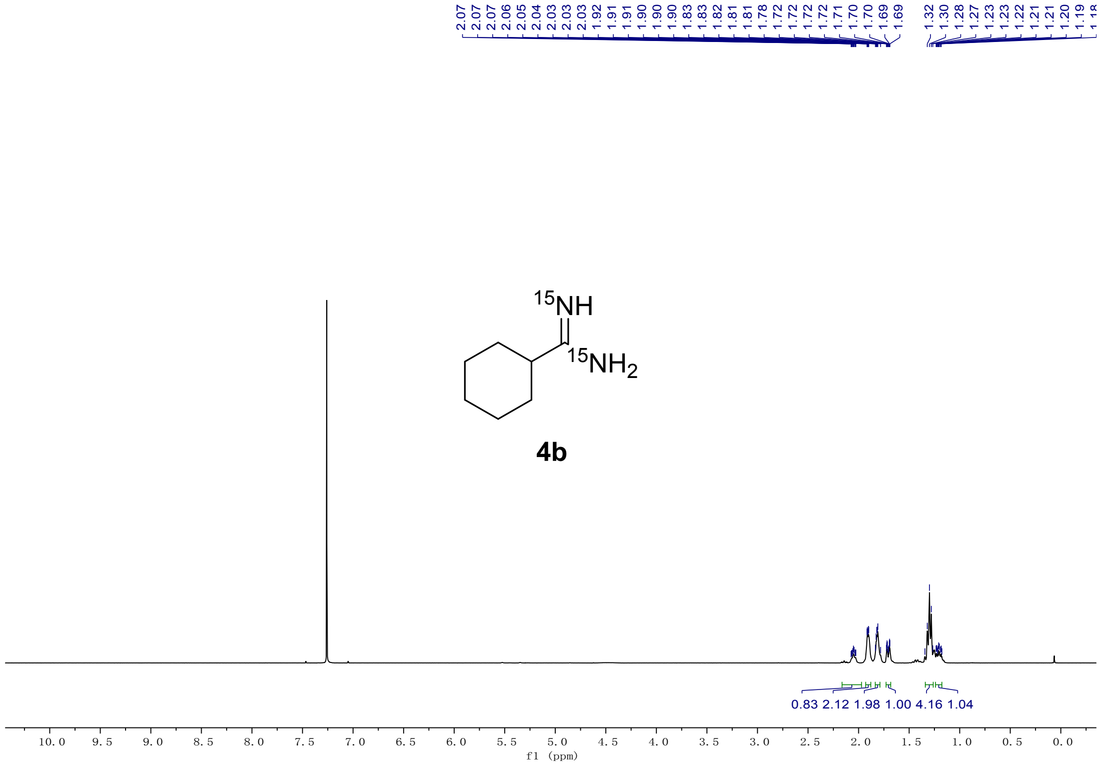
2.07 2.07 2.06 2.05 2.04 2.03 2.03 2.03 1.92 1.91 1.91 1.90 1.90 1.90 1.83 1.83 1.82 1.81 1.81 1.78 1.72 1.72 1.72 1.72 1.72 1.71 1.70 1.70 1.69 1.69
1.32 1.30 1.28 1.27 1.23 1.23 1.22 1.21 1.21 1.20 1.19
15NH
15NH2
4b
0.83 2.12 1.98 1.00 4.16 1.04
f1 (ppm)

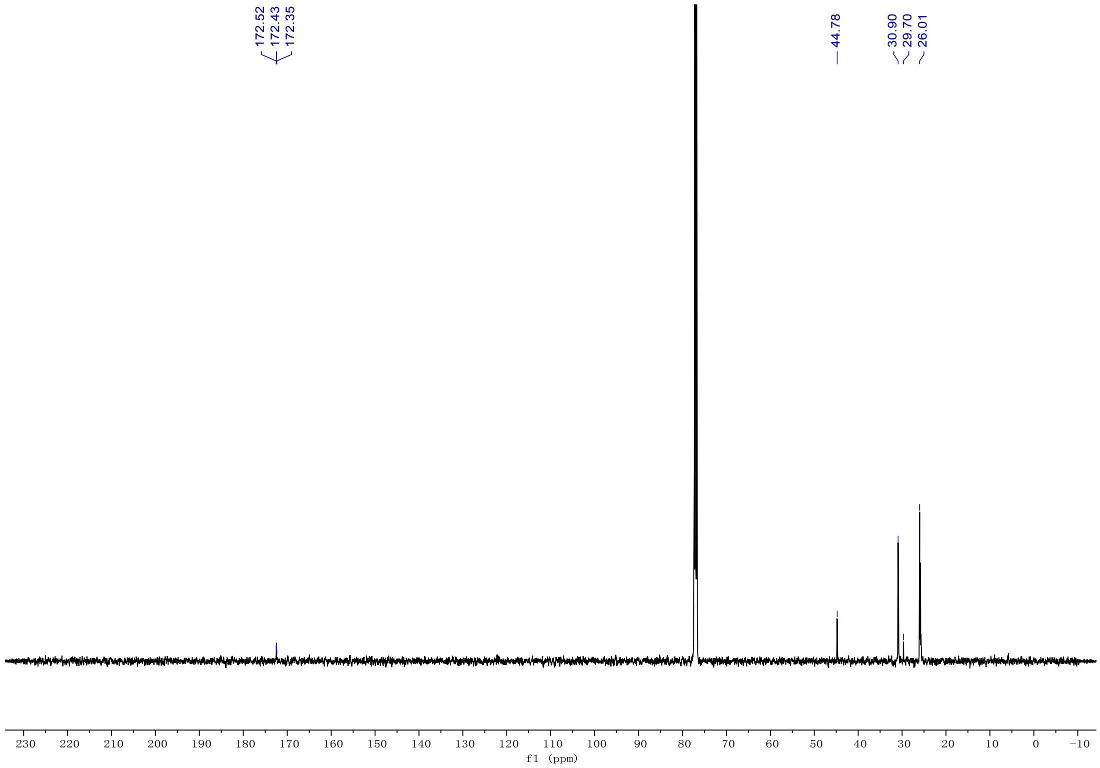
172.52 172.43 172.35
44.78
30.90 29.70 26.01
f1 (ppm)

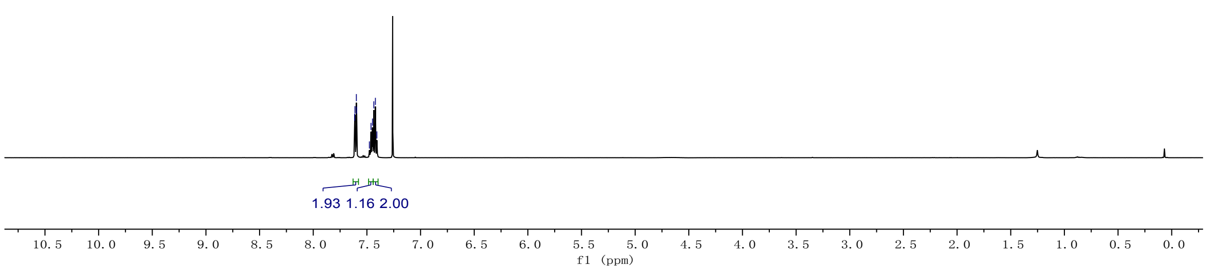
7.61
7.61
7.60
7.59
7.48
7.46
7.45
7.44
7.42
7.41
1.93 1.16 2.00
10.5 10.0 9.5 9.0 8.5 8.0 7.5 7.0 6.5 6.0 5.5 5.0 4.5 4.0 3.5 3.0 2.5 2.0 1.5 1.0 0.5 0.0
f1 (ppm)

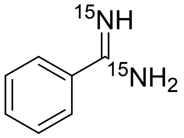
15NH
15NH2

4c

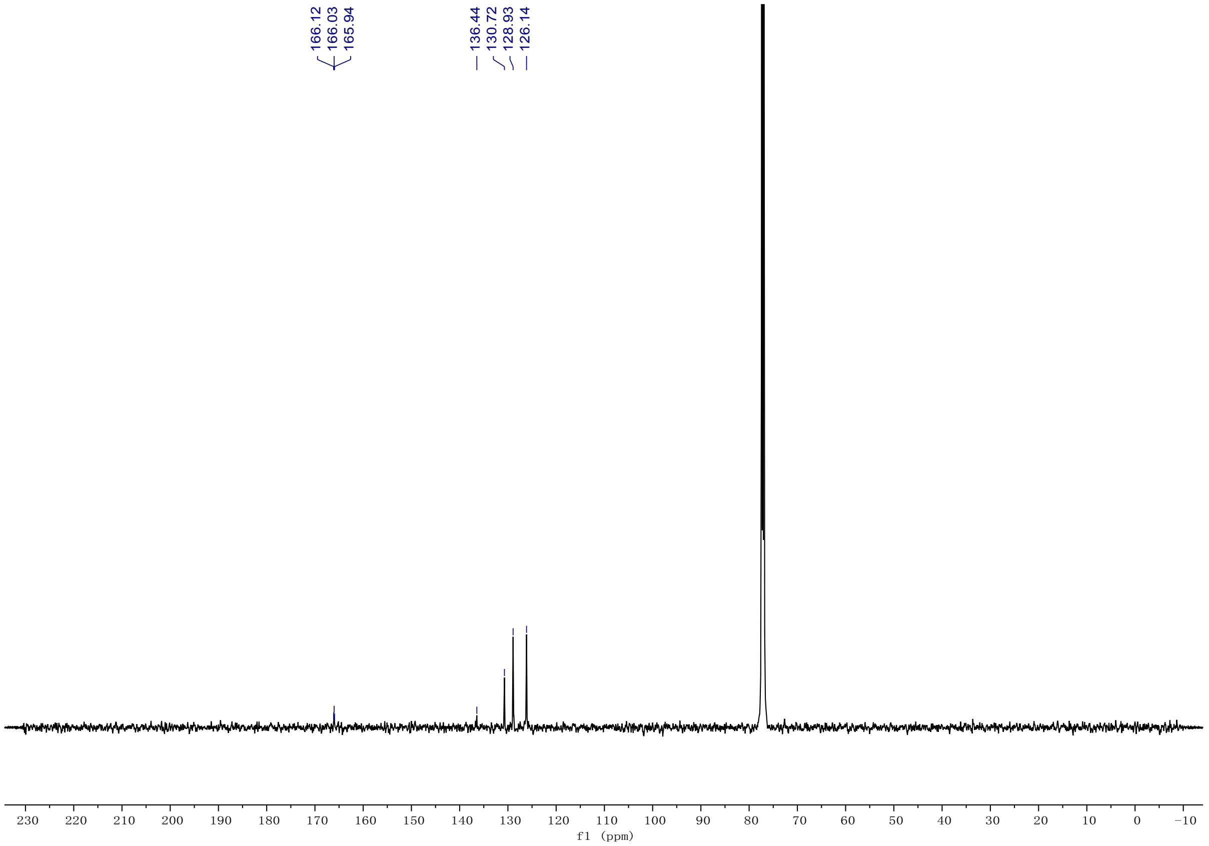
166.12
166.03
165.94
136.44
130.72
128.93
126.14
230 220 210 200 190 180 170 160 150 140 130 120 110 100 90 80 70 60 50 40 30 20 10 0 -10
f1 (ppm)

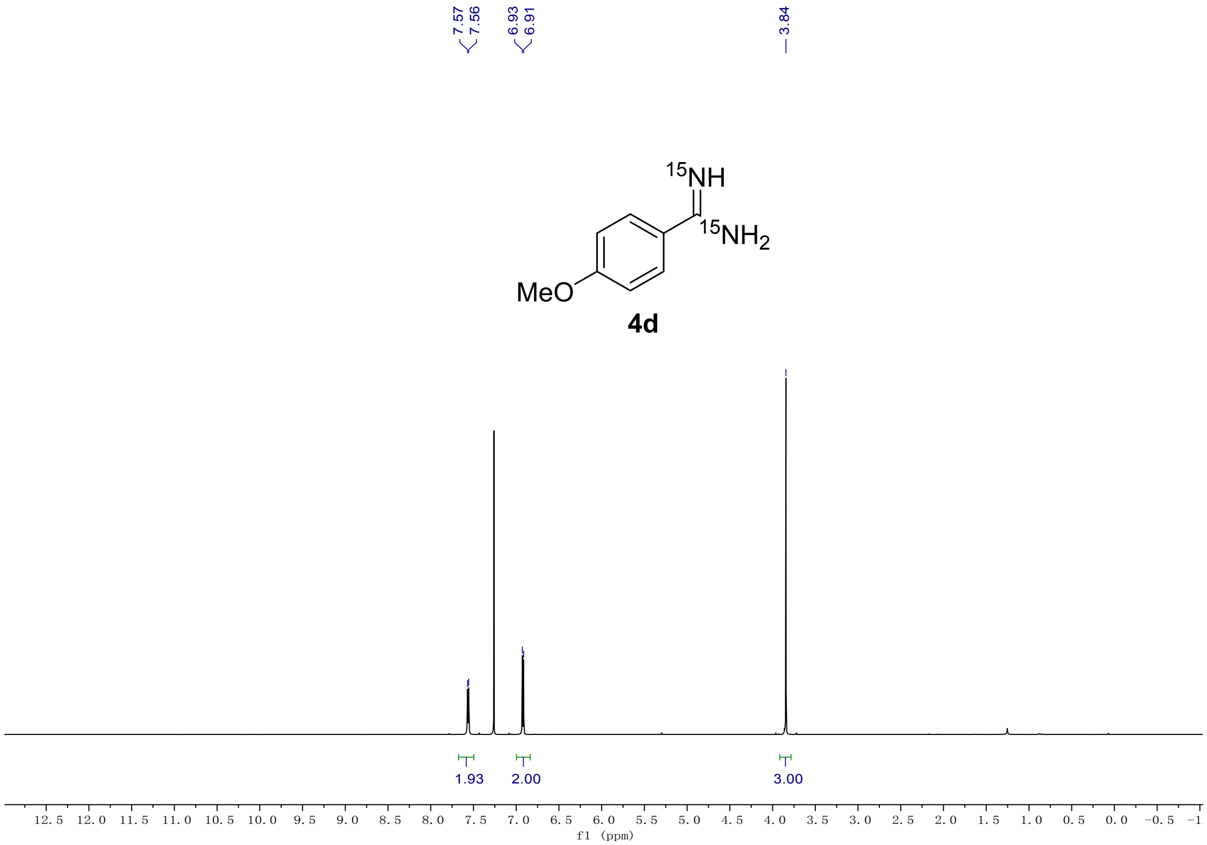
7.57
7.56
6.93
6.91
3.84
15NH
15NH2
MeO
4d
1.93
2.00
3.00
12.5 12.0 11.5 11.0 10.5 10.0 9.5 9.0 8.5 8.0 7.5 7.0 6.5 6.0 5.5 5.0 4.5 4.0 3.5 3.0 2.5 2.0 1.5 1.0 0.5 0.0 −0.5 −1
f1 (ppm)

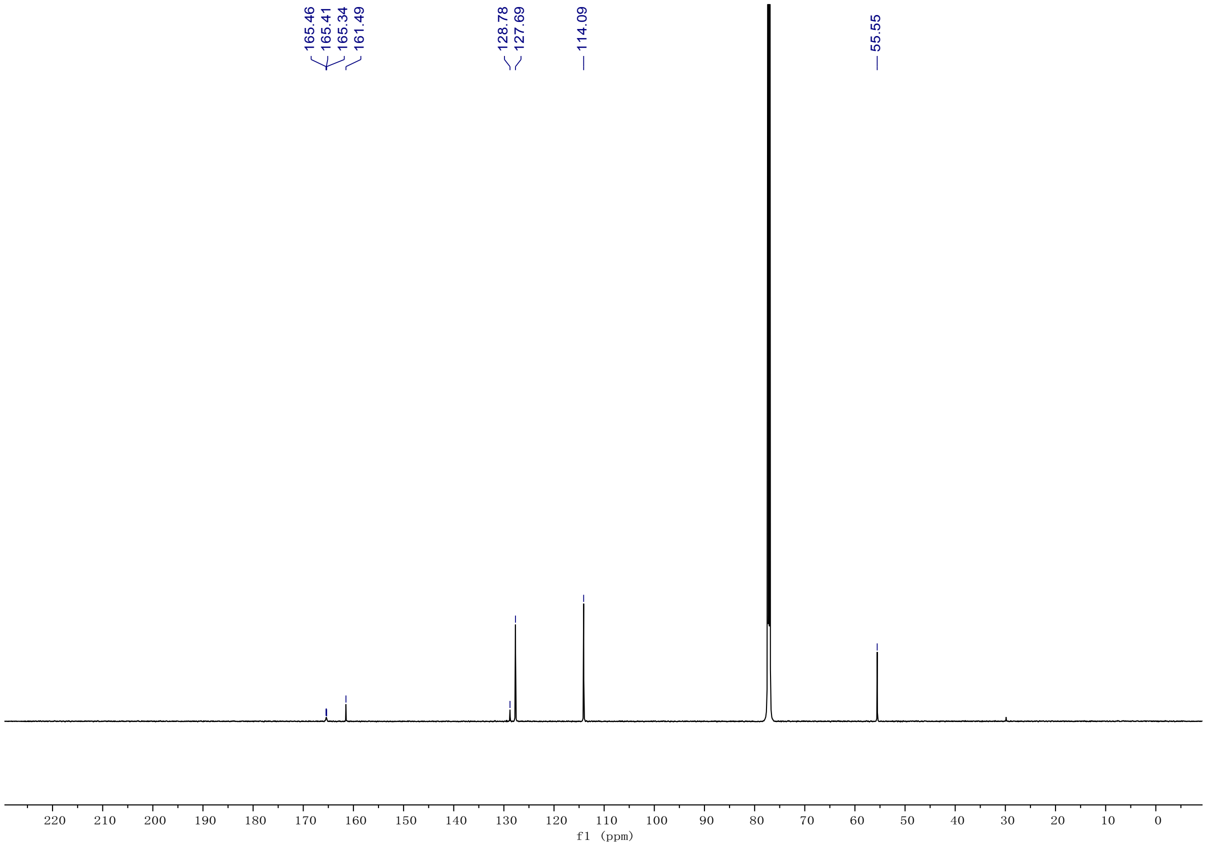
165.46
165.41
165.34
161.49
128.78
127.69
114.09
55.55
220 210 200 190 180 170 160 150 140 130 120 110 100 90 80 70 60 50 40 30 20 10 0
f1 (ppm)

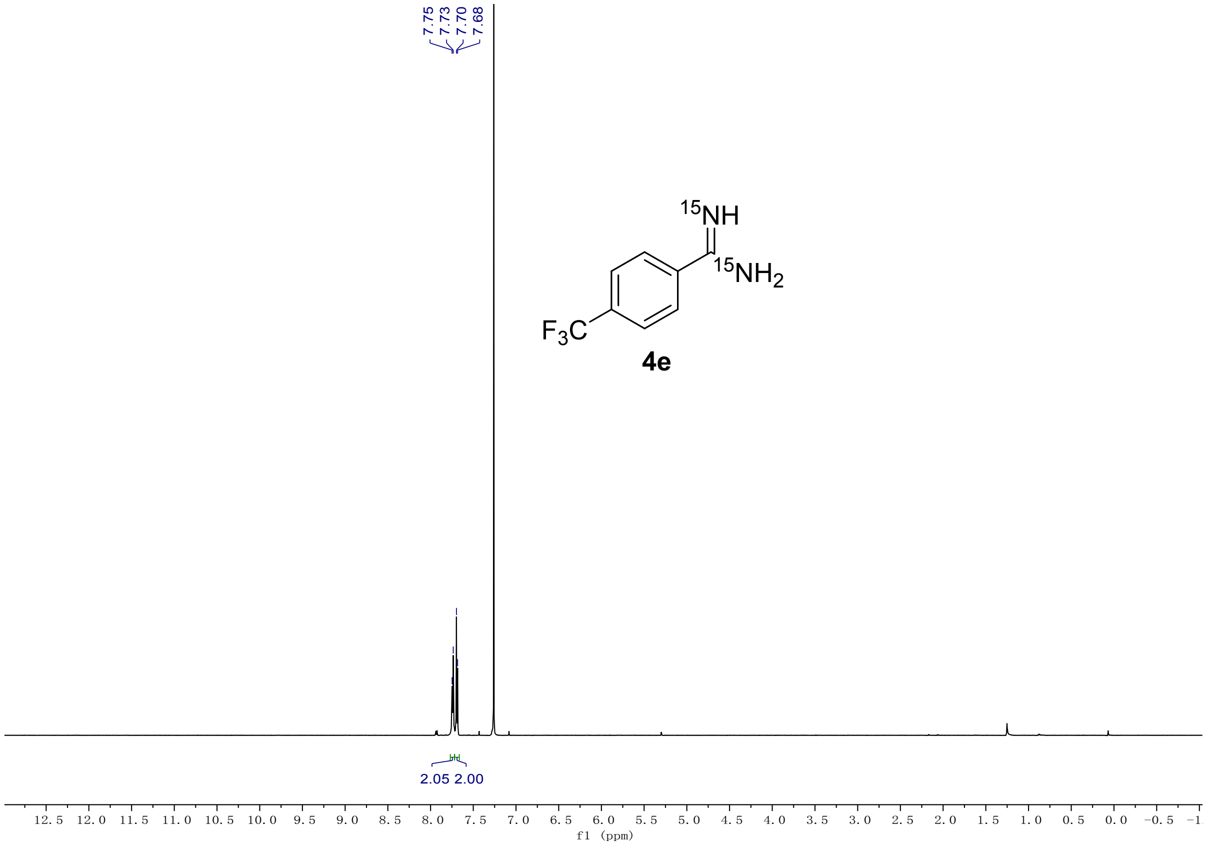
7.75
7.73
7.70
7.68
15NH
15NH2
F3C
4e
2.05 2.00
12.5 12.0 11.5 11.0 10.5 10.0 9.5 9.0 8.5 8.0 7.5 7.0 6.5 6.0 5.5 5.0 4.5 4.0 3.5 3.0 2.5 2.0 1.5 1.0 0.5 0.0 -0.5 -1
f1 (ppm)

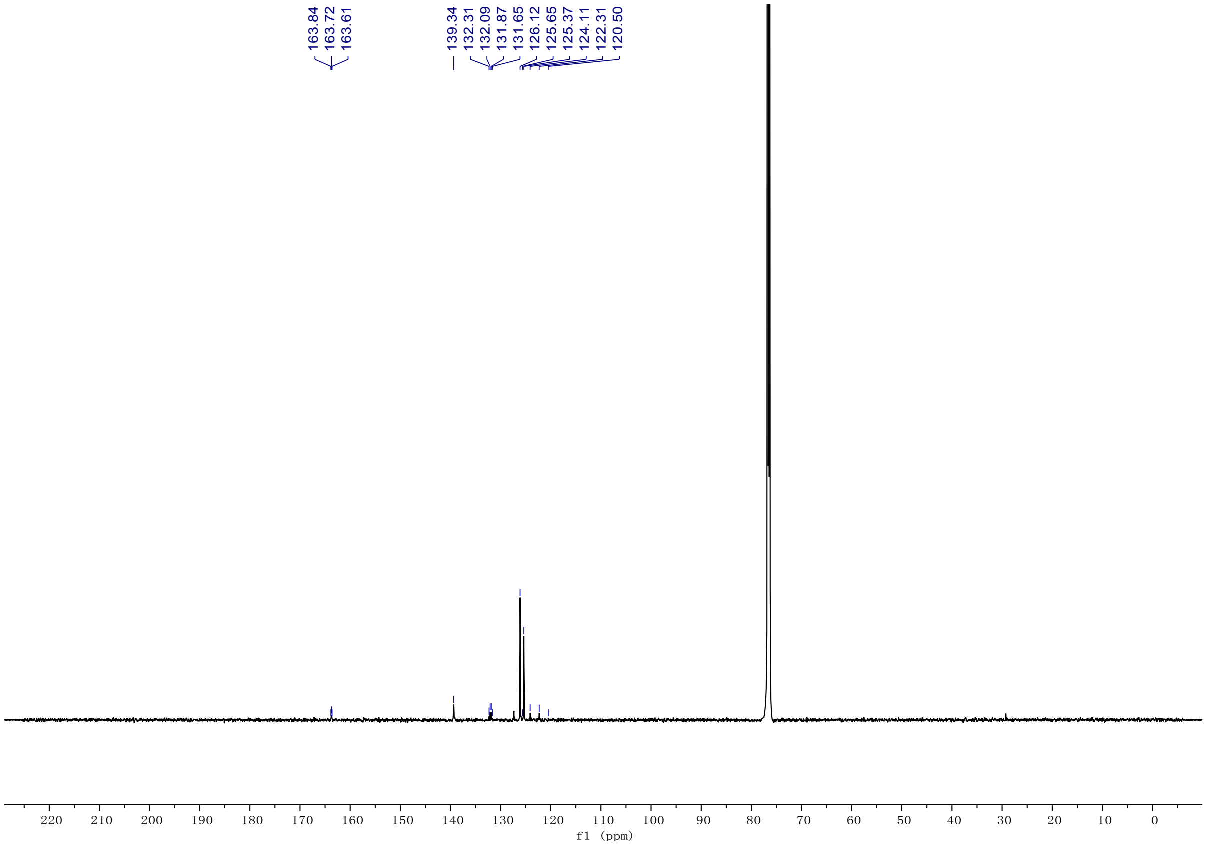
163.84
163.72
163.61
139.34
132.31
132.09
131.87
131.65
126.12
125.65
125.37
124.11
122.31
120.50
220 210 200 190 180 170 160 150 140 130 120 110 100 90 80 70 60 50 40 30 20 10 0
f1 (ppm)

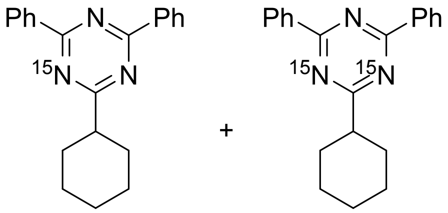
Ph
N
Ph
^{15}N
N
+
Ph
N
Ph
^{15}N
^{15}N

92%   8%

5b

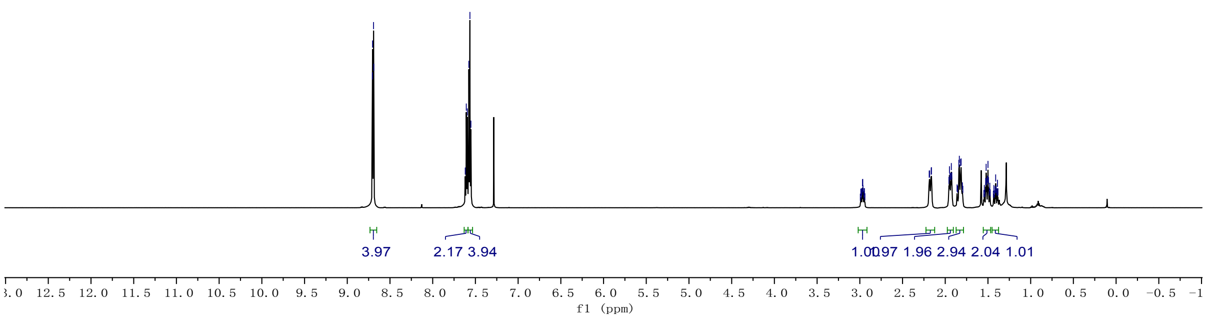
8.71
8.70
8.69
7.62
7.61
7.59
7.58
7.56
7.55
2.99
2.98
2.98
2.97
2.97
2.96
2.95
2.95
2.94
2.19
2.18
2.16
1.96
1.95
1.93
1.93
1.92
1.86
1.84
1.84
1.82
1.81
1.80
1.79
1.55
1.54
1.54
1.53
1.52
1.52
1.51
1.50
1.49
1.48
1.48
1.47
1.44
1.43
1.42
1.41
1.40
1.39
1.38
3.97
2.17 3.94
1.00
0.97 1.96 2.94 2.04 1.01
f1 (ppm)

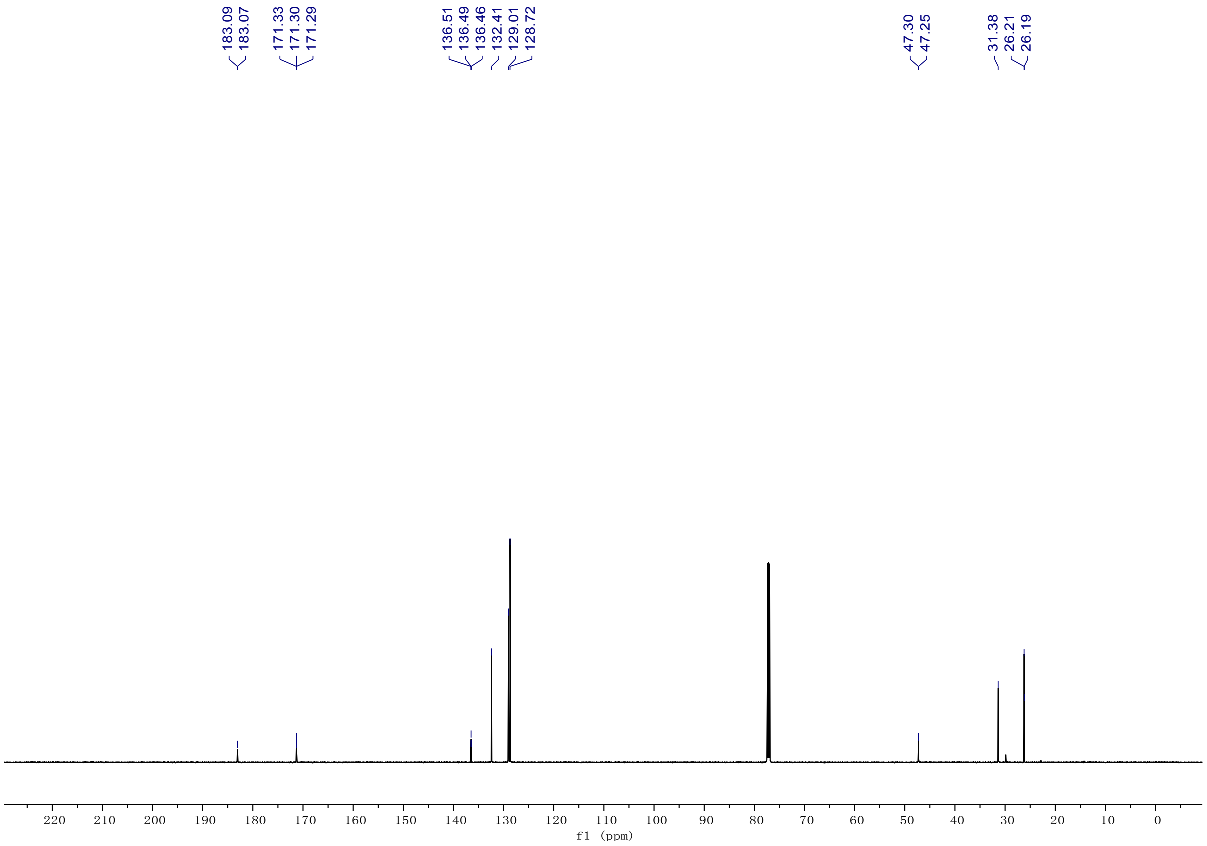
183.09
183.07
171.33
171.30
171.29
136.51
136.49
136.46
132.41
129.01
128.72
47.30
47.25
31.38
26.21
26.19
f1 (ppm)

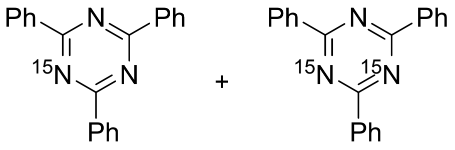
Ph
N
Ph
15N
N
Ph
+
Ph
N
Ph
15N
15N
Ph

49% 51%

5c

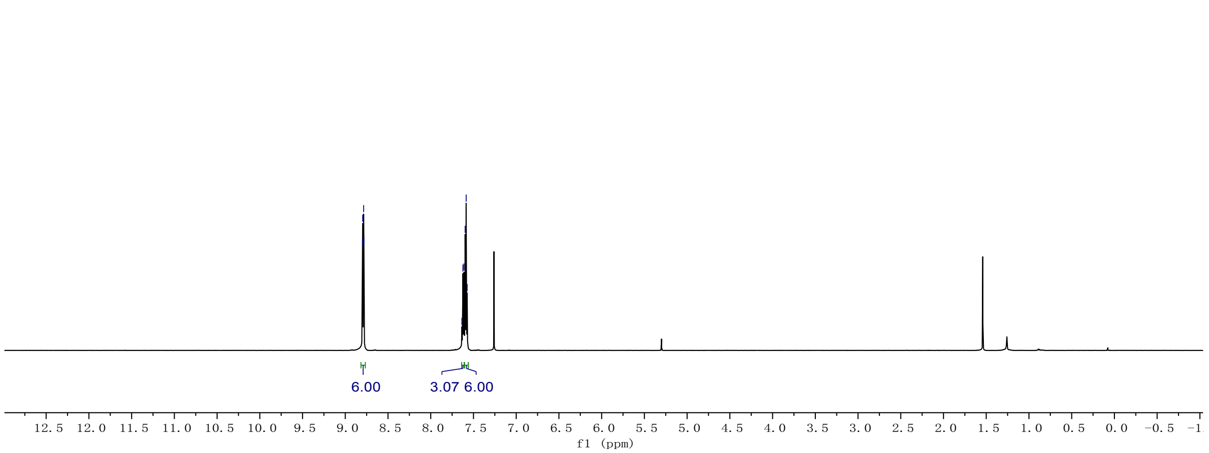
8.80
8.80
8.78
8.78
7.64
7.62
7.61
7.60
7.59
7.57
6.00
3.07 6.00
12.5 12.0 11.5 11.0 10.5 10.0 9.5 9.0 8.5 8.0 7.5 7.0 6.5 6.0 5.5 5.0 4.5 4.0 3.5 3.0 2.5 2.0 1.5 1.0 0.5 0.0 -0.5 -1
f1 (ppm)

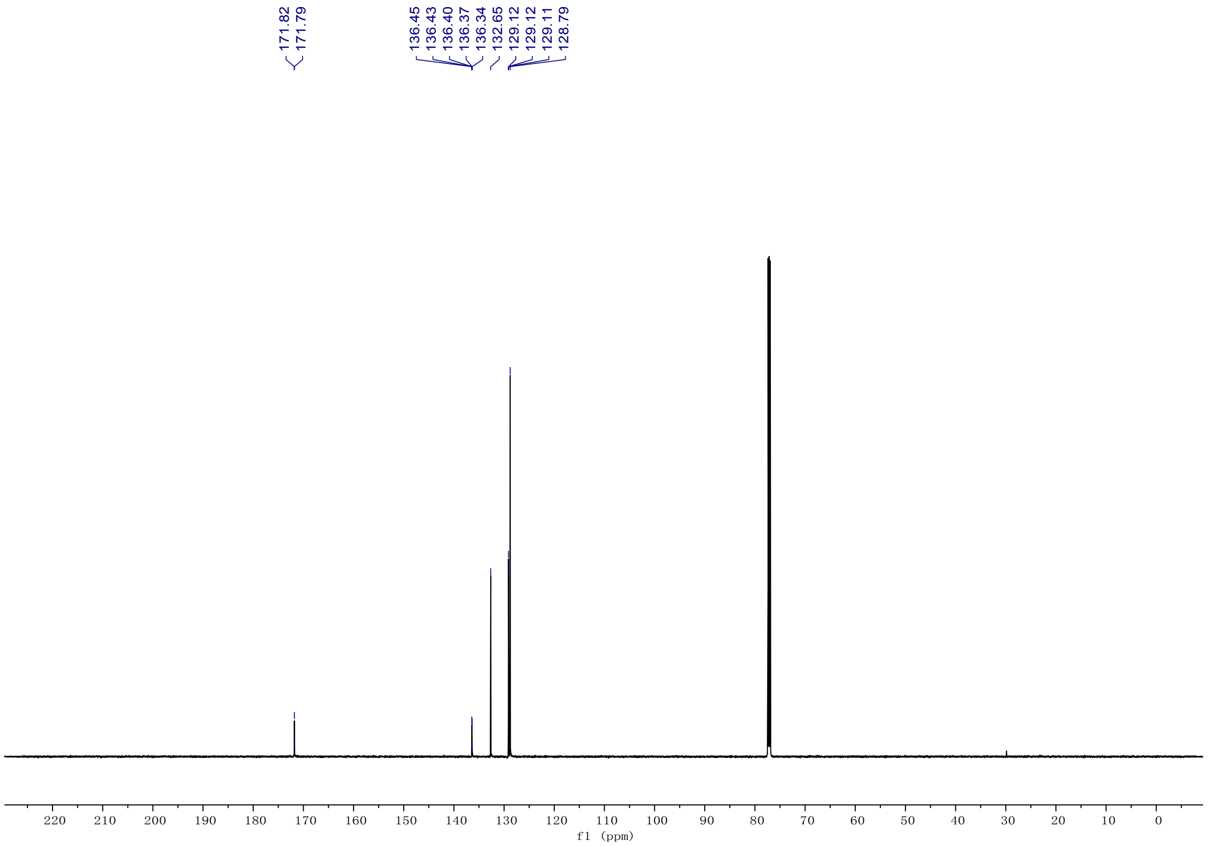
171.82
171.79
136.45
136.43
136.40
136.37
136.34
132.65
129.12
129.12
129.11
128.79
220 210 200 190 180 170 160 150 140 130 120 110 100 90 80 70 60 50 40 30 20 10 0
f1 (ppm)

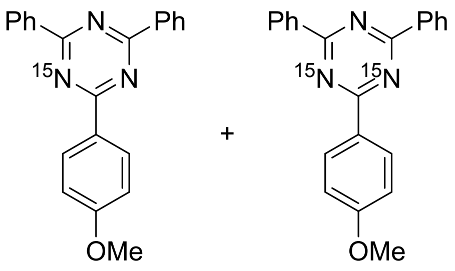
Ph
N
Ph
15N
N
OMe
+
Ph
N
Ph
15N
15N
OMe

51% 49%

5d

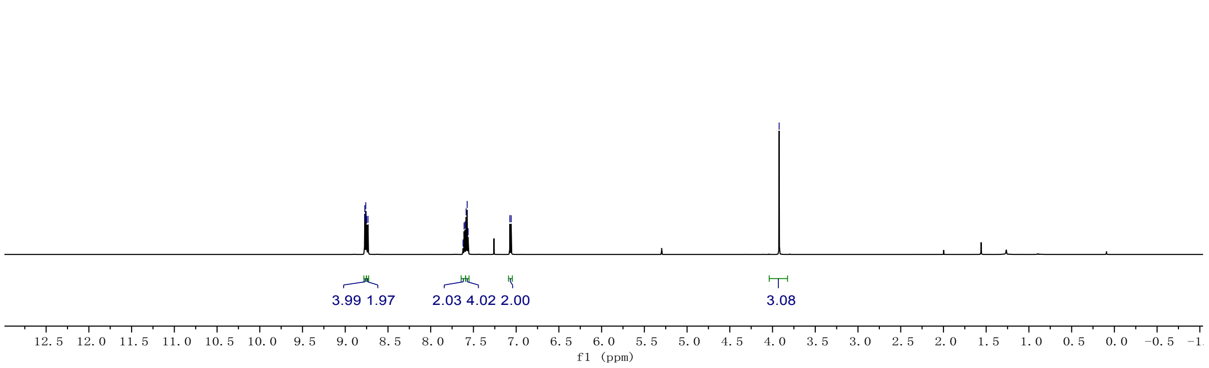
8.77
8.77
8.76
8.76
8.75
8.73
7.62
7.61
7.60
7.59
7.57
7.56
7.07
7.06
3.92
3.99 1.97
2.03 4.02 2.00
3.08
f1 (ppm)

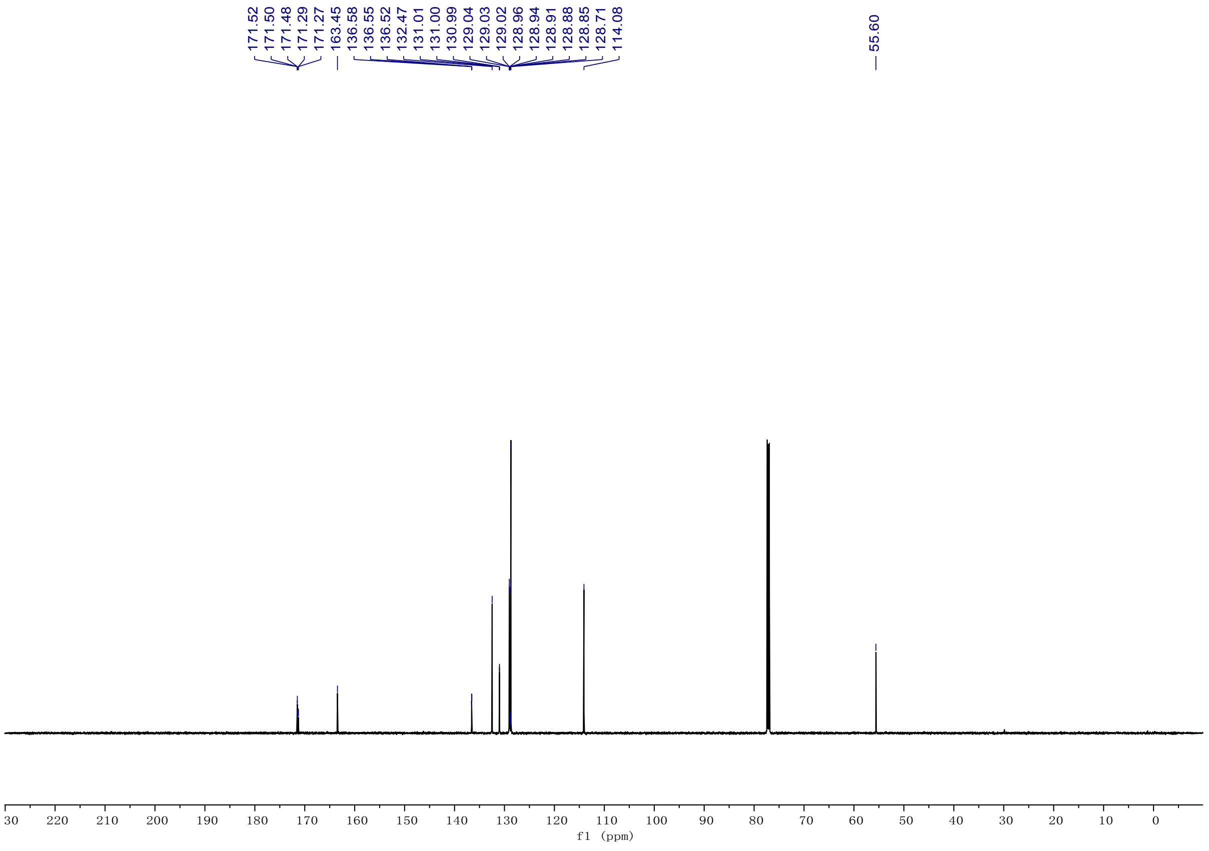
171.52
171.50
171.48
171.29
171.27
163.45
136.58
136.55
136.52
132.47
131.01
131.00
130.99
129.04
129.03
129.02
128.96
128.94
128.91
128.88
128.85
128.71
114.08
55.60
f1 (ppm)

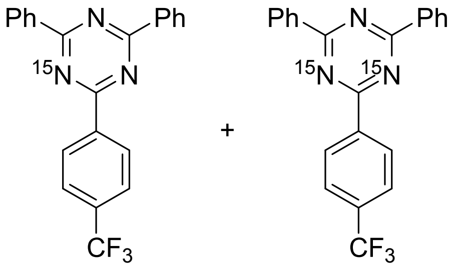
Ph
N
Ph
15N
N
+
Ph
N
Ph
15N
15N
CF3
CF3

56% 44%

5e

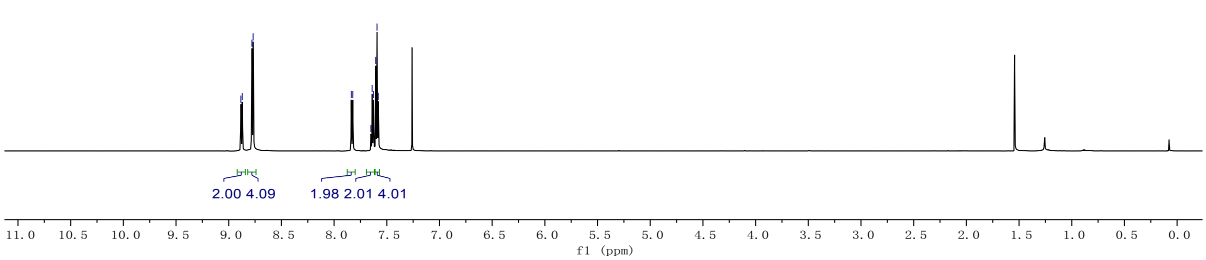
8.88
8.87
8.78
8.77
7.84
7.82
7.65
7.64
7.63
7.61
7.59
7.58
2.00 4.09
1.98 2.01 4.01
11.0 10.5 10.0 9.5 9.0 8.5 8.0 7.5 7.0 6.5 6.0 5.5 5.0 4.5 4.0 3.5 3.0 2.5 2.0 1.5 1.0 0.5 0.0
f1 (ppm)

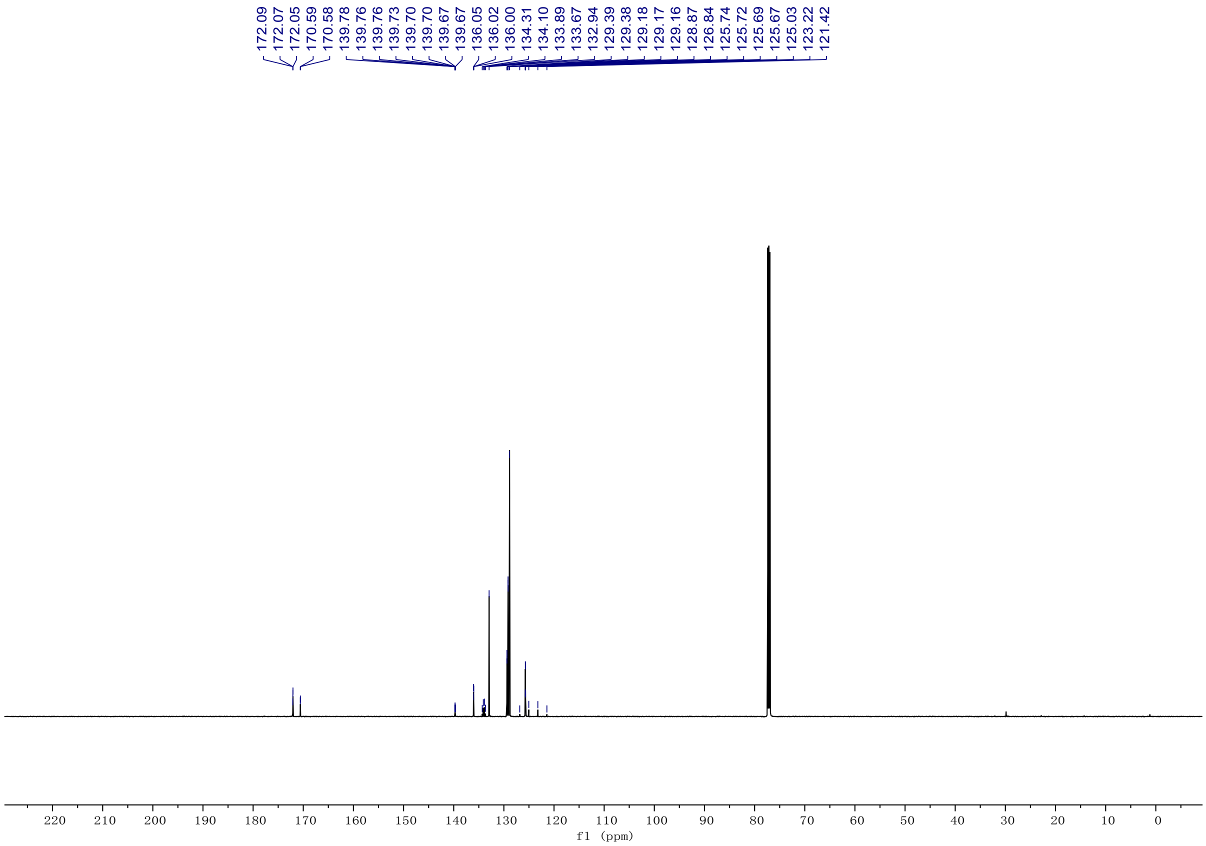
172.09
172.07
172.05
170.59
170.58
139.78
139.76
139.76
139.73
139.70
139.70
139.67
139.67
136.05
136.02
136.00
134.31
134.10
133.89
133.67
132.94
129.39
129.38
129.18
129.17
129.16
128.87
126.84
125.74
125.72
125.69
125.67
125.03
123.22
121.42
220 210 200 190 180 170 160 150 140 130 120 110 100 90 80 70 60 50 40 30 20 10 0
f1 (ppm)

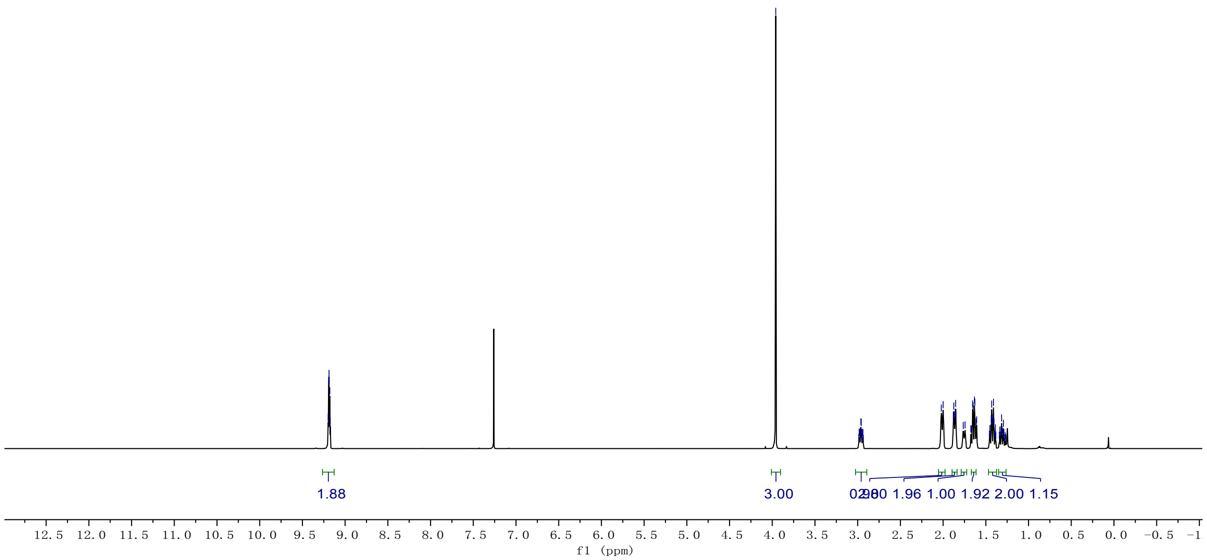

9.20 9.19 9.19 9.19 9.18 9.17 3.96 2.99 2.98 2.98 2.97 2.97 2.96 2.96 2.95 2.95 2.94 2.94 2.93 2.02 2.00 1.88 1.88 1.87 1.86 1.85 1.85 1.76 1.74 1.68 1.67 1.65 1.65 1.63 1.63 1.61 1.61 1.46 1.45 1.45 1.44 1.43 1.43 1.42 1.41 1.40 1.39 1.39 1.38 1.34 1.34 1.33 1.32 1.32 1.31 1.30 1.29 1.28 1.27 1.27
1.88
3.00
1.96 1.00 1.92 2.00 1.15
12.5 12.0 11.5 11.0 10.5 10.0 9.5 9.0 8.5 8.0 7.5 7.0 6.5 6.0 5.5 5.0 4.5 4.0 3.5 3.0 2.5 2.0 1.5 1.0 0.5 0.0 -0.5 -1
f1 (ppm)

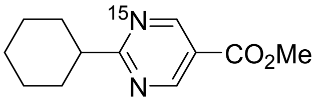

15N
N
CO2Me

6b

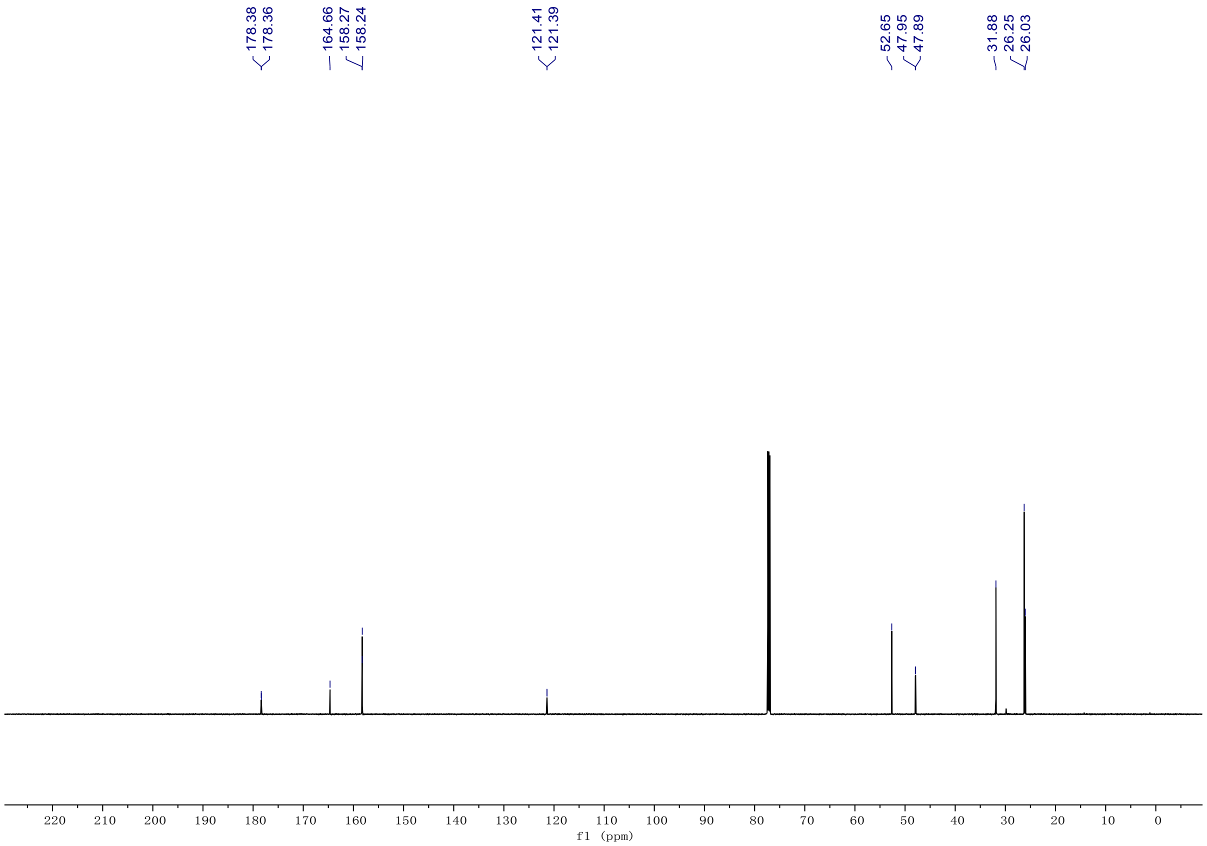

178.38 178.36
164.66 158.27 158.24
121.41 121.39
52.65 47.95 47.89
31.88 26.25 26.03
220 210 200 190 180 170 160 150 140 130 120 110 100 90 80 70 60 50 40 30 20 10 0
f1 (ppm)

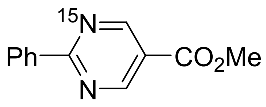
15N
Ph
N
CO2Me

6c

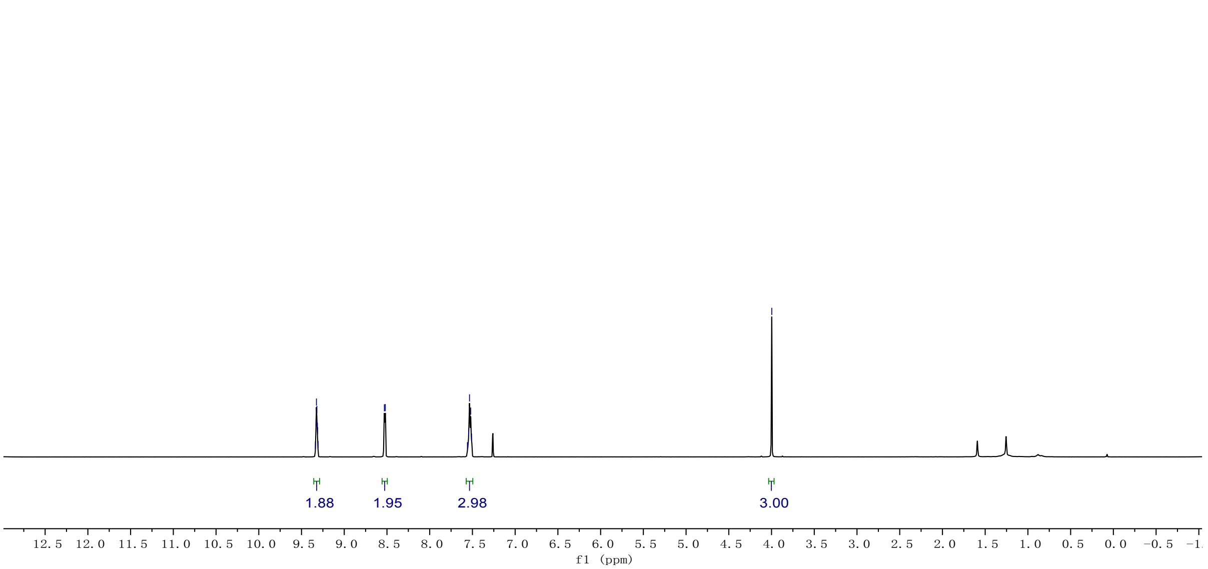
9.34
9.33
9.33
9.32
9.31
9.31
9.31
8.53
8.52
7.56
7.54
7.53
7.52
7.51
4.00
1.88
1.95
2.98
3.00
f1 (ppm)

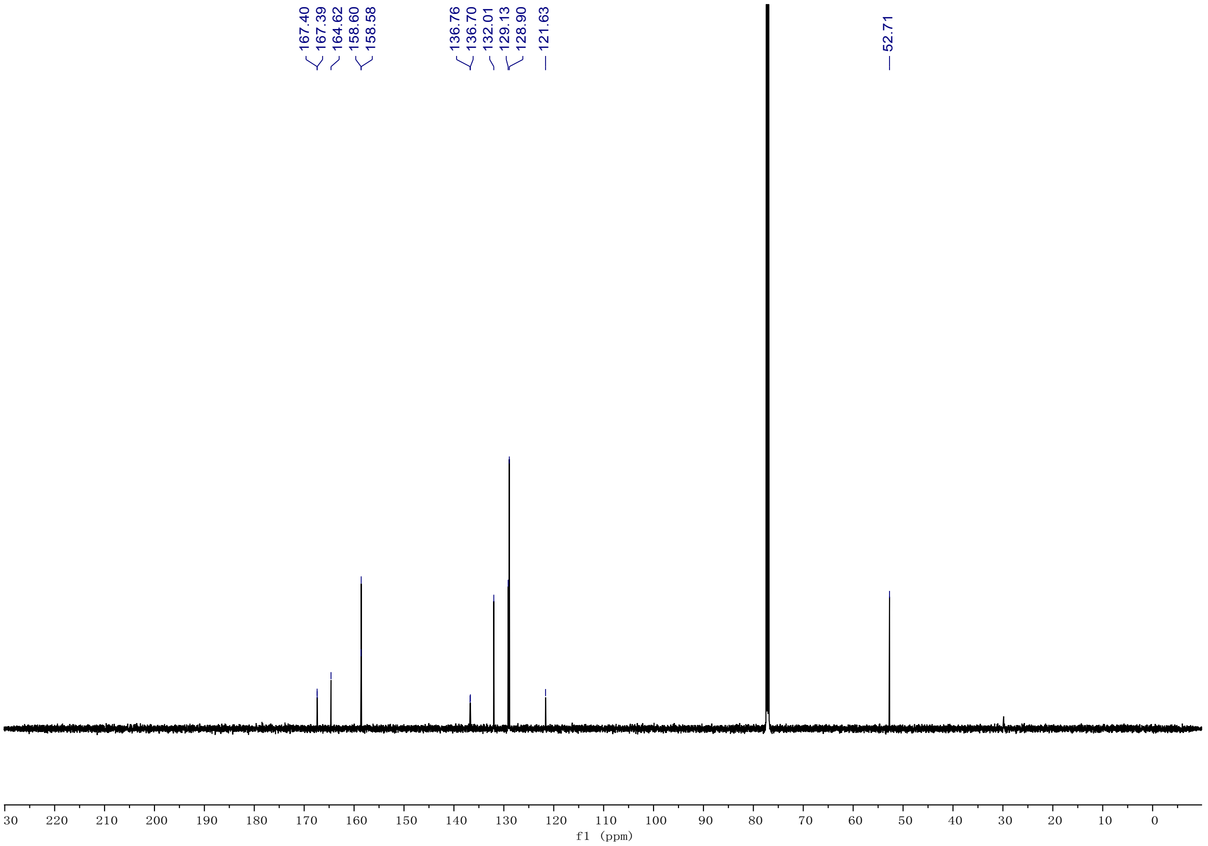
167.40
167.39
164.62
158.60
158.58
136.76
136.70
132.01
129.13
128.90
121.63
52.71
f1 (ppm)

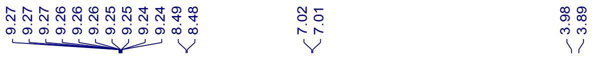
9.27 9.27 9.27 9.26 9.26 9.25 9.25 9.24 9.24 8.49 8.48
7.02 7.01
3.98 3.89

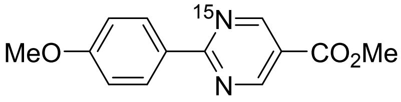
^{15}N
MeO
N
CO_2Me

6d

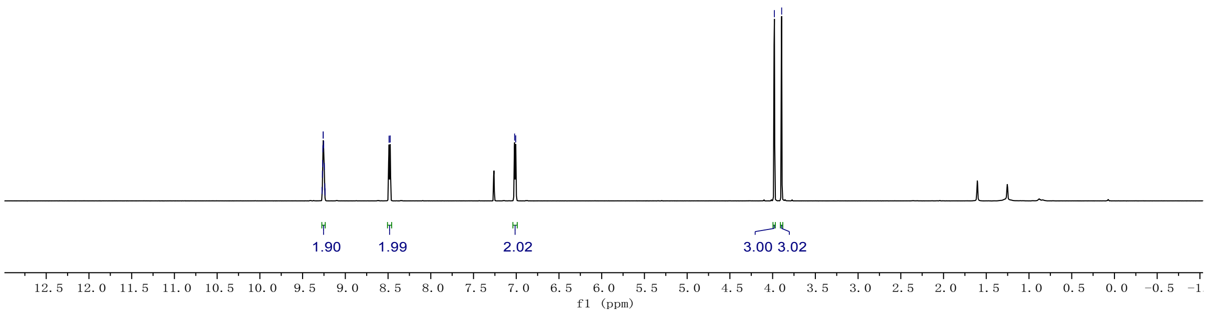
1.90
1.99
2.02
3.00 3.02
f1 (ppm)

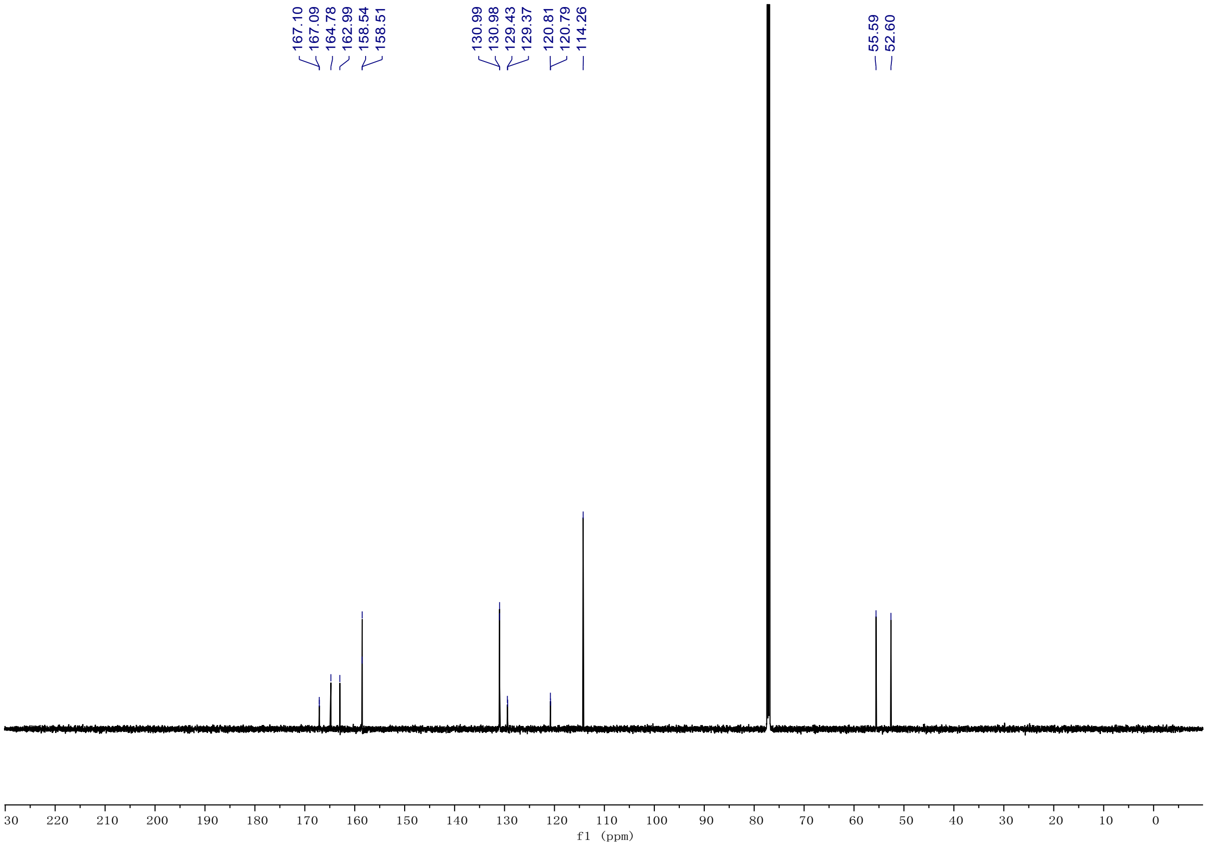
167.10 167.09 164.78 162.99 158.54 158.51
130.99 130.98 129.43 129.37 120.81 120.79 114.26
55.59 52.60
f1 (ppm)

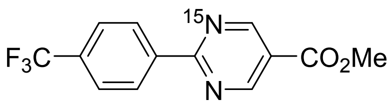
15N
F3C
CO2Me
N

6e

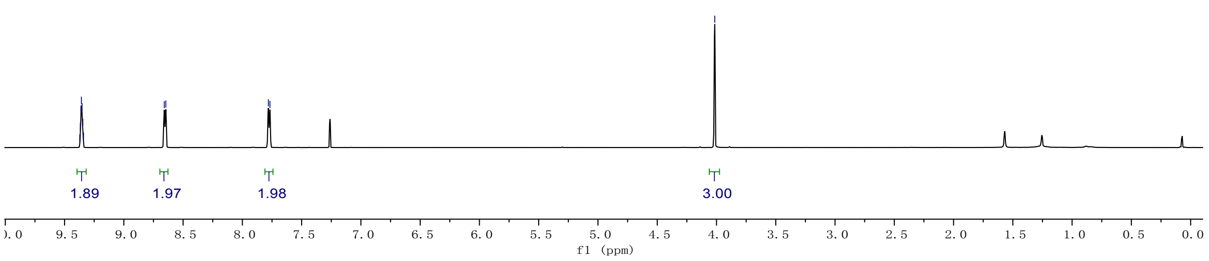
9.37
9.37
9.36
9.36
9.35
9.35
9.34
8.66
8.65
7.78
7.77
4.01
1.89
1.97
1.98
3.00
f1 (ppm)

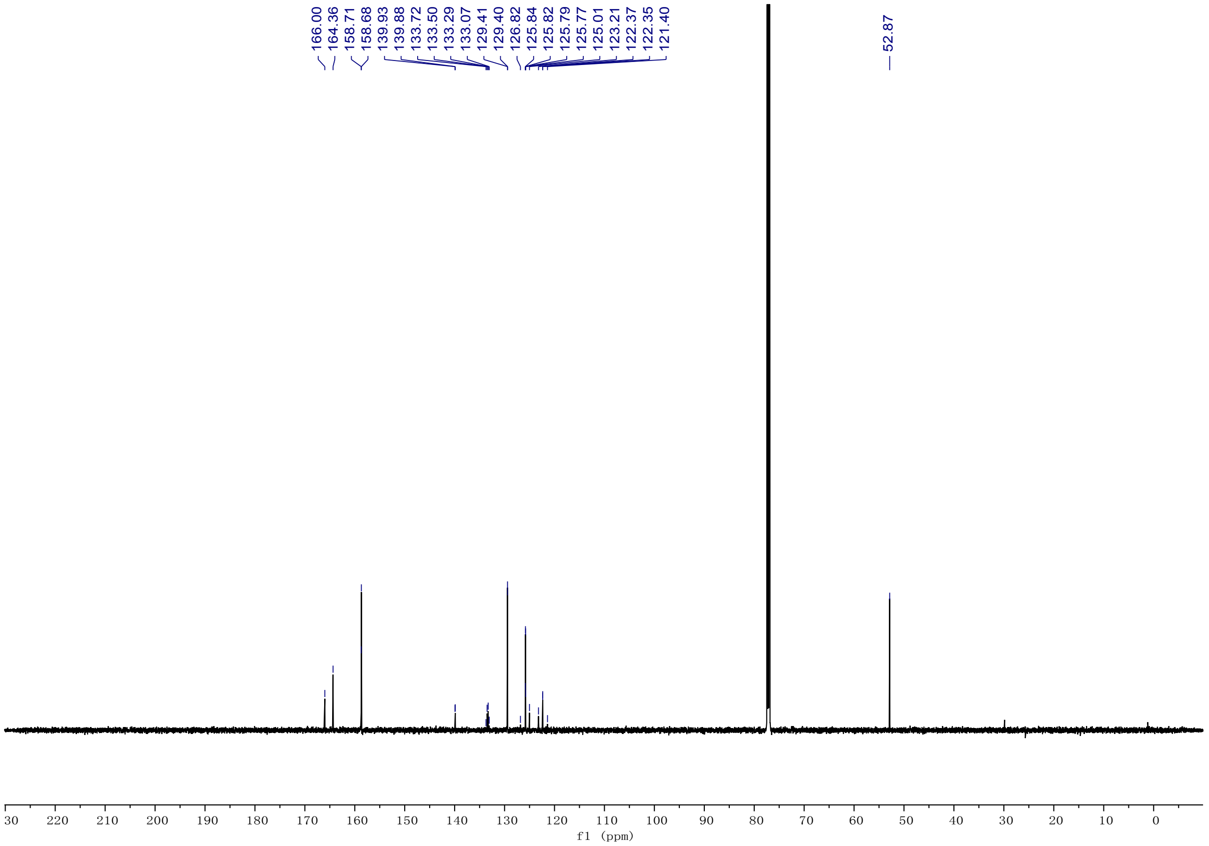
166.00
164.36
158.71
158.68
139.93
139.88
133.72
133.50
133.29
133.07
129.41
129.40
126.82
125.84
125.82
125.79
125.77
125.01
123.21
122.37
122.35
121.40
52.87
f1 (ppm)

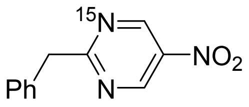
15N
NO2
Ph
N

7a

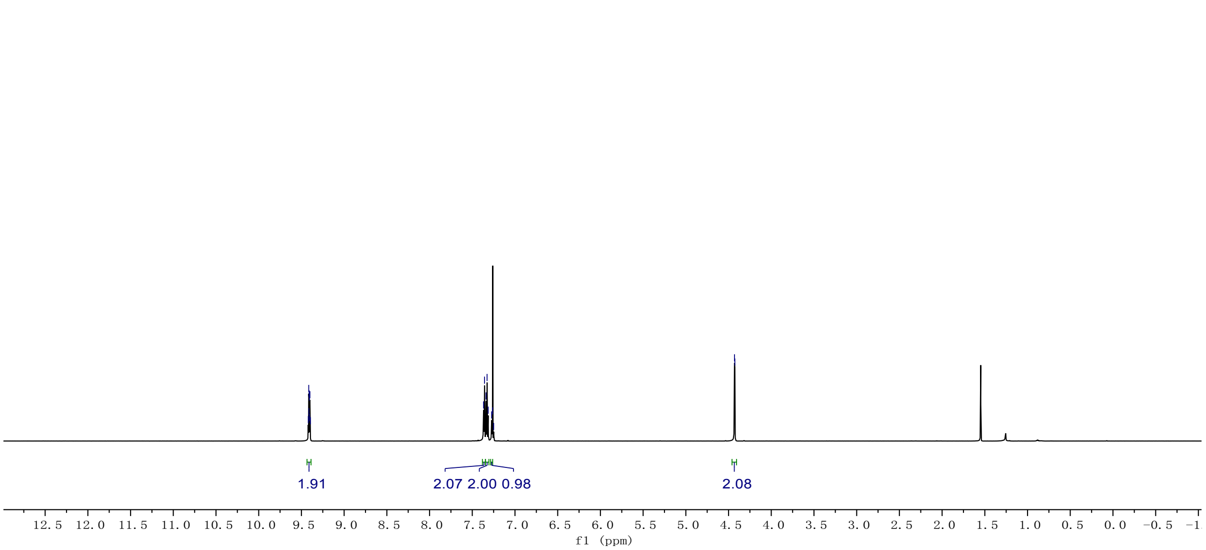
9.42
9.41
9.41
9.41
9.40
9.40
9.40
7.37
7.36
7.34
7.33
7.31
7.27
7.26
7.25
4.43
4.43
1.91
2.07 2.00 0.98
2.08
12.5 12.0 11.5 11.0 10.5 10.0 9.5 9.0 8.5 8.0 7.5 7.0 6.5 6.0 5.5 5.0 4.5 4.0 3.5 3.0 2.5 2.0 1.5 1.0 0.5 0.0 -0.5 -1
f1 (ppm)

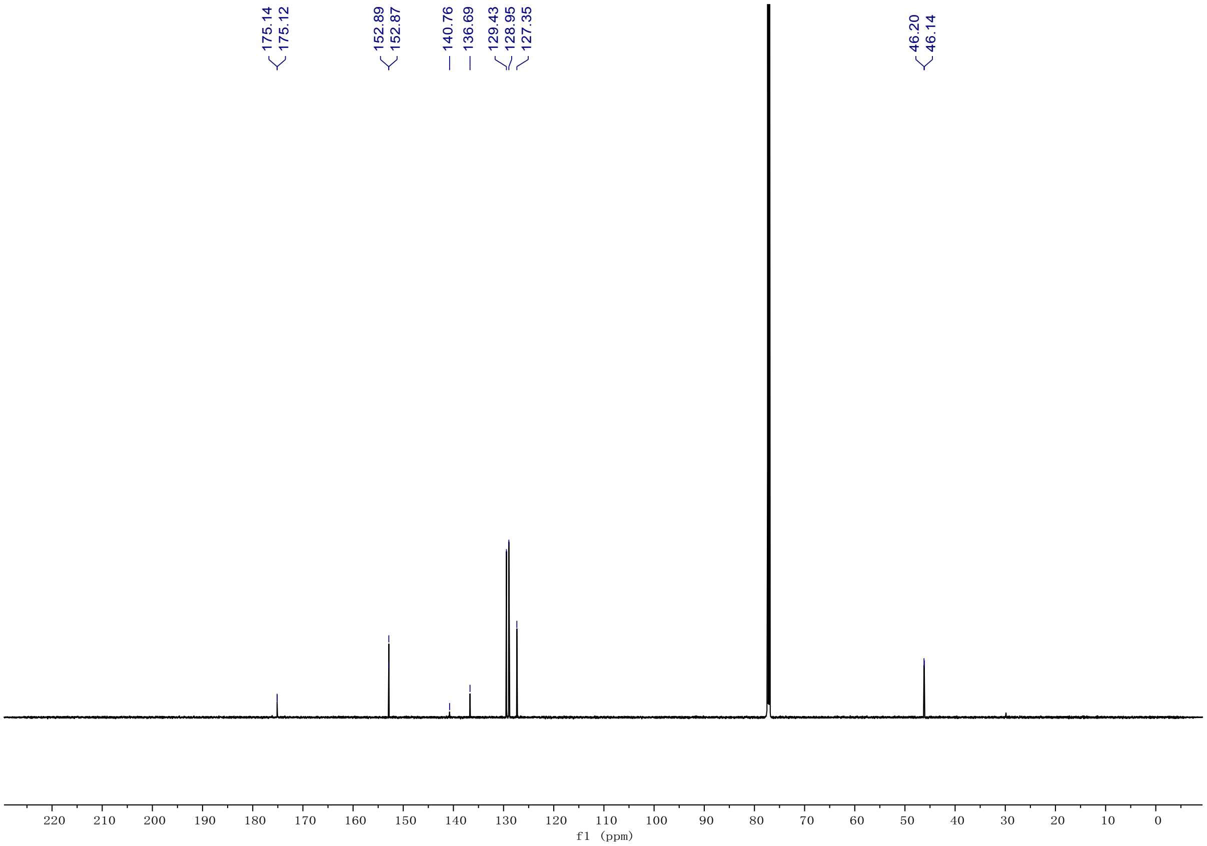
175.14
175.12
152.89
152.87
140.76
136.69
129.43
128.95
127.35
46.20
46.14
220 210 200 190 180 170 160 150 140 130 120 110 100 90 80 70 60 50 40 30 20 10 0
f1 (ppm)

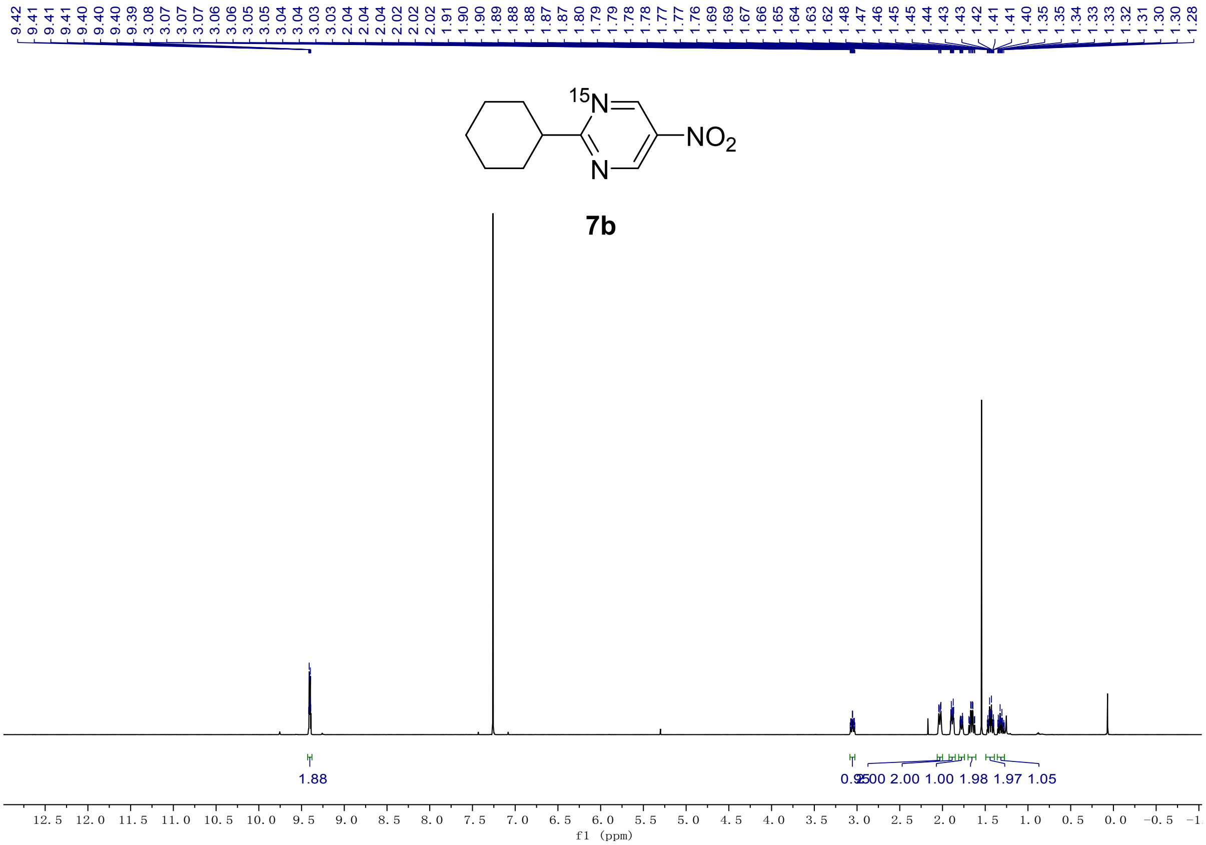
9.42 9.41 9.41 9.40 9.40 9.39 3.08 3.07 3.07 3.06 3.05 3.05 3.04 3.03 3.03 2.04 2.04 2.02 2.02 2.02 1.91 1.90 1.89 1.88 1.88 1.87 1.87 1.80 1.79 1.79 1.78 1.78 1.77 1.76 1.69 1.69 1.67 1.66 1.65 1.64 1.63 1.62 1.48 1.47 1.46 1.45 1.45 1.44 1.43 1.43 1.42 1.41 1.41 1.40 1.35 1.35 1.34 1.33 1.33 1.32 1.31 1.30 1.30 1.28
15N
NO2
7b
1.88
0.95 2.00 2.00 1.00 1.98 1.97 1.05
12.5 12.0 11.5 11.0 10.5 10.0 9.5 9.0 8.5 8.0 7.5 7.0 6.5 6.0 5.5 5.0 4.5 4.0 3.5 3.0 2.5 2.0 1.5 1.0 0.5 0.0 -0.5 -1
f1 (ppm)

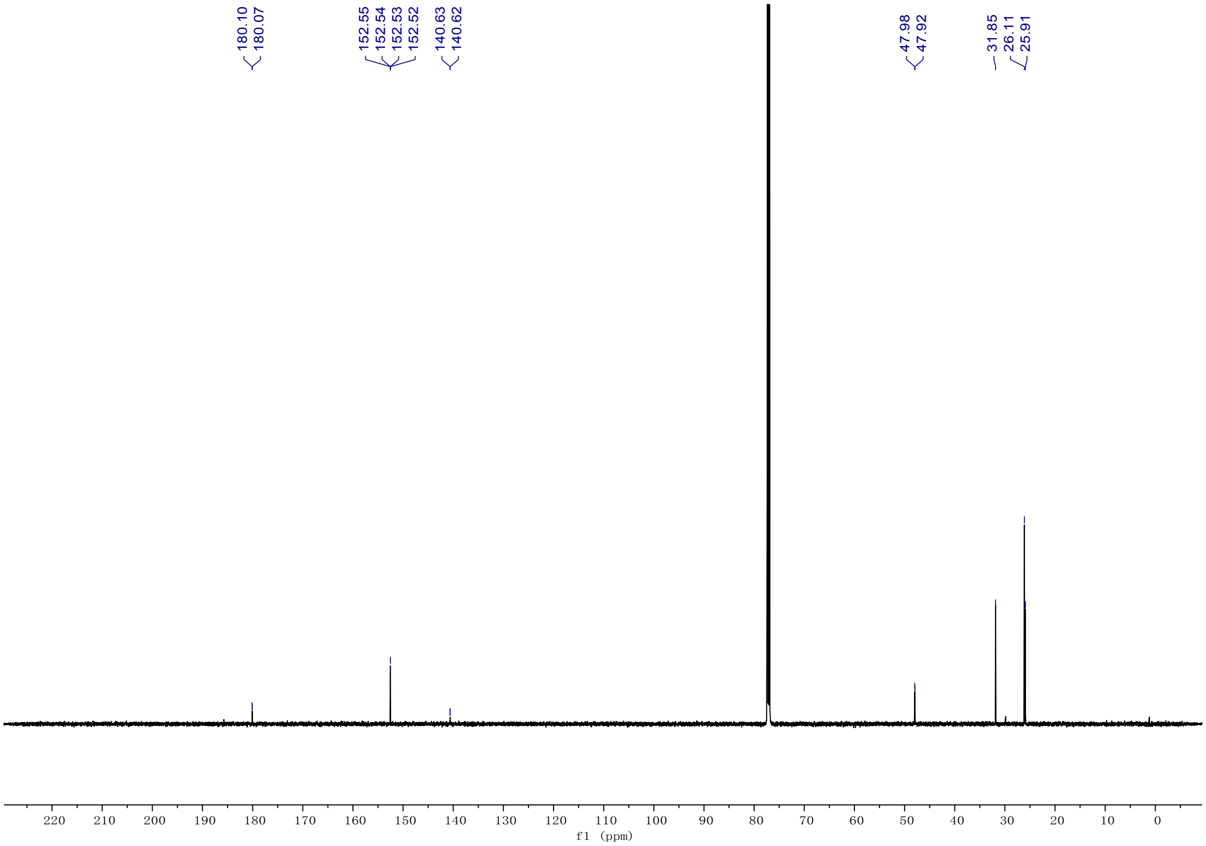
180.10 180.07
152.55 152.54 152.53 152.52
140.63 140.62
47.98 47.92
31.85 26.11 25.91
220 210 200 190 180 170 160 150 140 130 120 110 100 90 80 70 60 50 40 30 20 10 0
f1 (ppm)

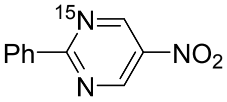
15N
Ph
N
NO2

7c

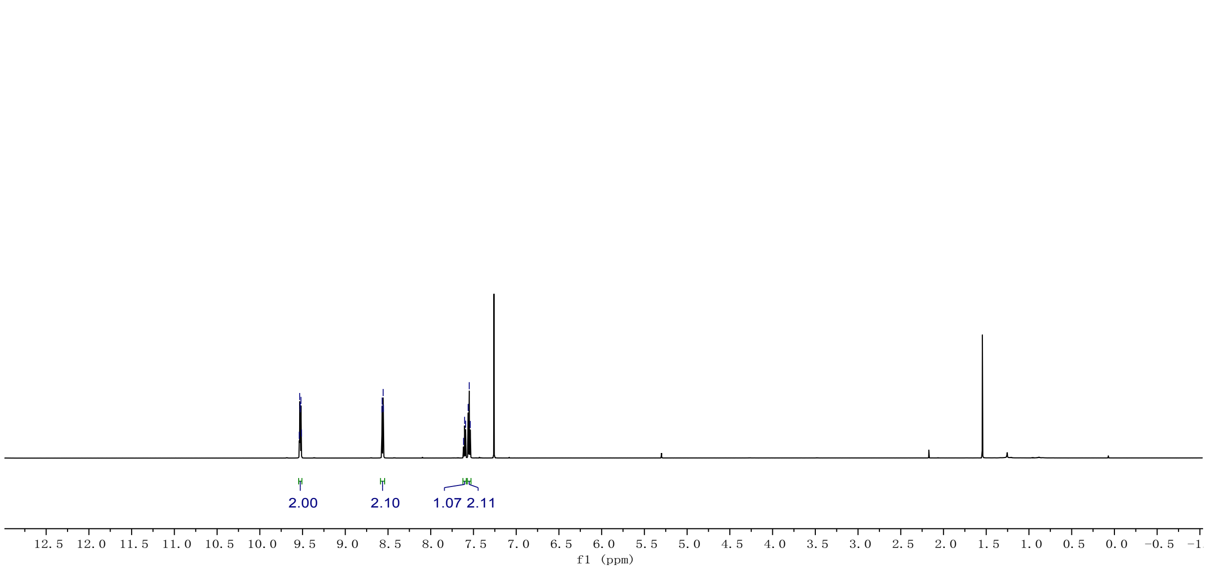
9.54
9.53
9.53
9.53
9.52
9.51
8.57
8.57
8.56
8.55
7.62
7.60
7.59
7.56
7.55
7.54
2.00
2.10
1.07
2.11
12.5 12.0 11.5 11.0 10.5 10.0 9.5 9.0 8.5 8.0 7.5 7.0 6.5 6.0 5.5 5.0 4.5 4.0 3.5 3.0 2.5 2.0 1.5 1.0 0.5 0.0 -0.5 -1
f1 (ppm)

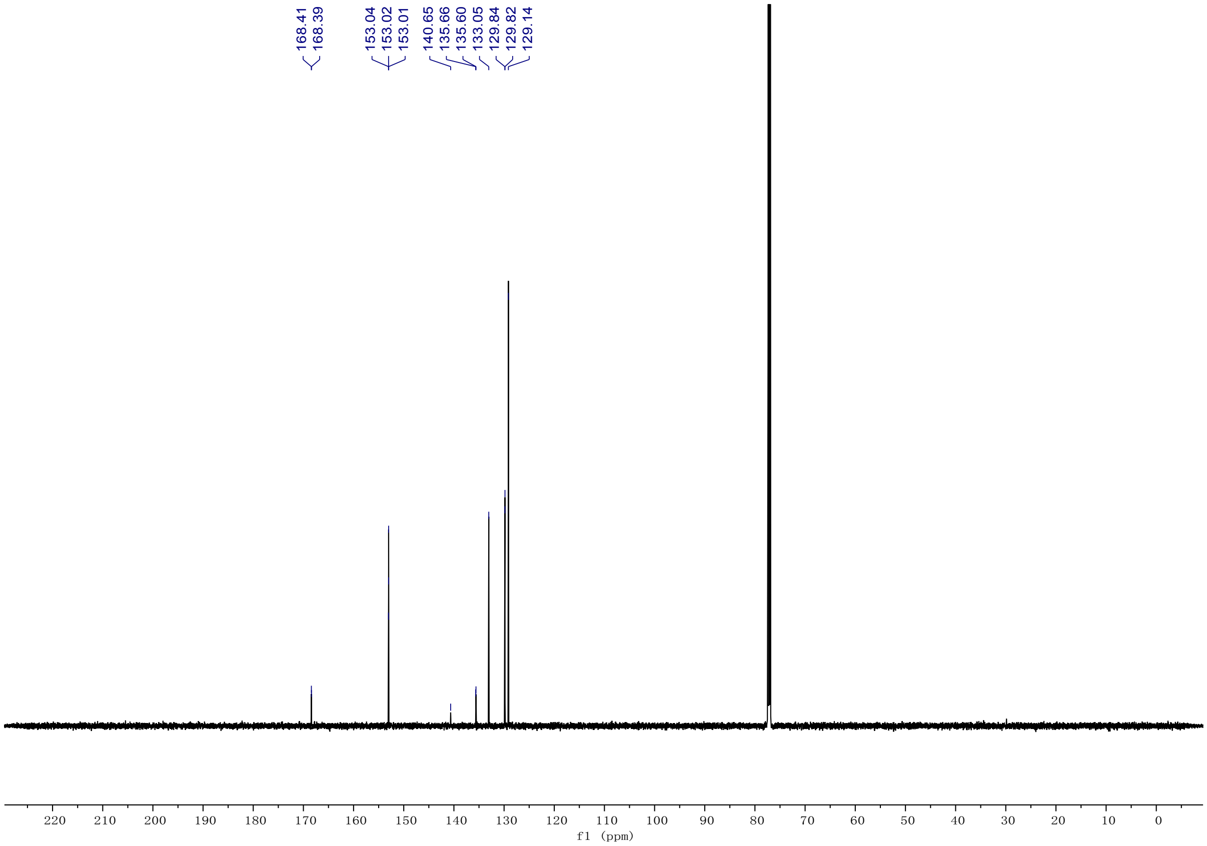
168.41
168.39
153.04
153.02
153.01
140.65
135.66
135.60
133.05
129.84
129.82
129.14
220 210 200 190 180 170 160 150 140 130 120 110 100 90 80 70 60 50 40 30 20 10 0
f1 (ppm)

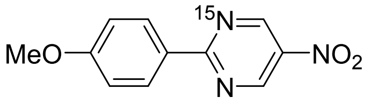

MeO
15N
N
NO2

7d

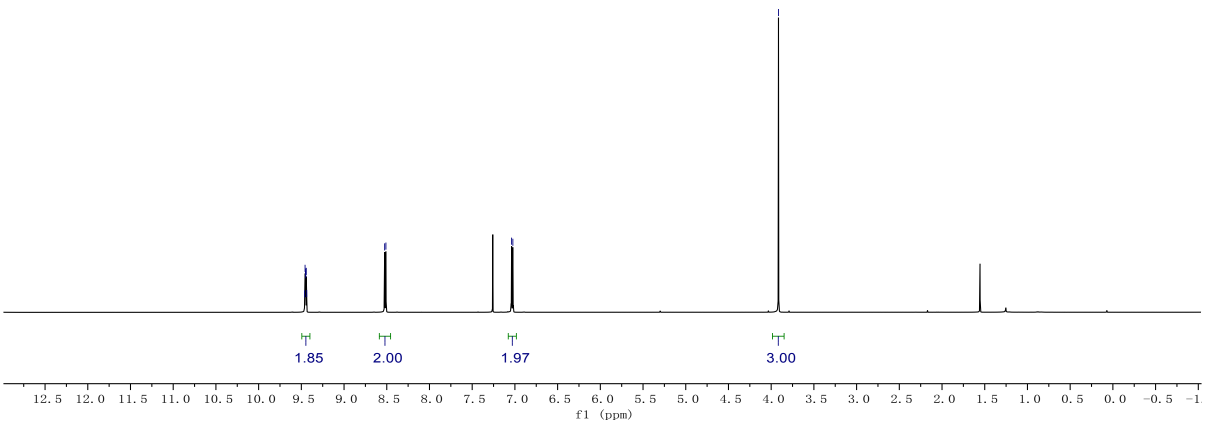

9.46
9.46
9.45
9.45
9.44
9.44
8.52
8.51
7.04
7.02
3.91
1.85
2.00
1.97
3.00
12.5 12.0 11.5 11.0 10.5 10.0 9.5 9.0 8.5 8.0 7.5 7.0 6.5 6.0 5.5 5.0 4.5 4.0 3.5 3.0 2.5 2.0 1.5 1.0 0.5 0.0 -0.5
f1 (ppm)

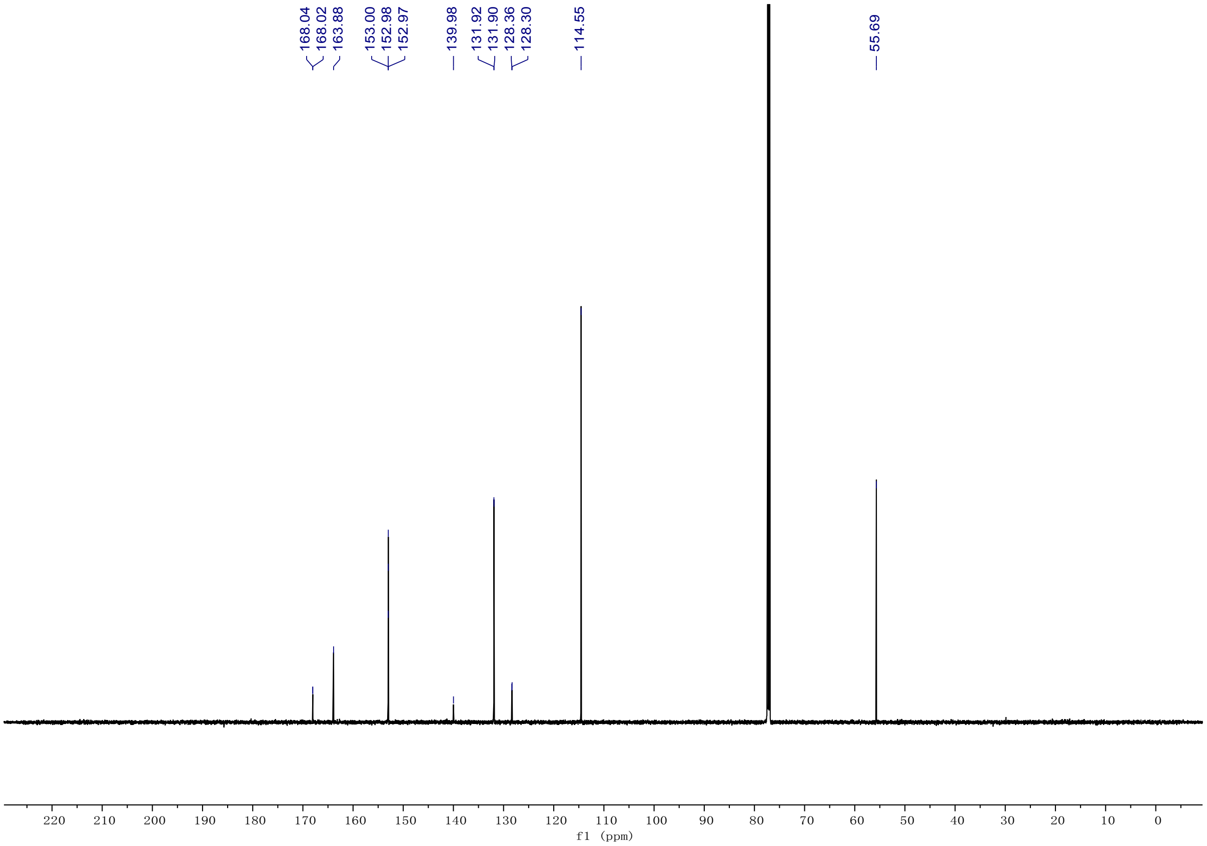

168.04
168.02
163.88
153.00
152.98
152.97
139.98
131.92
131.90
128.36
128.30
114.55
55.69
220 210 200 190 180 170 160 150 140 130 120 110 100 90 80 70 60 50 40 30 20 10 0
f1 (ppm)

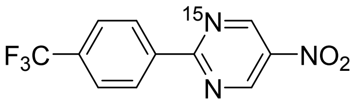
F3C
15N
N
NO2

7e

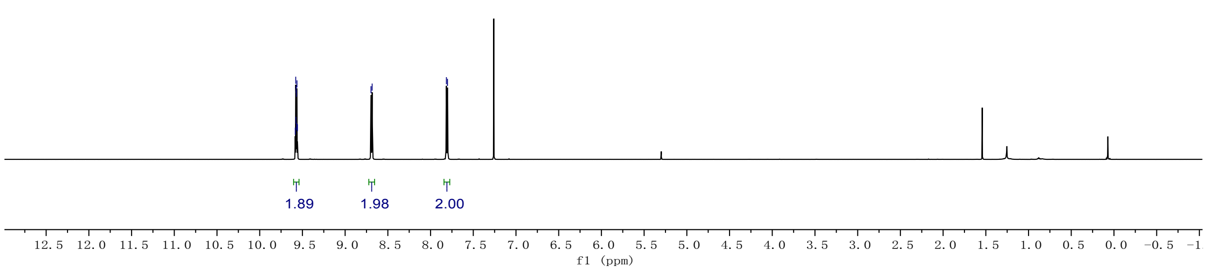
9.58
9.58
9.58
9.57
9.57
9.57
9.56
8.70
8.68
7.81
7.80
1.89
1.98
2.00
f1 (ppm)

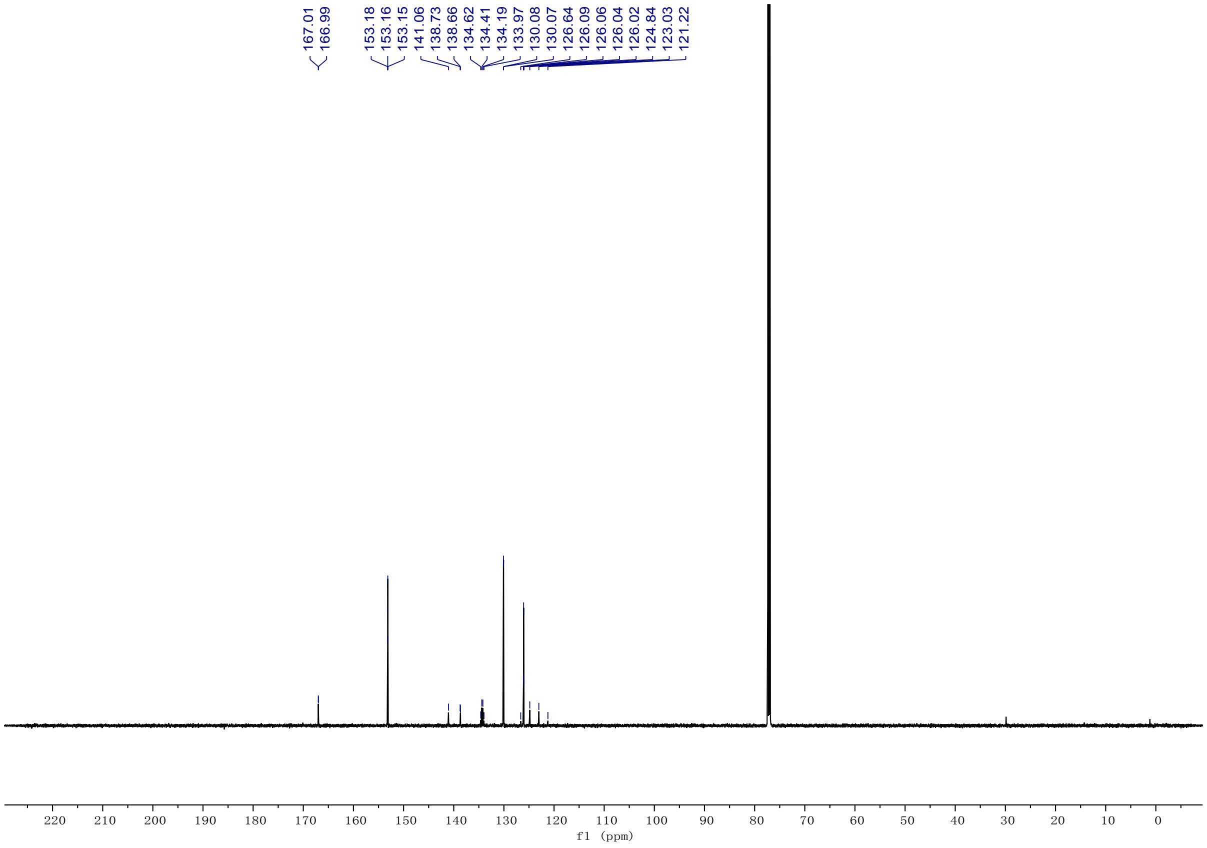
167.01
166.99
153.18
153.16
153.15
141.06
138.73
138.66
134.62
134.41
134.19
133.97
130.08
130.07
126.64
126.09
126.06
126.04
126.02
124.84
123.03
121.22
f1 (ppm)

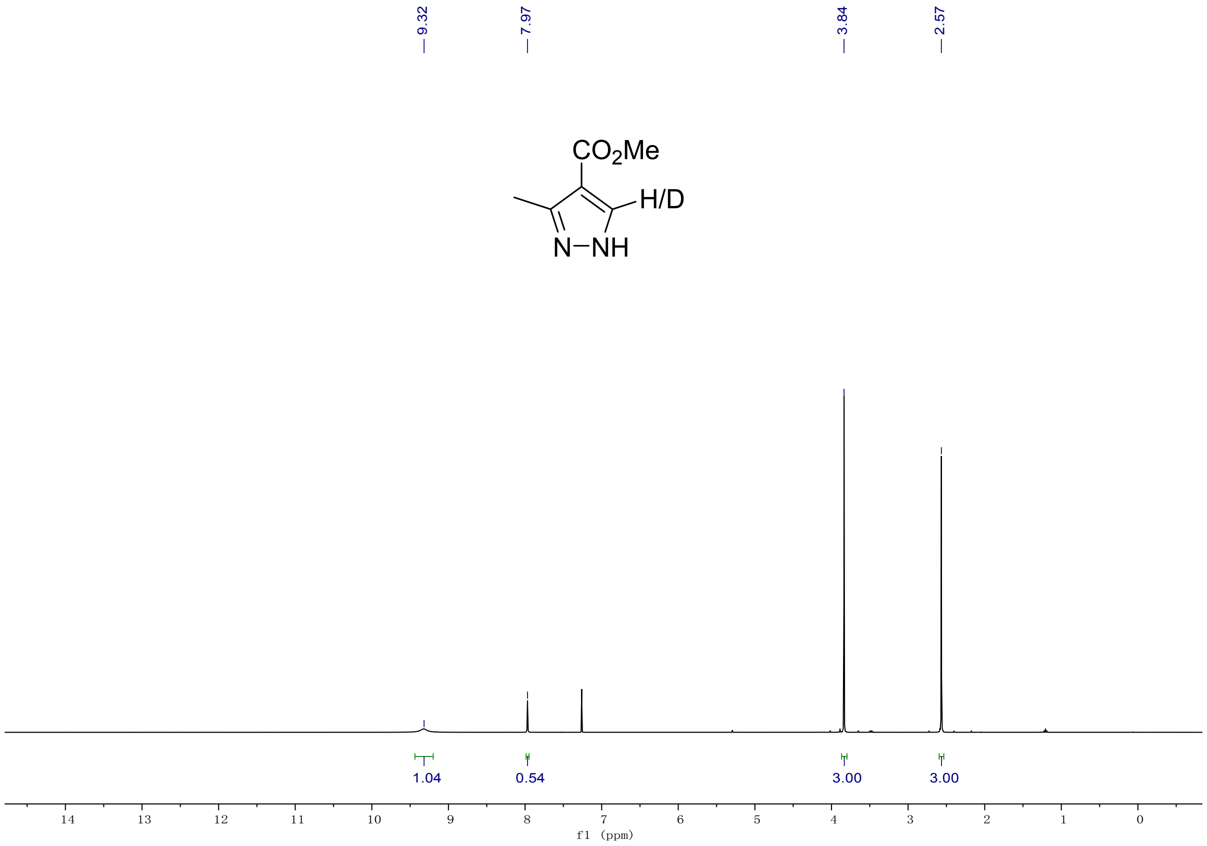

9.32
7.97
3.84
2.57
CO2Me
H/D
N–NH
1.04
0.54
3.00
3.00
14
13
12
11
10
9
8
7
6
5
4
3
2
1
0
f1 (ppm)

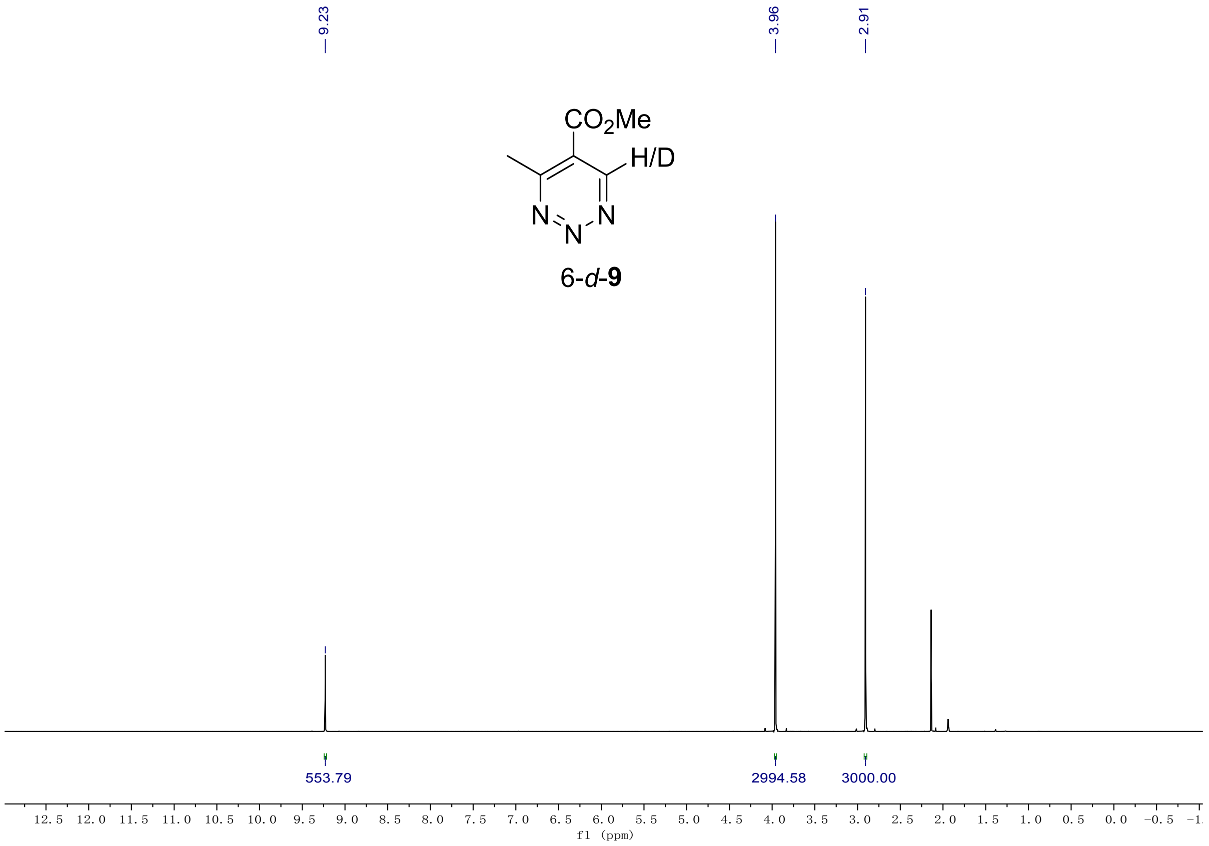

9.23
3.96
2.91
CO2Me
H/D
N
N
N
6-d-9
553.79
2994.58
3000.00
12.5 12.0 11.5 11.0 10.5 10.0 9.5 9.0 8.5 8.0 7.5 7.0 6.5 6.0 5.5 5.0 4.5 4.0 3.5 3.0 2.5 2.0 1.5 1.0 0.5 0.0 -0.5
f1 (ppm)
